# The histone methyltransferase EZH2 primes the early differentiation of follicular helper T cells during acute viral infection

**DOI:** 10.1038/s41423-019-0219-z

**Published:** 2019-03-06

**Authors:** Xiangyu Chen, Guoshuai Cao, Jialin Wu, Xinxin Wang, Zhiwei Pan, Jianbao Gao, Qin Tian, Lifan Xu, Zhirong Li, Yaxing Hao, Qizhao Huang, Pengcheng Wang, Minglu Xiao, Luoyingzi Xie, Shupei Tang, Zhenyu Liu, Li Hu, Jianfang Tang, Ran He, Li Wang, Xinyuan Zhou, Yuzhang Wu, Mengjie Chen, Beicheng Sun, Bo Zhu, Jun Huang, Lilin Ye

**Affiliations:** 1 Institute of Immunology, PLA, Third Military Medical University, 400038 Chongqing, China; 2 Institute for Molecular Engineering, University of Chicago, Chicago, IL 60637 USA; 3 Institute of Cancer, Xinqiao Hospital, Third Military Medical University, 400038 Chongqing, China; 4 Cancer Center, The General Hospital of Western Theater Command, Chengdu, China; 5 Section of Genetic Medicine, Department of Medicine, University of Chicago, Chicago, IL 60637 USA; 6 Department of Hepatobiliary Surgery, The Affiliated Drum Tower Hospital of Nanjing University Medical School, Nanjing, 210009 Jiangsu China

**Keywords:** Lymphocyte differentiation, Infection, Antimicrobial responses

## Abstract

Epigenetic modifications to histones dictate the differentiation of naïve CD4^+^ T cells into different subsets of effector T helper (T_H_) cells. The histone methyltransferase enhancer of zeste homolog 2 (EZH2) has been implicated in the mechanism regulating the differentiation of T_H_1, T_H_2 and regulatory T (T_reg_) cells. However, whether and how EZH2 regulates follicular helper T (T_FH_) cell differentiation remain unknown. Using a mouse model of acute lymphocytic choriomeningitis virus (LCMV) infection, we observed abundant EZH2 expression and associated H3K27me3 modifications preferentially in the early committed virus-specific T_FH_ cells compared to those in T_H_1 cells. Ablation of EZH2 in LCMV-specific CD4^+^ T cells leads to a selective impairment of early T_FH_ cell fate commitment, but not late T_FH_ differentiation or memory T_FH_ maintenance. Mechanistically, EZH2 specifically stabilizes the chromatin accessibility of a cluster of genes that are important for T_FH_ fate commitment, particularly *B cell lymphoma 6 (Bcl6)*, and thus directs T_FH_ cell commitment. Therefore, we identified the chromatin-modifying enzyme EZH2 as a novel regulator of early T_FH_ differentiation during acute viral infection.

## Introduction

During pathogenic infections, follicular T helper (T_FH_) cells provide essential assistance to cognate pathogen-specific B cells that enable them to initiate and sustain germinal center (GC) reactions in B-cell follicles within secondary lymphoid tissues.^1^ GC reactions lead to both the rapid production of high-affinity antibodies that protect against the immediate infection and the subsequent generation of persistent humoral immune memory for long-term protection.^1,2^ In the early stage of GC reactions, intimate interactions between early differentiated T_FH_ cells and newly activated cognate B cells in the interfollicular regions of secondary lymphoid tissues direct the migration of both cell types to B-cell follicles, in turn cooperating to prime early GC responses.^3^ During ongoing GC reactions, T_FH_ cells promote the survival, proliferation, class switching and hypermutation of cognate B cells and eventually drive them to differentiate into long-lived memory B cells and antibody-secreting plasma cells by secreting important cytokines such as IL-21,^4^ IL-4^5^ and IL-9^6^ and engaging certain cell surface-bound receptors and their matching ligands, including CD40-CD40L,^7^ ICOS-ICOSL^7^ and PD-1-PD-L1.^8^ Unlike other lineages of CD4^+^ helper T (T_H_) cells, T_FH_ cells are programmed to express the chemokine receptor CXCR5.^9–11^ In response to the chemoattractant CXCL3,^12,13^ CXCR5^+^ T_FH_ cells migrate to B-cell follicles, where they physically interact with cognate B cells.^1^

Given the critical role of T_FH_ cells in B-cell-mediated humoral immunity, investigations of the early fate commitment of T_FH_ cells are very important, as the differentiation of T_FH_ cells occurs within 48 h after acute viral infections.^14,15^ The transcriptional repressor B cell lymphoma-6 (Bcl-6) functions as a “master regulator” to govern early T_FH_ cell differentiation.^16–18^ A rapid induction of Bcl-6 expression in de novo activated virus-specific CD4^+^ T cells represents a key step toward the T_FH_ fate commitment. A wide variety of transcription factors (TFs) have recently been shown to regulate Bcl-6 expression during T_FH_ differentiation.^3^ STAT family TFs, including STAT1,^19^ STAT3^20^ and STAT4,^21^ promote Bcl-6 expression in response to stimulation with the corresponding cytokines, such as IL-6, IL-12 and IL-21. Batf,^22^ IRF4,^23^ Notch1 and Notch2^24^ have also been reported to induce Bcl-6 expression and promote T_FH_ differentiation. In contrast, the TFs Blimp-1^16^ and Foxo1^25^ inhibit Bcl-6 expression and subsequently repress T_FH_ differentiation. Despite the known positive and negative effects of these TFs on Bcl-6 expression, researchers have not conclusively determined whether these regulators exert their effects during the early stage of T_FH_ commitment. Transcription factor-1 (TCF-1) initiates the T_FH_ fate commitment by directly inducing Bcl-6 expression and suppressing Blimp-1 expression during an acute viral infection.^15,26,27^ Although TCF-1 is expressed at high levels in naïve CD4^+^ T cells,^28^ activated virus-specific CD4^+^ T cells must further increase TCF-1 expression levels for subsequent T_FH_ conversion following an acute viral infection.^15^ Currently, additional factors that are responsible for the early induction of the fate commitment of T_FH_ cells remain to be determined.

Epigenetic modifications of histones have been extensively implicated in the mechanisms regulating T cell differentiation.^29,30^ The epigenetic regulator enhancer of zeste homolog 2 (EZH2), which is the catalytic subunit of polycomb repressive complex 2 (PRC2), functions as a methyltransferase to induce the trimethylation of histone H3 at lysine 27.^31,32^ The resulting H3K27me3 protein recruits chromatin-compressing protein complexes to certain loci to silence the expression of corresponding genes.^31,32^ EZH2-mediated H3K27me3 plays a critical role in the differentiation and lineage stability of various types of CD4^+^ T_H_ cells, including T_H_1, T_H_2 and regulatory T (T_reg_) cells.^33–38^ In the T_H_1 and T_H_2 lineages, several groups reported that EZH2 and the associated H3K27me3 directly bind to *Tbx21*^36,38^ (which encodes the TF T-bet, a master regulator of T_H_1 differentiation) and *Gata3*^36^ (encoding the TF Gata3, which specifies T_H_2 differentiation) to inhibit the transcription of both TFs and eventually suppress the differentiation of naïve CD4^+^ T cells into both T_H_1 and T_H_2 cells. Additionally, EZH2 and H3K27me3 inhibit the differentiation of T_H_1 and T_H_2 cells through the direct epigenetic marking and silencing of genes that encode lineage-specific cytokines, such as *Ifng*^36,38^ and *Il13*.^36,38^ Paradoxically, EZH2 also induces T_H_1 cell differentiation by increasing the stability of T-bet and inducing the production of T_H_1 cytokines.^34,35^ In T_reg_ cells, CD28-induced EZH2 expression promotes H3K27me3 deposition at gene loci that are normally repressed in Treg cells and thus plays an important role in stabilizing the lineage specification of activated T_reg_ cells.^33^ Despite the profound effects of EZH2 on T_H_1, T_H_2 and T_reg_ differentiation, whether and how EZH2 regulates the T_FH_ fate commitment remains to be investigated.

Here, we first defined a strict lineage-specific mode of chromatin accessibility in virus-specific T_FH_ cells compared to virus-specific T_H_1 cells in response to an acute infection. Bona fide differentiated virus-specific T_FH_ cells exhibited increased EZH2 expression and the associated H3K27me3 modification compared to naïve T cells and T_H_1 cells in the early days after an acute lymphocytic choriomeningitis virus (LCMV) infection. Furthermore, EZH2 was required for governing the chromatin accessibility of a cluster of T_FH_-lineage-associated genes, particularly *Bcl6*, which are essential for T_FH_ fate commitment. Accordingly, inactivation of EZH2 in virus-specific CD4^+^ T cells led to a pronounced reduction in the early commitment but not late differentiation or maintenance of T_FH_ cells.

## Materials and methods

### Mice, virus, bacteria and tamoxifen treatment

CD45.1^+^ SMARTA mice were a gift from Dr. R. Ahmed (Atlanta, USA). The *Ezh2*^*fl/fl*^, *Cd4*-Cre transgenic, ERT2-Cre transgenic and C57BL/6J (CD45.1 and CD45.2) mice were obtained from Jackson Laboratories. The LCMV Armstrong strain was provided by Dr. R. Ahmed (Atlanta, USA), and 2 × 10^5^ plaque-forming units (PFUs) of this strain were intraperitoneally injected into mice to establish an acute viral infection. *Listeria monocytogenes* expressing the LCMV glycoprotein-specific I-A^b^-restricted CD4^+^ T cell epitope GP61–80 (LM-GP61) was created from a vector strain,^39^ and 1 × 10^7^ colony-forming units (CFUs) of the recombinant bacteria were intravenously injected to establish a bacterial infection in mice. Six- to ten-week old mice of both sexes were infected without randomization or “blinding”. Bone marrow (BM) chimera mice were infected 2 months after reconstitution. Tamoxifen (T5648; Sigma-Aldrich; 10 mg/ml) in sunflower oil (S5007; Sigma-Aldrich) was intraperitoneally injected into mice at a daily dose of 1 mg/mouse for 4 days. Infected mice were housed in accordance with the institutional biosafety regulations of the Third Military Medical University. All mouse experiments were performed according to the guidelines of the Institutional Animal Care and Use Committees of the Third Military Medical University.

### ATAC-Seq library preparation

The ATAC-Seq libraries were prepared as previously described.^40^ Briefly, 50,000 target cells were washed with PBS and then treated with lysis buffer, followed by labeling with the Nextera enzyme (15027865; Illumina). The labeled samples were immediately amplified by 9–10 cycles of polymerase chain reaction (PCR) with barcoded primers and sequenced with a HiSeq4000 instrument in a 150 bp/150 bp paired-end run or a NextSeq500 instrument in a 76 bp/76 bp paired-end run.

### ATAC-Seq data preprocessing

Raw sequencing reads were first trimmed of adapters to improve the quality using Trim Galore! v0.4.4 (https://www.bioinformatics.babraham.ac.uk/projects/trim_galore/), which is a wrapper based on CutAdapt v1.14 (ref. ^41^) and FastQC v0.11.5 (https://www.bioinformatics.babraham.ac.uk/projects/fastqc/). Paired-end reads that passed quality control (QC) were then aligned to mm10 using Bowtie2 v2.2.9 (ref. ^42^). The resulting BAM files were then filtered again to remove unmapped reads, mate-unmapped reads, nonprimary aligned reads, reads that failed platform quality checks and PCR duplicate reads using SAMtools v1.4.1 (ref. ^43^) (-F 1804). In addition, reads mapped to ChrM were also removed and PCR duplicate reads were further identified and removed using Picard v2.16.0 MarkDuplicate (https://broadinstitute.github.io/picard/).

The insert size distributions were then calculated using Picard v2.16.0 CollectInsertSizeMetrics. Since Tn5 transposase binds as a dimer and inserts two adaptors separated by 9 bp,^44^ all aligned reads were shifted by + 4 bp on the positive strand and −5 bp on the negative strand using deepTools v2.5.2 alignmentSieve.^45^ Afterward, peak calling was performed using MACS2 v2.1.1,^46^ with a *q*-value threshold of 0.01. Peaks that overlapped with the Encode black list regions were then removed using bedtools intersect v2.26.0 (ref. ^47^). We then merged peaks from all replicates and filtered peaks that are not reproducible, based on the Irreproducible Discovery Rate (IDR)^48^ (IDR < 0.005), across at least one pair of replicates in each sample group. Subsequently, depending on the comparisons required for different purposes, filtered peaks from multiple sample groups were merged using the bedtools v2.26.0 merge algorithm to create genome-wide atlas of accessible chromatin regions for further analysis.

### Assignment of ATAC-Seq peaks to genes

Each ATAC-Seq peak was uniquely assigned to a gene, with the corresponding feature annotations (e.g., exon, intron and intergenic) in R v3.4.1 using ChipPeakAnno v3.10.2,^49^ with TxDb.Mmusculus.UCSC.mm10.knownGene v3.4.0 as the reference database. The following rules listed in order of priority were used for assignment: peaks that overlap with the transcribed region of a gene or within 2 kb upstream or downstream of the transcribed region are assigned to that gene; any remaining peaks are assigned to the nearest gene based on the distance from the center of the peak to the transcription start site (TSS) or 3′ end of the transcribed region. Regions within 5 kb of the TSS are defined as the promoter regions.

### Differential peak analysis and principal component analysis

For each analysis that compares data from multiple sample groups, all required shifted BAM files from all replicates of those samples were used to generate an accessibility matrix by counting the normalized reads (normalized by DESeq2-calculated size factors) within each peak region of the corresponding atlas peak file using deepTools v.2.5.4 multiBamSummary in BED-mode. The resulting matrix was input to DESeq2 v1.16.1 (ref. ^50^) to calculate the differential accessibility of the peaks of the relevant pairs. Principal component analysis (PCA) plots were then generated using DESeq2 v1.16.1.

### Coverage plots and chromatin accessibility heat map

For each sample group, shifted and RPM normalized bam files were first converted to bedgraph files by counting the normalized reads in 10 bp bins and removing reads in the Encode blacklisted regions. Then, reads obtained from replicates of the same sample group were pooled using bedtools v2.26.0 unionbedg, and the resulting merged bedgraph files were used to generate a single coverage plot for each sample group, which was visualized in IGV v2.4.4 (ref. ^51^) and used to generate the chromatin accessibility heat map. Using deepTools v2.5.4 computeMatrix, each atlas peak was extended to a ±1 kb region from the peak center, and the reads from the final coverage plot were then separated into 10 bp bins to be represented as a row in the heat map. Selected differential peaks were then stacked together to generate the overall heat map using deepTools v2.5.4 plotHeatmap. Bins with read counts greater than a threshold, which was defined as the 75th percentile +1.5 × inter quartile range (IQR), were capped at that threshold value to increase the visibility of low-signal regions. Capping was only performed to plot the heat map, and all other analyses were performed with uncapped values.

### Flow cytometry and antibodies

Flow cytometry data were obtained with a FACSCanto II instrument (BD Biosciences) and analyzed using FlowJo software (Tree Star). The major histocompatibility complex (MHC) class II (I-A^b^) tetramer of LCMV epitope of GP66–77 was provided by Dr. Rafi Ahmed (Emory University). The antibodies and reagents used for flow cytometry are listed in Supplementary Table 3. CXCR5 staining has been described previously.^15^ Surface staining was performed in PBS containing 2% fetal bovine serum (wt/vol) on ice. Intracellular staining of Foxp3, EZH2, H3K27me3, TCF-1 and Bcl-6 was performed using the Foxp3/Transcription Factor Staining Buffer Set (00–5523; eBioscience). MHC II GP66–77 tetramer staining was performed by incubating cells with the tetramer for 1 h at 37 °C.

### Immunofluorescence staining

Immunofluorescence staining was performed using previously described methods.^52^ Briefly, frozen sections of the spleen were fixed with cold acetone for 10 min at 4 °C and blocked with 5% normal goat serum for 30 min. Sections were then stained with biotin-conjugated PNA (RL-1072; Vector), Alexa Fluor 647-conjugated IgD (11–26c.2a; eBioscience) and PE-conjugated CD4 (RM4–5; BD Biosciences) antibodies, followed by Alexa Fluor 488-conjugated streptavidin (25–4317–82; Invitrogen). Cover slips on which both types of cells were cultured were mounted on slides with the ProLong Antifade Kit (P-7481; Life Technologies) and examined under a Zeiss LSM 510 confocal fluorescence microscope. The images were processed with ImageJ software.

### Retroviral constructs and transduction

The sequences encoding the codon-improved Cre (iCre) gene or *Bcl6* were amplified and cloned into the vector MIGR1 (MSCV-IRES-GFP) or MIGR2 (MSCV-IRES-hCD2), respectively. Retroviruses were packaged by transfecting 293T cells with the retroviral vectors along with the pCL^eco^ plasmid. SMARTA cells were activated in vivo by injecting 200 μg of the GP61–77 peptide into SMARTA mice. Eighteen hours later, activated SMARTA cells were purified and “spin-infected” by centrifugation (800 g) with retrovirus supernatants, 20 ng/ml IL-2 (130–098–221; Miltenyi Biotec) and 8 μg/ml polybrene (H9268; Sigma-Aldrich) at 37 °C for 90 min. SMARTA cells were then transferred into recipient mice, followed by the infection of the hosts with LCMV Armstrong.

### Adoptive transfer

A total of 5 × 10^5^ (for analysis on days 2, 3 or 5) or 1 × 10^4^ (for analysis on day 8 or later) CD45.1^+^ SMARTA cells (naïve or retrovirus-transduced) were adoptively transferred into CD45.2^+^ recipients. On the following day, the recipients were intraperitoneally injected with 1 × 10^6^ PFUs of LCMV Armstrong (day 2 or 5) or 1 × 10^7^ CFUs of LM-GP66 (day 3) or were intraperitoneally injected with 2 × 10^5^ PFUs of LCMV Armstrong (day 8 or later). For the EPZ6438-treated SMARTA cell transfer experiment, naïve CD45.1^+^ SMARTA cells were treated with EPZ6438 (2 μM; E-7438, Active Biochem) or vehicle at 37 °C for 3 days, and then transferred into CD45.2^+^ recipient mice, followed by infection with LCMV Armstrong.

### BM chimeras

A total of 2 × 10^6^ BM cells harvested from *Ezh2*^fl/fl^ERT2-Cre (CD45.2^+^) mice and C57BL/6J wild-type (WT) (CD45.1^+^) mice were mixed at a ratio of 4:6. Mixed BM cells were then intravenously injected into lethally irradiated (two doses of 550 rads each) CD45.1^+^ WT recipients. After a 2-month reconstitution, the recipient mice were infected with LCMV Armstrong.

### ELISA

The LCMV-specific serum IgG titers were measured using an ELISA, as previously described.^53,54^ Briefly, plates were coated with LCMV-infected cell lysates, and LCMV-specific antibodies were detected with HRP-conjugated goat anti-mouse IgG secondary antibodies (Southern Biotech).

### Microarray

T_FH_ cells were isolated using a previously described method.^15^ Briefly, total splenocytes obtained from WT and *Ezh2*^fl/fl^*Cd4*-Cre mice on day 8 after LCMV Armstrong infection were subjected to the depletion of lineage marker-positive cells (Lin^+^ cells) using biotin-conjugated antibodies (anti-B220 (RA3–6B2), anti-CD8 (53.6.7), anti-CD11c (N418), anti-TER-119 (TER-119) and anti-NK1.1 (PK136); all from BioLegend), followed by coupling to BeaverBeads Mag500 Streptavidin (22302; Beaver). The enriched Lin^−^ cells were then stained with anti-CD4, anti-CD44, anti-GITR, anti-CD25 and anti-CXCR5 antibodies (all identified in Supplementary Table 3). The CD4^+^CD25^−^GITR^−^CD44^+^CXCR5^+^ T_FH_ cells were sorted with a FACS Aria II cell sorter (BD Biosciences) and then immediately lysed with TRIzol LS reagent (10296; Life Technologies). Then, total RNA was extracted and submitted to CapitalBio for a microarray analysis.

### Quantitative reverse-transcription PCR

Sorted T_FH_ cells were sorted directly into TRIzol LS reagent (10296; Life Technologies). Total RNA was extracted and reverse transcribed with the RevertAid Minus First Strand cDNA Synthesis Kit (K1632; Thermo Scientific). The relative expression of various genes was determined using AceQ qPCR SYBR Green Master Mix (Q111; Vazyme) with a CFX96 Touch Real-Time System (Bio-Rad). The primers for the indicated genes are summarized in Supplementary Table 4.

### ChIP

ChIP assays of sorted T_FH_ cells, T_H_1 cells and naïve CD4^+^ T cells were performed using a Simple Enzymatic Chromatin IP Kit (Magnetic Beads) (9003; Cell Signaling Technology). The resulting chromatin fragments were immunoprecipitated with an anti-H3K27me3 antibody (9733; Cell Signaling Technology), followed by binding to ChIP Grade Protein G Magnetic Beads (9006; Cell Signaling Technology) and purification with a PCR purification kit (28104; Qiagen). Sequence-indexed libraries were prepared from immunoprecipitated chromatin fragments using the Illumina TruSeq indexed pair-ended DNA library preparation protocol and ultimately sequenced using the NextSeq500 platform.

### ChIP-Seq analysis and coverage plot

The raw sequencing reads obtained from the Chip-Seq analysis were first trimmed and filtered with Trim Galore! v0.4.4, and then aligned in single-end mode and searched against mm10 using Bowtie2 v2.2.9. The reads were then filtered using samtools to remove low-quality reads and unmapped reads. Duplicate reads were also filtered using Picard v2.16.0 MarkDuplicates. Peak calling was performed using MACS2 v2.1.1 with a *q*-value threshold of 0.05 and the --SPMR flag. The resulting bedgraph files were used to build a coverage plot using MACS2 v2.1.1 bdgcmp with the logLR method and a *P*-value of 1e−5.

### Statistical analysis

Statistical analyses were conducted with Prism 6.0 software (GraphPad). An unpaired two-tailed *t*-test with a 95% confidence interval was used to calculate *P*-values. For retroviral transduction, SMARTA cell cotransfer, spleen chimera and BM chimera experiments, and a paired two-tailed *t*-test with the 95% confidence interval were used to calculate *P-*values.

## Results

### Chromatin states of virus-specific T_FH_ and T_H_1 cells in response to acute viral infection

**Fig. 1:**
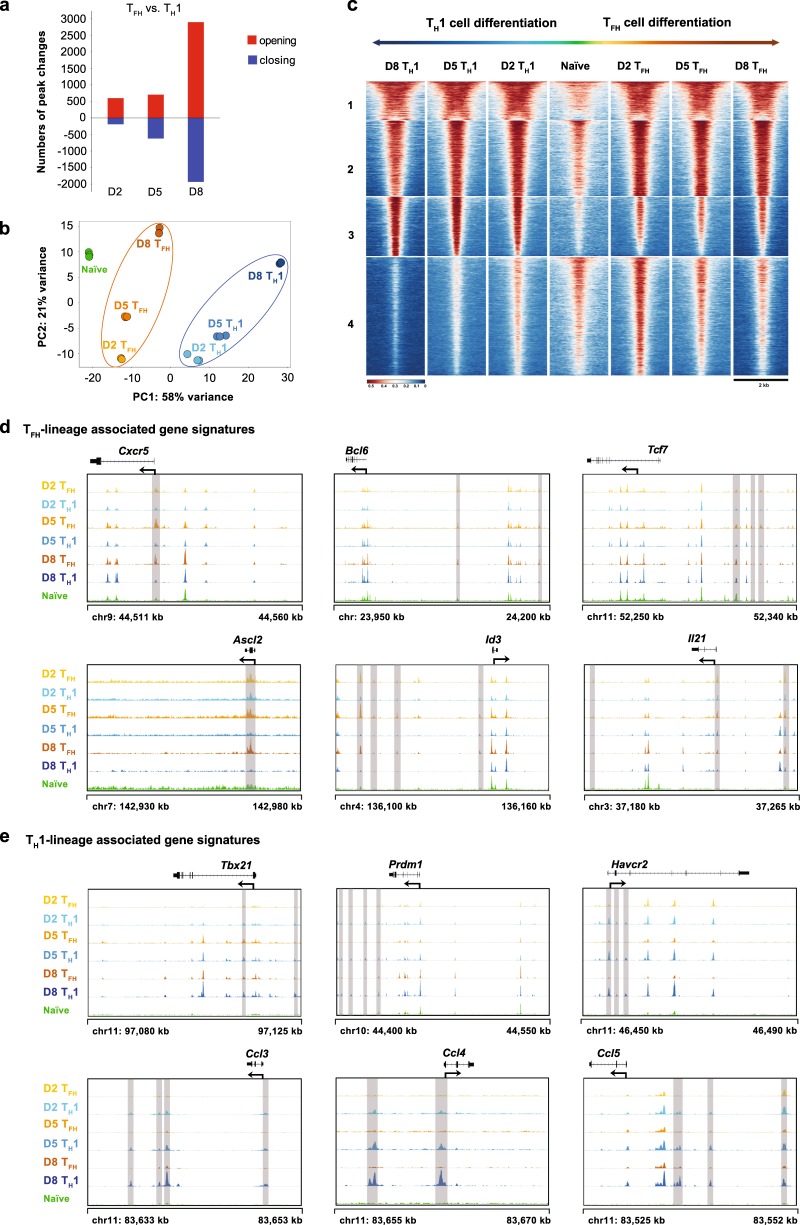
Chromatin states of the virus-specific T_FH_ and T_H_1 cells in response to an acute viral infection. **a** Numbers of chromatin peaks with differential accessibility (FDR < 0.05; FC > 4) between SMARTA T_FH_ cells and SMARTA T_H_1 cells at the indicated time points after LCMV Armstrong infection. **b** PCA plot of the peak accessibilities in naïve SMARTA CD4^+^ T cells, SMARTA T_FH_ cells (days 2, 5 and 8 postinfection) and SMARTA T_H_1 cells (days 2, 5 and 8 postinfection). Each dot represents a replicate of the indicated group. **c** Chromatin accessibility heat map of differential peaks from **a**. Each row represents one of the 15,600 differential peaks that was center-aligned and extended upstream and downstream by 1 kb from the center. The peaks are *K*-means clustered. **d** ATAC-Seq signal profiles of T_FH_ lineage-associated gene loci. **e** ATAC-Seq signal profiles of T_H_1 lineage-associated gene loci. Differential peaks are highlighted in gray (**d** and **e**). The data were obtained from one experiment with three biological replicates (pooled from at least five mice per group) (**a**–**e**)

In response to an acute viral infection, activated virus-specific CD4^+^ T cells differentiate into either T_H_1 or T_FH_ cells.^15,55,56^ We first adoptively transferred LCMV-specific naïve SMARTA cells (expressing a transgenic T cell receptor specific for the LCMV glycoprotein epitope I-A^b^GP66–77) into WT C57BL/6J recipients and subsequently infected the chimeric recipients with the LCMV Armstrong strain to investigate the potential regulatory regions involved in this bifurcated differentiation at the genome level. Then, we sorted virus-specific SMARTA T_FH_ cells and T_H_1 cells from the SMARTA chimera mice on days 2, 5 and 8 after the infection (Supplementary Figure S1a–c) and subsequently performed an ATAC-Seq assay to measure the transposase-accessible chromatin.^40^ We also sorted naïve SMARTA cells (CD4^+^CD25^−^CD62L^+^CD44^−^) as a control (Supplementary Figure S1d). QC of the ATAC-Seq libraries revealed the characteristic DNA fragment length distribution and the expected peak distribution across genomic features (Supplementary Figure S2a, b). With these high-quality libraries, we assessed the differences in chromatin-accessible regions (ChARs) and found that compared to naïve cells, dramatic changes in the numbers of ChARs emerged as early as day 2 postinfection in both virus-specific T_FH_ and T_H_1 cells (Supplementary Figure S2c). Furthermore, chromatin-accessible patterns were discerned in T_FH_ and T_H_1 cells, respectively, at different time points postinfection (Fig. 1a). The greatest differences in ChAR patterns were observed between T_FH_ and T_H_1 cells at day 8 postinfection (Fig. 1a), reflecting the multistage differentiation of fully functional T_FH_ and T_H_1 cells.^1^ A ChAR-based PCA further revealed that T_FH_ and T_H_1 cells started to enter two distinct CD4^+^ T cell differentiation states on day 2 postinfection, and these differentiation trajectories continuously developed through the entire process (Fig. 1b).

*K*-means clustering of the differential peaks revealed four distinct areas, and the third and fourth clusters (gene lists are provided in Supplementary Tables S1 and S2) revealed opened ChARs that were specifically detected in T_H_1 and T_FH_ cells, respectively (Fig. 1c), compared to naïve cells. Notably, when focusing on cluster 4, we found an array of T_FH_ lineage-associated gene loci that were enriched in this region, including *Bcl6*, *Tcf7*, *Id3*,^57^ *Ascl2*,^58^ *Cxcr5* and *Il21* (Fig. 1d). The T_H_1-associated genes *Tbx21*, *Prdm1*, *Havcr2*, *Ccl3*, *Ccl4* and *Ccl5* were observed in cluster 3 (Fig. 1e). Further analysis of the ChARs for each individual gene locus revealed the stringent lineage-specific mode of chromatin accessibility; i.e., the chromatin accessibility of T_FH_-associated genes was more prominent in T_FH_ cells than in T_H_1 cells, and vice versa (Fig. 1d and e). Based on these results, chromatin remodeling is tightly associated with the T_FH_ but not T_H_1 lineage commitment and differentiation in response to an acute viral infection.

### Dynamic EZH2 expression and H3K27me3 modification in virus-specific T_FH_ cells

**Fig. 2:**
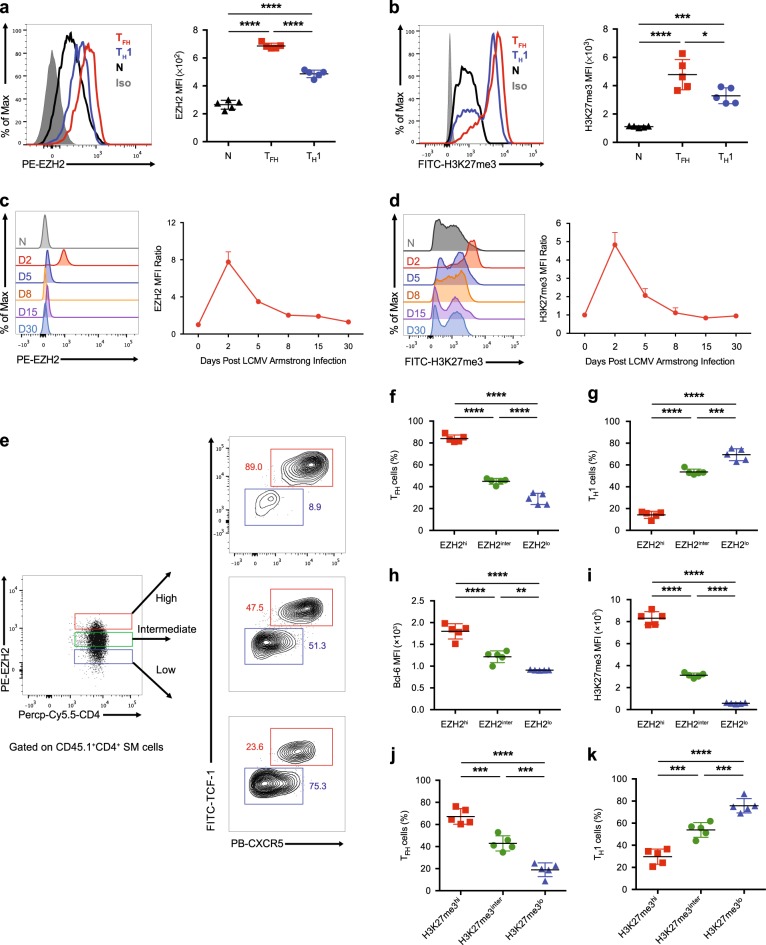
Dynamic changes in EZH2 expression and the H3K27me3 modification in virus-specific T_FH_ cells. **a**, **b** Comparison of EZH2 (**a**) and H3K27me3 (**b**) levels between SMARTA T_FH_ cells (CD25^lo^CXCR5^+^) and SMARTA T_H_1 cells (CD25^hi^CXCR5^−^) from the spleens of CD45.2^+^ wild-type mice that underwent adoptive transfer of CD45.1^+^ SMARTA cells and analyzed on day 2 after LCMV Armstrong infection and in naïve (CD44^lo^CD62L^hi^) SMARTA cells (N). **c**, **d** Flow cytometry analysis of EZH2 (**c**) and H3K27me3 (**d**) levels in T_FH_ cells derived from the mice listed in **a** on days 2, 5, 8, 15 and 30 after the LCMV Armstrong infection and in naïve SMARTA cells (N). The EZH2 or H3K27me3 mean fluorescence intensity (MFI) ratio at each time point was calculated as the MFI of T_FH_ cells / MFI of CD4^+^CD44^lo^ T cells in an identical mouse. **e** Flow cytometry analysis of the EZH2^hi^, EZH2^inter^ and EZH2^lo^ subsets of T_FH_ cells and T_H_1 cells from the mice shown in **a** on day 2 after the LCMV Armstrong infection. The proportions of T_FH_ cells and T_H_1 cells and the MFI of Bcl-6 in each population are summarized in **f**, **g** and **h**, respectively. **i** The MFI of H3K27me3 in the EZH2^hi^, EZH2^inter^ and EZH2^lo^ subsets of T_FH_ cells described in **e**. The proportions of T_FH_ cells (**j**) and T_H_1 cells (**k**) among the H3K27me3^hi^, H3K27me3^inter^ and H3K27me3^lo^ subsets of SMARTA cells from mice shown in **a** on day 2 after the LCMV Armstrong infection. *P < 0.05, ***P < 0.001 and ****P < 0.0001 (unpaired two-tailed *t*-test). The data are representative of two independent experiments with at least four mice per group (**a**–**d** and **f**–**k**; error bars in **a**–**d** and **f**–**k** indicate the s.d.)

The EZH2-mediated H3K27me3 modification plays a critical role in chromatin remodeling.^59^ Next, we sought to investigate the relationship between EZH2 expression and H3K27me3 modification during T_FH_ cell differentiation in response to an acute viral infection. We first adoptively transferred naïve SMARTA cells into WT C57BL/6J recipients, followed by an infection with the LCMV Armstrong strain. On day 2 after infection, we compared EZH2 expression and H3K27me3 modification between naïve (CD44^lo^CD25^−^), T_H_1 (CD25^hi^CXCR5^−^) and T_FH_ (CD25^lo^CXCR5^+^) SMARTA cells. We observed the highest levels of EZH2 expression in T_FH_ cells compared to those in T_H_1 and naïve cells, and T_H_1 cells expressed a relatively higher level of EZH2 than the naïve cells (Fig. 2a). This phenotypic pattern was consistent with the levels of the EZH2-mediated H3K27me3 modification in T_FH_, T_H_1 and naïve SMARTA cells (Fig. 2b). Next, we analyzed the kinetics of EZH2 expression in T_FH_ SMARTA cells at different time points after infection and found that virus-specific T_FH_ cells rapidly increased EZH2 expression upon infection and reached a peak on day 2 postinfection (Fig. 2c). EZH2 expression then decreased to a comparable level to naïve cells on day 8 postinfection (Fig. 2c). Consistent with the EZH2 expression kinetics, the direct target of EZH2, H3K27me3, exhibited similar kinetics during T_FH_ cell differentiation (Fig. 2d).

The fate commitment of virus-specific T_FH_ cells occurs within the first 48 h after an acute viral infection. Therefore, the high expression of EZH2 in early differentiated T_FH_ cells on day 2 postinfection prompted us to examine whether the level of EZH2 expression correlated with T_FH_ cell commitment. For this purpose, on day 2 after infection, we divided SMARTA cells into three subsets, EZH2^hi^, EZH2^inter^ and EZH2^lo^, according to their EZH2 expression levels and observed that the EZH2^hi^ subset was much more poised to adopt the T_FH_ fate than the EZH2^inter^ and EZH2^lo^ subsets (Fig. 2e, f), while the opposite phenotype was observed during T_H_1 differentiation (Fig. 2e–g). Compared to the EZH2^inter^ and EZH2^lo^ subsets, the EZH2^hi^ subset expressed a significantly higher level of Bcl-6 (Fig. 2h), suggesting the enhanced propensity of this subset to become T_FH_ cells. Consistent with these findings, the extent of the H3K27me3 modification was also positively correlated with EZH2 expression and an early T_FH_ fate choice in SMARTA cells on day 2 after an acute viral infection (Fig. 2i–k). Collectively, high EZH2 expression and the associated H3K27me3 modification in activated virus-specific CD4^+^ T cells preferentially drove them to differentiate into T_FH_ cells during the early response to acute viral infection, while the cells displaying low EZH2 and H3K27me3 levels were more prone to differentiate into T_H_1 cells.

### Role of EZH2 in early T_FH_ commitment during acute viral infection

**Fig. 3:**
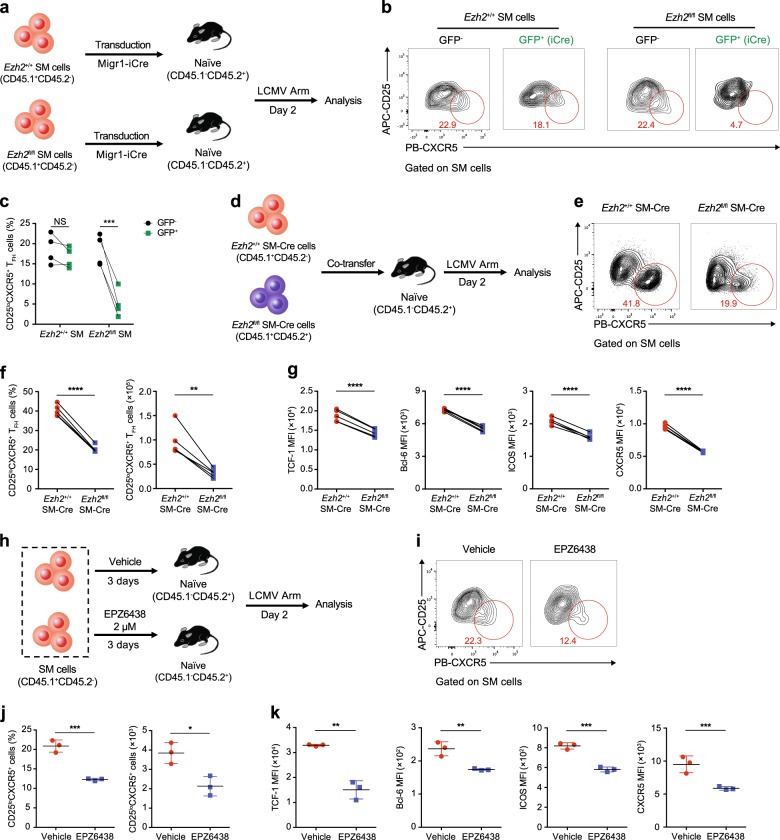
Role of EZH2 in early T_FH_ commitment during an acute viral infection. **a** Experimental setup. A retrovirus overexpressing iCre was introduced into CD45.1^+^*Ezh2*^+/+^ SMARTA (SM) cells and CD45.1^+^*Ezh2*^fl/fl^ SM cells, which were transferred into CD45.2^+^ WT recipients. Then, the recipients were infected with the LCMV Armstrong strain and analyzed on day 2 postinfection. **b** Flow cytometry analysis of *Ezh2*^+/+^ and *Ezh2*^fl/fl^ SM cells transduced with a retrovirus expressing iCre. The numbers adjacent to the outlined areas indicate the percentages of CD25^lo^CXCR5^+^ T_FH_ cells, which are summarized in **c**. **d** Experimental setup. *Ezh2*^+/+^*Cd4*-Cre SMARTA cells (*Ezh2*^+/+^ SM-Cre; CD45.1^+^CD45.2^−^) and *Ezh2*^fl/fl^*Cd4*-Cre SMARTA cells (*Ezh2*^fl/fl^ SM-Cre; CD45.1^+^CD45.2^+^) were cotransferred into WT recipients (CD45.1^−^CD45.2^+^), which were infected with the LCMV Armstrong strain and assessed on day 2 postinfection. **e** Flow cytometry analysis of *Ezh2*^+/+^ SM-Cre and *Ezh2*^fl/fl^ SM-Cre SMARTA cells. The numbers adjacent to the outlined areas indicate the percentages of CD25^lo^CXCR5^+^ T_FH_ cells, which are summarized in **f** (left panel). Total numbers of CD25^lo^CXCR5^+^ T_FH_ cells in **e** are presented (**f**, right panel). **g** Quantification of TCF-1, Bcl-6, ICOS and CXCR5 levels in the CD25^lo^CXCR5^+^ T_FH_ cells shown in **e**. **h** Experimental setup. After treatment with either EPZ6438 or vehicle for 3 days, CD45.1^+^SMARTA cells were transferred into WT CD45.2^+^ recipients that were subsequently infected with the LCMV Armstrong strain. The adoptively transferred SMARTA cells were analyzed on day 2 postinfection. **i** Flow cytometry analysis of EPZ6438-treated and vehicle-treated SMARTA cells. The numbers adjacent to the outlined areas indicate the proportions of CD25^lo^CXCR5^+^ T_FH_ cells, which are summarized in **j** (left panel). Total numbers of CD25^lo^CXCR5^+^ T_FH_ cells analyzed in **i** are presented in **j** (right panel). **k** Quantification of TCF-1, Bcl-6, ICOS and CXCR5 levels in the CD25^lo^CXCR5^+^ T_FH_ cells shown in **j**. NS not significant; *P < 0.05, **P < 0.01, ***P < 0.001 and ****P < 0.0001 (paired two-tailed *t*-test (**c**, **f** and **g**) or unpaired two-tailed *t*-test (**j** and **k**)). The data are representative of two independent experiments with at least three mice (**c**, **f**, **g**, **j** and **k**) per group (error bars in **j** and **k** indicate the s.d.)

Considering the positive correlation between the EZH2 expression level and T_FH_ commitment, we next investigated whether EZH2 is indeed essential for early T_FH_ differentiation upon acute viral infection. We crossed mice harboring *lox*P-flanked *Ezh2* alleles (*Ezh2*^fl/fl^) with TCR-transgenic SMARTA mice that expressed the congenic marker CD45.1 to generate *Ezh2*^fl/fl^ SMARTA mice (hereafter designated *Ezh2*^fl/fl^ SM). Subsequently, we used a retroviral transduction system to overexpress codon-improved Cre recombinase (iCre) for the conditional deletion of EZH2 in virus-specific *Ezh2*^fl/fl^ SM cells. Then, we transferred retrovirus-transduced and nontransduced *Ezh2*^fl/fl^ SM cells or WT SM cells (*Ezh2*^+/+^ SM) into naïve WT recipients that expressed the congenic marker CD45.2 and subsequently infected animals with the LCMV Armstrong strain (Fig. 3a). On day 2 after infection, we visualized the efficient deletion of EZH2 expression in *Ezh2*^fl/fl^ SM cells overexpressing iCre (Supplementary Figure S3a). Notably, only 4.7% of iCre-expressing *Ezh2*^fl/fl^ SM cells differentiated into CD25^lo^CXCR5^+^ T_FH_ cells, whereas ~20% of virus-specific *Ezh2*^+/+^ SM cells (with and without iCre recombinase) and *Ezh2*^fl/fl^ SM cells (without iCre recombinase) were committed to the T_FH_ cell fate (Fig. 3b, c), highlighting the importance of EZH2 in T_FH_ fate determination.

We bred *Ezh2*^fl/fl^ mice with SMARTA mice that transgenically expressed Cre recombinase downstream of the *Cd4* enhancer, promoter and silencer sequences (hereafter called *Ezh2*^fl/fl^ SM-Cre) to further confirm the role of EZH2 in the early differentiation of virus-specific T_FH_ cells. We next mixed equal numbers of *Ezh2*^+/+^ SM-Cre cells with *Ezh2*^fl/fl^ SM-Cre cells and cotransferred these cells into WT recipients, which were subsequently infected with the LCMV Armstrong strain (Fig. 3d). On day 2 after infection, we validated the efficiency of *Ezh2* deletion in *Ezh2*^fl/fl^ SM-Cre cells (Supplementary Figure S3b) and observed a remarkable reduction in the T_FH_ cell commitment of the *Ezh2*^fl/fl^ SM-Cre cells compared to *Ezh2*^+/+^ SM-Cre cells (Fig. 3e, f). Furthermore, we compared the expression levels of molecules that are closely associated with T_FH_ fate commitment, including TCF-1, Bcl-6, ICOS and CXCR5, between T_FH_ cells that differentiated from *Ezh2*^fl/fl^ SM-Cre cells and *Ezh2*^+/+^ SM-Cre cells (cells within the red circle in Fig. 3e). The levels of all examined molecules were substantially reduced in *Ezh2*^fl/fl^ SM-Cre cell-derived T_FH_ cells compared to those in T_FH_ cells that differentiated from *Ezh2*^+/+^ SM-Cre cells (Fig. 3g). Additionally, these phenotypes characterizing the early T_FH_ differentiation of *Ezh2*^fl/fl^ SM-Cre cells in response to LCMV infection were largely confirmed to be the same cells upon an acute intracellular bacterial infection with a recombinant *L. monocytogenes* strain that expressed the LCMV glycoprotein epitope I-A^b^GP61–80 (Supplementary Figure S3e–i).

Next, we investigated whether the inhibition of the EZH2-mediated H3K27me3 modification also led to defects in the T_FH_ cell commitment of virus-specific CD4^+^ T cells in response to a viral infection. For this purpose, we treated SMARTA cells with vehicle or the small molecule EPZ6438, which is a specific inhibitor of the H3K27me3 modification,^60^ in vitro for 3 days and subsequently transferred these cells into WT recipients that were then infected with the LCMV Armstrong strain (Fig. 3h). Treatment with EPZ6438 efficiently reduced the levels of the H3K27me3 modification in SMARTA cells without affecting EZH2 expression (Supplementary Figure S3c and d). Notably, the inhibition of the H3K27me3 modification by the EPZ6438 treatment resulted in a substantial reduction in T_FH_ cell differentiation on day 2 postinfection compared to the T_FH_ commitment of vehicle-treated SMARTA cells (Fig. 3i, j). Furthermore, similar to results obtained using *Ezh2*^fl/fl^ SM-Cre cell-derived T_FH_ cells (Fig. 3g), T_FH_ cells that differentiated from EPZ6438-treated SMARTA cells also exhibited lower expression of T_FH_ lineage-associated molecules, including TCF-1, Bcl-6, ICOS and CXCR5, compared to that of T_FH_ cells derived from vehicle-treated SMARTA cells (Fig. 3k). Taken together, these data validated the hypothesis that EZH2 expression and the subsequent H3K27me3 modification in virus-specific CD4^+^ T cells were important for early commitment to the T_FH_ cell fate in response to an acute viral infection.

### Requirement for EZH2 expression in endogenous virus-specific T_FH_ cell differentiation

**Fig. 4:**
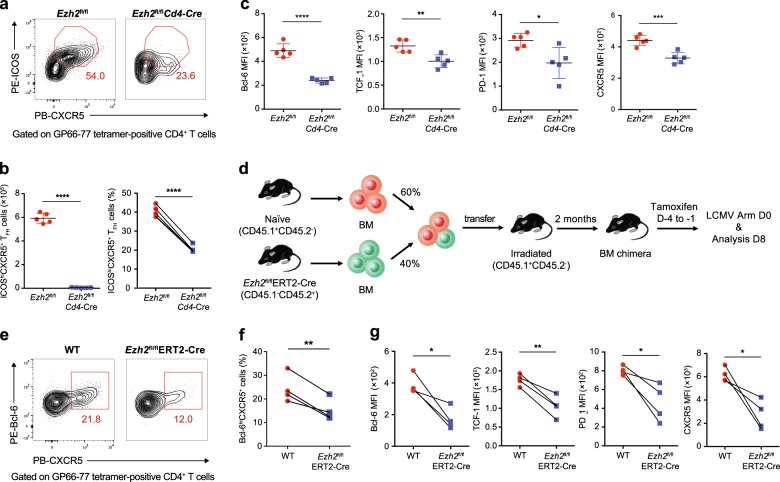
Requirement for EZH2 expression in endogenous virus-specific T_FH_ cell differentiation. **a** Flow cytometry analysis of GP66–77 tetramer-positive CD4^+^ T cells in the spleens of *Ezh2*^fl/fl^*Cd4*-Cre mice and *Ezh2*^fl/fl^ mice on day 8 after LCMV Armstrong infection. The numbers adjacent to the outlined areas indicate the proportions of ICOS^hi^CXCR5^+^ T_FH_ cells. **b** The percentage (left panel) and number (right panel) of ICOS^hi^CXCR5^+^ T_FH_ cells in **a**. **c** Quantification of Bcl-6, TCF-1, PD-1 and CXCR5 levels in the ICOS^hi^CXCR5^+^ T_FH_ cells shown in **a**. **d** Setup of the BM chimera experiment. Irradiated CD45.1^+^ WT recipients underwent the adoptive transfer of CD45.1^+^ WT BM cells (60%) and CD45.2^+^ *Ezh2*^fl/fl^ERT2-Cre BM cells (40%). Two months after reconstitution, the recipients were treated with tamoxifen and then infected with the LCMV Armstrong strain. **e** Flow cytometry analysis of GP66–77 tetramer-positive CD4^+^ T cells in the spleens of recipient mice shown in **d** on day 8 after the LCMV Armstrong infection. The numbers adjacent to the outlined areas indicate the proportions of Bcl-6^hi^CXCR5^+^ T_FH_ cells, which are summarized in **f**. **g** Quantification of Bcl-6, TCF-1, PD-1 and CXCR5 levels in the Bcl-6^hi^CXCR5^+^ T_FH_ cells shown in **e**. NS not significant; *P < 0.05, **P < 0.01, ***P < 0.001 and ****P < 0.0001 (unpaired two-tailed *t*-test (**b** and **c**) or paired two-tailed *t*-test (**f** and **g**)). The data are representative of two independent experiments with at least four mice (**b**, **c**, **f** and **g**) per group (error bars in **b** and **c** indicate the s.d.)

We bred *Ezh2*^fl/fl^ mice with *Cd4*-Cre transgenic mice to further analyze the role of EZH2 in non-TCR transgenic, endogenous virus-specific T_FH_ cell differentiation. The resulting *Ezh2*^fl/fl^*Cd4*-Cre mice conditionally lost EZH2 expression in their CD4^+^ T cell population (Supplementary Figure S4a). On day 8 after infection with the LCMV Armstrong strain, *Ezh2*^fl/fl^*Cd4*-Cre mice exhibited a remarkable reduction in the virus-specific GP66–77 tetramer-positive CXCR5^+^ICOS^+^ T_FH_ cell population in the spleen compared to that in *Ezh2*^fl/fl^ control mice (Fig. 4a, b). Furthermore, the tetramer-positive T_FH_ cells from *Ezh2*^fl/fl^*Cd4*-Cre mice exhibited decreased levels of T_FH_ differentiation-associated molecules, such as Bcl-6, TCF-1, PD-1 and CXCR5, compared to T_FH_ cells from *Ezh2*^fl/fl^ control mice (Fig. 4c). These data revealed the effect of EZH2 deficiency on the T_FH_ differentiation of endogenous virus-specific CD4^+^ T cells, which were highly consistent with our observations in the *Ezh2*-null TCR-transgenic SMARTA cells shown in Fig. 3. Additionally, we compared the T_FH_ differentiation of bulk virus-activated Foxp3^−^CD44^hi^ CD4^+^ T cells from *Ezh2*^fl/fl^*Cd4*-Cre mice and *Ezh2*^fl/fl^ control mice after LCMV infection. Similar to the impaired T_FH_ differentiation of GP66–77 tetramer-positive CD4^+^ T cells in *Ezh2*^fl/fl^*Cd4*-Cre mice upon infection, the bulk-activated Foxp3^−^CD44^hi^CD4^+^ T cells from these mice also exhibited compromised CD44^hi^CXCR5^+^T_FH_ differentiation, as evidenced by the decreases in the proportion of T_FH_ cells, the T_FH_ to T_H_1 ratio and the absolute T_FH_ number compared to that in *Ezh2*^fl/fl^ control mice (Supplementary Figure S4b–d). As expected, T_FH_ cells lacking EZH2 expression displayed reduced levels of an array of T_FH_ lineage-associated proteins, including TCF-1, Bcl-6, PD-1 and ICOS (Supplementary Figure S4e).

Consistent with the defective T_FH_ differentiation caused by *Ezh2* deficiency in CD4^+^ T cells, *Ezh2*^fl/fl^*Cd4*-Cre mice exhibited remarkably reduced frequencies and absolute numbers of PNA^+^FAS^+^GC B cells in the spleen on day 8 postinfection (Supplementary Figure S4f, g). The typical GC structure was also rarely observed in tissue sections of the spleens of *Ezh2*^fl/fl^*Cd4*-Cre mice compared to sections from *Ezh2*^fl/fl^ control mice, as revealed by a confocal microscopy analysis (Supplementary Figure S4h). Consistently, we noted less differentiation of B220^lo^CD138^hi^ plasma cells in the spleens of *Ezh2*^fl/fl^*Cd4*-Cre mice than that in the spleens of *Ezh2*^fl/fl^ control mice (Supplementary Figure S4i and j). Given the defective T_FH_ differentiation and consequent scarcity of the GC B cell population in response to LCMV infection, *Ezh2*^fl/fl^*Cd4*-Cre mice exhibited substantially reduced LCMV-specific IgG titers on days 8 and 90 postinfection compared with those of their *Ezh2*^fl/fl^ counterparts (Supplementary Figure S4k).

We generated BM chimeras by mixing congenitally marked BM cells from *Ezh2*^fl/fl^ERT2-Cre mice (expressing a fusion protein with a tamoxifen-sensitive estrogen receptor variant and Cre; CD45.2, 40%) with BM cells from WT mice (CD45.1, 60%) and subsequently injecting these mixtures into irradiated WT recipients to more precisely evaluate the role of cell-autonomous EZH2 in the mechanism regulating endogenous T_FH_ differentiation (Fig. 4d). After reconstitution, we first administered tamoxifen to efficiently delete EZH2 (Supplementary Figure S5a) and then infected these chimeric mice with the LCMV Armstrong strain. On day 8 after infection, a decreased percentage of Bcl-6^hi^CXCR5^+^ T_FH_ cells was observed among GP66–77 tetramer-positive CD4^+^ T cells originating from *Ezh2*^fl/fl^ ERT2-Cre mice compared with that of cells of WT origin (Fig. 4e, f). Significantly lower levels of TCF-1, Bcl6, PD-1 and CXCR5 were also detected in EZH2-deficient T_FH_ cells (*Ezh2*^fl/fl^ ERT2-Cre) than those in control T_FH_ cells (WT) (Fig. 4g). We observed similar phenotypes in bulk T_FH_ cells from *Ezh2*^fl/fl^ERT2-Cre and WT mice (Supplementary Figure S5b–e). Altogether, these data highlighted the importance of cell-intrinsic EZH2 in the mechanism regulating endogenous T_FH_ differentiation in response to an acute viral infection.

### EZH2 is not required for the late differentiation and maintenance of virus-specific T_FH_ cells during an acute viral infection

**Fig. 5:**
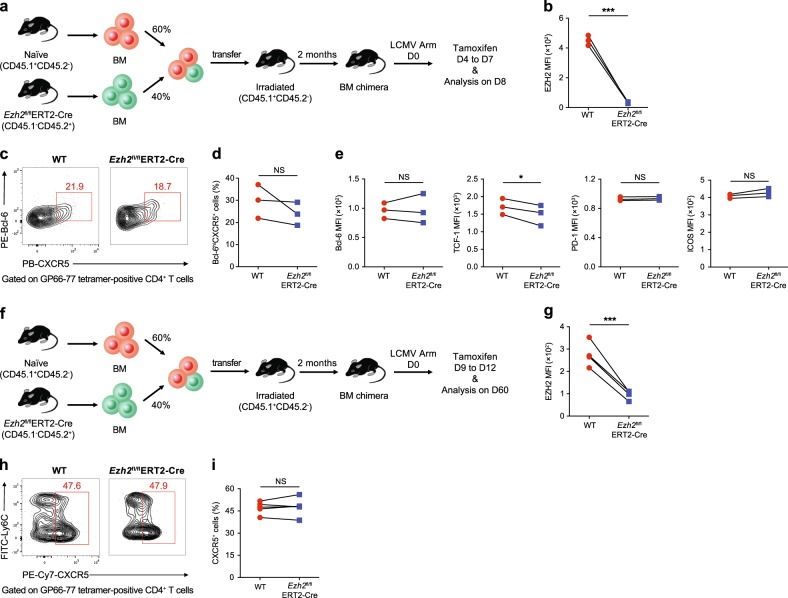
EZH2 is not essential for the late differentiation and maintenance of virus-specific T_FH_ cells during an acute viral infection. **a** Setup of the BM chimera experiment. Irradiated CD45.1^+^ WT recipients underwent adoptive transfer of CD45.1^+^ WT BM cells (60%) and CD45.2^+^ *Ezh2*^fl/fl^ERT2-Cre BM cells (40%). Two months after reconstitution, the recipients were infected with LCMV Armstrong, followed by the administration of tamoxifen from days 4 to 7 after infection and an analysis on day 8. **b** Expression of EZH2 in virus-specific Bcl6^hi^CXCR5^+^ T_FH_ cells from the BM chimeric WT and *Ezh2*^fl/fl^ERT2-Cre mice shown in **a**. **c** Flow cytometry analysis of GP66–77 tetramer-positive CD4^+^ T cells in the spleens of the chimeras described in **a** on day 8 after the LCMV Armstrong infection. Numbers adjacent to outlined areas indicate the percentage of Bcl-6^hi^CXCR5^+^ T_FH_ cells, which were summarized in **d**. **e** Levels of Bcl-6, TCF-1, PD-1 and ICOS in the Bcl-6^hi^CXCR5^+^ T_FH_ cells shown in **c**. **f** The BM chimeras described in **a** were infected with LCMV Armstrong, treated with tamoxifen on days 9 to 12 after infection and analyzed on day 60. **g** Quantification of EZH2 expression in virus-specific CXCR5^+^ T_FH_ cells originating from BM chimeric WT and *Ezh2*^fl/fl^ERT2-Cre mice shown in **f**. **h** Flow cytometry of GP66–77 tetramer-positive CD4^+^ T cells in the spleens of the chimeras described in **f** on day 60 after the LCMV Armstrong infection. Numbers adjacent to outlined areas indicate the percentage of CXCR5^+^ T_FH_ cells, which were summarized in **i**. NS not significant; *P < 0.05 and ***P < 0.001 (paired two-tailed *t*-test (**b**, **d**, **e**, **g** and **i**)). The data are representative of two independent experiments with at least three mice (**b**, **d**, **e**, **g**, **h** and **i**) per group

We next determined the role of EZH2 in the late differentiation of T_FH_ cells during an acute infection. BM chimeras were generated by mixing BM cells from *Ezh2*^fl/fl^ERT2-Cre mice (CD45.2, 40%) with BM cells from WT mice (CD45.1, 60%) and subsequently injecting the mixtures into irradiated WT recipients (Fig. 5a). After reconstitution, these chimeric mice were initially infected with the LCMV Armstrong strain and then administered tamoxifen from days 4 to 7 after infection to induce the deletion of EZH2 (Fig. 5b). Notably, we observed comparable virus-specific Bcl-6^hi^CXCR5^+^ T_FH_ cell proportions between cells from *Ezh2*^fl/fl^ ERT2-Cre and WT mice (Fig. 5c, d). Consistent with the intact late T_FH_ cell differentiation, the levels of molecules related to T_FH_ cell differentiation, including Bcl-6, PD-1 and ICOS, were also similar between cells from *Ezh2*^fl/fl^ ERT2-Cre and WT mice (with the exception of a reduction in TCF-1 expression) (Fig. 5e). BM chimeric mice were infected with LCMV Armstrong and then administered tamoxifen from days 9 to 12 to ablate EZH2 expression and further determine whether EZH2 is required for the maintenance of virus-specific T_FH_ cells (Fig. 5f). On day 60 postinfection, normal virus-specific T_FH_ cell proportions were observed in the absence of EZH2 (Fig. 5g–i). Hence, EZH2 is not required for the late differentiation and the maintenance of virus-specific T_FH_ cells in response to an acute viral infection, suggesting a specific effect on early T_FH_ fate commitment.

### EZH2 remodels T_FH_ lineage-associated chromatin accessibility during viral infection

**Fig. 6:**
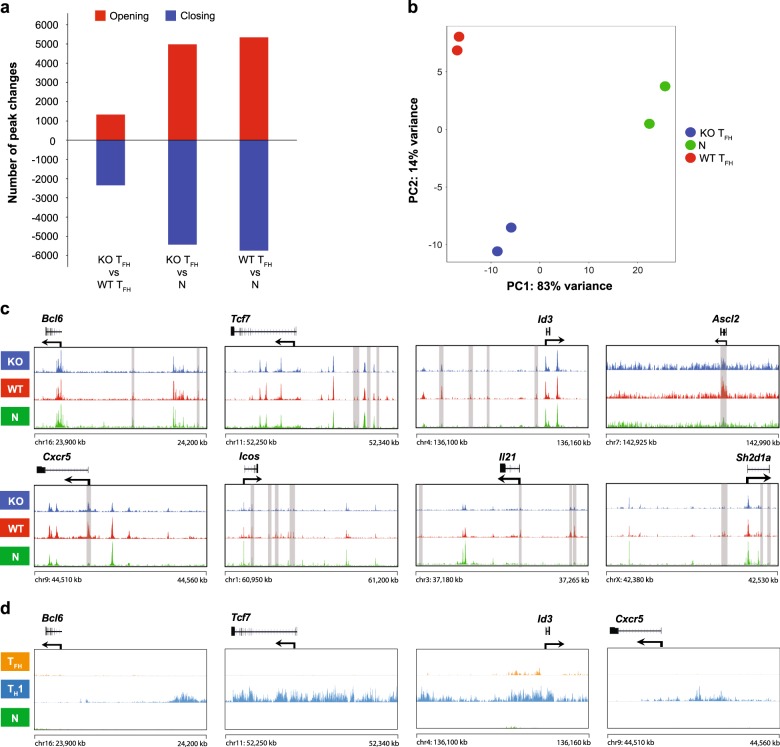
Role of EZH2 in the remodeling of T_FH_ lineage-associated chromatin accessibility during viral infection. **a** Numbers of chromatin peaks with differential accessibility (FDR < 0.05; FC > 1.5) in naïve CD4^+^ T cells (N), WT T_FH_ cells and KO T_FH_ cells. **b** PCA plot of the peak accessibilities in naïve CD4^+^ T cells (green), WT T_FH_ cells (red) and KO T_FH_ cells (blue). Each dot represents a replicate of the group. **c** ATAC-Seq signal profiles of T_FH_ lineage-associated gene loci in naïve CD4^+^ T cells (N), WT T_FH_ cells and KO T_FH_ cells. **d** Tracks of H3K27me3 ChIP-Seq data from T_FH_ lineage-associated gene loci in naïve CD4^+^ T cells (N), T_FH_ cells and T_H_1 cells. The data were obtained from two independent experiments with one biological replicate (pooled from at least five mice per group) in each experiment (**a**–**c**) and from one experiment with one biological replicate (**d**)

We investigated the impact of the EZH2 deficiency on chromatin state changes in virus-specific T_FH_ cells to obtain an understanding of the epigenetic modifications mediated by EZH2 to regulate T_FH_ differentiation. Therefore, we analyzed ATAC-Seq libraries (Supplementary Figure S6a, b) generated from WT T_FH_ cells, EZH2-KO T_FH_ cells and naïve CD4^+^ T cells. Compared to naïve CD4^+^ cells, both WT T_FH_ and KO T_FH_ cells showed significant changes in opening and closing peaks in the genome, suggesting the presence of different chromatin states (Fig. 6a). This finding was clearly revealed in the PCA plot (Fig. 6b): the KO T_FH_ cells showed a chromatin state that differed from WT T_FH_ cells and naïve T cells. The EZH2 deficiency resulted in the remodeling of a cluster of specific gene loci that are closely associated with T_FH_ differentiation, which were almost identical to the loci identified in Fig. 1d, including *Bcl6*, *Tcf7*, *Id3*, *Ascl2*, *Cxcr5*, *Icos*, *Il21* and *Sh2d1a*^1^ (Fig. 6c). Notably, these loci were less open in *Ezh2* KO T_FH_ cells than those in WT T_FH_ cells (Fig. 6c). Because the EZH2-directed H3K27me3 modification is generally associated with a closed state of chromatin accessibility, we further probed whether these loci were marked by the H2K27me3 modification. Therefore, we sorted virus-specific T_FH_ cells and T_H_1 cells from the SMARTA chimeric mice on day 2 after the LCMV Armstrong infection and naïve CD4^+^ T cells (CD4^+^CD25^−^CD62L^+^CD44^−^) from naïve mice and subsequently performed H3K27me3 ChIP-Seq experiments. Strikingly, T_FH_-associated genes (e.g., *Bcl6*, *Tcf7*, *Id3* and *Cxcr5*) were selectively marked by the EZH2-associated H3K27me3 modification in T_H_1 cells, but not in T_FH_ cells, during the bifurcated CD4^+^ T cell differentiation (Fig. 6d). Thus, although EZH2 functions as a chromatin repressor, it appeared to rewind the chromatin states of certain T_FH_-associated genes to favor T_FH_ differentiation.

### Bcl-6 overexpression rescues compromised H3K27me3 modification-induced T_FH_ cell differentiation

**Fig. 7:**
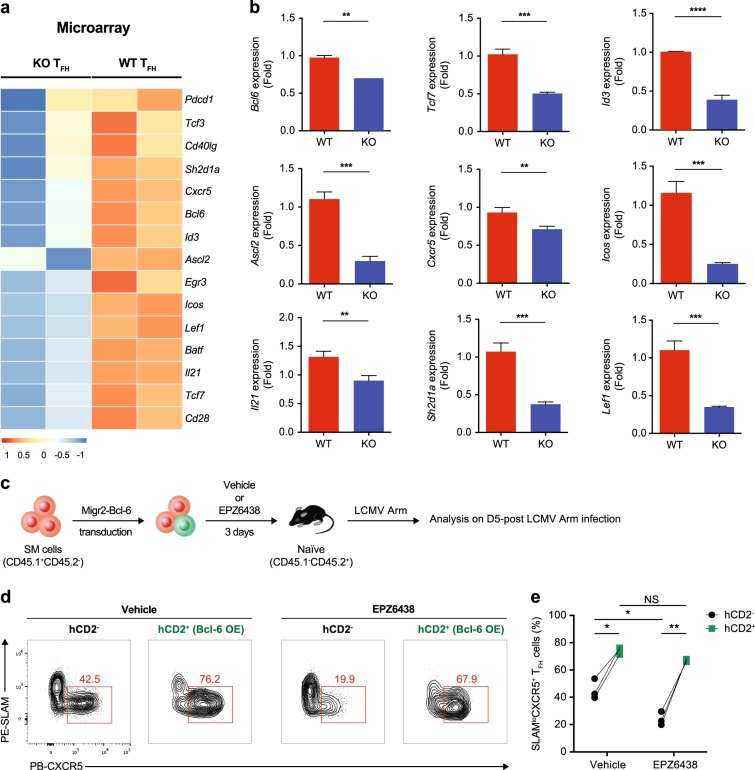
Bcl-6 overexpression rescues compromised H3K27me3 modification-induced T_FH_ cell differentiation. **a** Heat map of the canonical T_FH_ lineage-associated genes in WT T_FH_ cells and EZH2 KO T_FH_ cells based on data obtained from the microarray analysis. **b** Quantitative RT-PCR of selected genes in **a** (normalized to their expression in WT T_FH_ cells). **c** Experimental setup. After treatment with either EPZ6438 or vehicle for 3 days, CD45.1^+^SMARTA (SM) cells transduced with an empty retrovirus or retroviral vector overexpressing *Bcl6* were transferred into WT CD45.2^+^ recipients that were subsequently infected with LCMV Armstrong. The adoptively transferred SM cells were analyzed on day 5 postinfection. **d** Flow cytometry analysis of vehicle/EPZ6438-treated SM cells that were transduced with (hCD2^+^) or without (hCD2^−^) a retrovirus expressing Bcl-6. The numbers adjacent to the outlined areas indicate the percentages of SLAM^lo^CXCR5^+^ T_FH_ cells, which are summarized in **e**. NS not significant; *P < 0.05, **P < 0.01, ***P < 0.001 and ****P < 0.0001 (unpaired two-tailed *t*-test (**b**); paired two-tailed *t*-test (**e**)). The data were obtained from one experiment with two biological replicates pooled from at least ten mice per group (**a**), are representative of two independent experiments with three technical replicates pooled from at least ten mice per group (**b**) or are representative of two independent experiments with at least three mice (**e**) per group (**b**; error bars indicate the s.d.)

Consistent with the reduced accessibility of important ChARs in T_FH_ cells caused by the EZH2 deficiency (Fig. 6c), the expression of the corresponding genes was substantially reduced, as revealed by microarray analyses (Fig. 7a). In addition to the genes analyzed in Fig. 6c (including *Bcl6*, *Tcf7*, *Id3*, *Ascl2*, *Cxcr5*, *Icos*, *Il21* and *Sh2d1a*), we also observed the downregulation of the mRNA levels of several other T_FH_ differentiation-associated genes, such as *Tcf3*,^57^ *Cd40lg*,^7^ *Pdcd1*, *Egr3*,^61^ *Lef1*, *Batf* and *Cd28* (ref. ^62^) (Fig. 7a). We further confirmed our discoveries by performing reverse-transcription quantitative PCR (RT-PCR) assays (Fig. 7b). These results raised the possibility that EZH2-mediated H3K27 trimethylation increases the chromatin accessibility, primarily at T_FH_-differentiation-associated genes, which then induce a favorable transcription program directing T_FH_ lineage commitment.

Bcl-6 is the master regulator of T_FH_ cell differentiation.^16–18^ Because virus-specific CD4^+^ T cells with abundant Bcl-6 and EZH2 expression tend to differentiate into the T_FH_ cell fate (Fig. 2f–h), and lower levels of the Bcl-6 protein (Figs. 3g, k, 4c and g) and mRNA (Fig. 7a, b) and reduced chromatin accessibility (Fig. 6c) are observed in EZH2-deficient T_FH_ cells, we hypothesized that the impaired T_FH_ cell commitment observed in the absence of EZH2 is potentially driven by compromised Bcl-6 expression and that Bcl6 overexpression might rescue the defective T_FH_ differentiation of activated virus-specific CD4^+^ T cells with decreased EZH2 activities. Naïve SMARTA cells were transduced with a retrovirus encoding Bcl-6 and then treated with vehicle or the H3K27me3 inhibitor EPZ6438 in vitro for 3 days to evaluate this hypothesis. After treatment, these cells were adoptively transferred into WT recipients, which were immediately infected with the LCMV Armstrong strain (Fig. 7c). Consistent with published studies,^16–18^ forced Bcl-6 expression biased vehicle-treated SMARTA cells to T_FH_ cell differentiation on day 5 postinfection (Fig. 7d, e; 42.5% vs. 76.2%). Meanwhile, a substantial reduction in the T_FH_ cell differentiation of nontransduced SMARTA cells was observed in the EPZ6438-treated group compared to that in the vehicle-treated group (Fig. 7d, e; 42.5% vs. 19.9%), consistent with our aforementioned data (Fig. 3j). Strikingly, Bcl-6 overexpression in EPZ6438-treated SMARTA cells restored the T_FH_ cell commitment to a similar level as the forced Bcl-6 expression-amplified T_FH_ cell commitment in the vehicle group (Fig. 7d, e; 76.2% vs. 67.9%). Taken together, the EZH2-associated H3K27me3 modification is a crucial positive determinant of Bcl-6 expression, which further directs T_FH_ cell differentiation.

## Discussion

During the initial phase of an acute viral infection, activated virus-specific CD4^+^ T cells differentiate into either T_H_1 or T_FH_ cells.^15,55,56^ Bcl-6 is required to specify the T_FH_ fate choice,^16–18^ and Blimp-1 antagonizes this effect by suppressing Bcl-6 expression and Bcl-6-mediated transcriptional activity.^16^ Thus, the Bcl-6–Blimp-1 balance coordinates the early T_FH_ commitment. The preserved and elevated TCF-1 expression in activated virus-specific CD4^+^ T cells induces Bcl-6 expression but inhibits Blimp-1 expression, and thus, it plays a critical role in guiding the T_FH_ commitment pathway.^15^ Currently, little is known about the epigenetic mechanism that stabilizes these TF networks during early T_FH_ fate determination. In this study, we defined a distinct chromatin state in virus-specific T_FH_ cells compared to virus-specific T_H_1 cells in response to an acute infection. Importantly, early fate-committed T_FH_ cells exhibited higher expression of the histone methyltransferase EZH2 in response to an acute viral infection and accordingly increased levels of the H3K27me3 modification compared with that of their T_H_1 counterparts. The ablation of EZH2 by genetic deletion in virus-specific CD4^+^ T cells substantially reduced the early T_FH_ commitment by disrupting the T_FH_-specific chromatin state. Specifically, EZH2 is required for the stabilization of chromatin accessibility and transcription pattern of *Bcl6*, which is essential for the T_FH_ fate commitment. Thus, our results highlighted the importance of EZH2, a widely recognized chromatin repressor, in the priming of T_FH_ cell fate commitment by reinforcing Bcl-6 expression in activated T_FH_ precursors.

T_FH_ differentiation is characterized as a stepwise process:^1^ early fate commitment (2 days after an acute infection, occurring at the T–B border), maturation (3–4 days after an acute infection, occurring in B-cell follicle and pre-GC) and memory formation (~1–2 months after an acute infection). In our study, the chromatin identity of virus-specific T_FH_ cells was discerned from virus-specific T_H_1 cells within the first 2 days after viral infection. In particular, chromatin remodeling of a cluster of T_FH_ lineage differentiation-associated genes, including *Bcl6*, *Tcf7*, *Id3*, *Ascl2*, *Cxcr5*, *Icos*, *Il21* and *Sh2d1a*, was observed in virus-specific T_FH_ cells. This distinct chromatin pattern is maintained throughout the differentiation process and maximized on day 8 postinfection, indicating a progressive mechanism regulating the chromatin states of T_FH_ cells in response to an acute infection.

TCR stimulation is crucial for the effective induction of EZH2 expression in CD4^+^ T cells.^33,63^ The enhanced strength and duration of the interaction between TCR and pMHC favors T_FH_ differentiation,^64^ which may partially explain the elevated EZH2 expression levels observed in T_FH_ cells compared to those of their T_H_1 counterparts following an acute viral infection. During T_FH_ differentiation, EZH2 expression spikes within 2 days after infection and then decreases to the baseline level that is comparable to naive CD4^+^ T cells. This dynamic pattern of EZH2 expression is further evidenced by a crucial role for EZH2 in the early commitment but not late differentiation or memory maintenance of T_FH_ cells. Strikingly, the ablation of EZH2 in early committed T_FH_ cells leads to the expression of a cluster of T_FH_ lineage differentiation-associated genes with less chromatin accessibility, including *Bcl6*, *Tcf7*, *Id3*, *Ascl2*, *Cxcr5*, *Icos*, *Il21* and *Sh2d1a*. In this regard, TCR-trigged EZH2 seems to imprint a T_FH_-associated chromatin pattern in T_FH_ precursors at an early stage of the bifurcated T_FH_ and T_H_1 differentiation. The EZH2-mediated H3K27me3 modification generally leads to a closed state of chromatin accessibility, but our results identified an essential role for this epigenetic modification in promoting the chromatin accessibility of a cluster of T_FH_-associated genes in T_FH_ cells. These effects appeared to be indirect, since we did not observe the deposition of H3K27me3 marks at these T_FH_-associated loci in T_FH_ cells. Alternatively, EZH2 and its associated H3K27me3 modification may orchestrate other chromatin modifications that directly target specific loci of these genes to promote T_FH_ differentiation. Further studies will be required to identify potential candidate chromatin modifiers downstream of EZH2 and to dissect the underlying regulatory mechanisms.

EZH2 has been reported to negatively regulate the differentiation of an array of effector T_H_ cell subsets, including T_H_1, T_H_2 and T_H_17 cells.^36–38^ These suppressive effects are likely mediated by the EZH2-driven H3K27me3 modifications that directly target and silence a variety of genes required for the differentiation of these cell subsets, including lineage-specifying TFs and/or cytokines, such as *Tbx21*, *Gata3* and *Ifng*, *Il13* and *Il17*. In sharp contrast to its negative role in modulating the differentiation of other T_H_ lineages, our study revealed that EZH2 instructed the early commitment to T_FH_ cell differentiation by stabilizing a cluster of T_FH_ cell lineage-associated genes, including *Bcl6*. Consistent with our results, a newly published study^65^ revealed a requirement for EZH2 in governing T_FH_ cell differentiation by integrating phosphorylation-dependent *Bcl6* activation and H3K27me3-dependent repression of p19Arf. Therefore, EZH2 regulates T_H_ differentiation in a cell-type-specific manner.

In conclusion, the present study is the first to define a distinct chromatin state in early committed T_FH_ cells that is maintained throughout the acute viral infection. The histone methyltransferase EZH2 is involved in this process and plays a critical role in priming the early T_FH_ cell fate commitment by promoting the stabilization of T_FH_ lineage-specific chromatin pattern. These findings provide valuable insights into strategies targeting EZH2 to improve vaccine efficacy and may aid in the development of novel therapeutic strategies for diseases associated with aberrant T_FH_ differentiation.

Accession codes: GEO: ATAC-Seq data and H3K27me3 ChIP-Seq data, GSE110722; microarray data, GSE110458.

## Supplementary information

Supplementary figures

Supplementary figure legends

Supplementary table 1

Supplementary table 2

Supplementary table 3

Supplementary table 4
