# Supplementary material for: The histone methyltransferase EZH2 primes the early differentiation of follicular helper T cells during acute viral infection
Source: Cell Mol Immunol. 2019 Mar 6;17(3):247–60. doi: 10.1038/s41423-019-0219-z (PMC7052164; doi:10.1038/s41423-019-0219-z)

Figure S1

a **Sortig strategy of SM T_{FH} /T_H1 cells on day 2-post LCMV Armstrong infection**

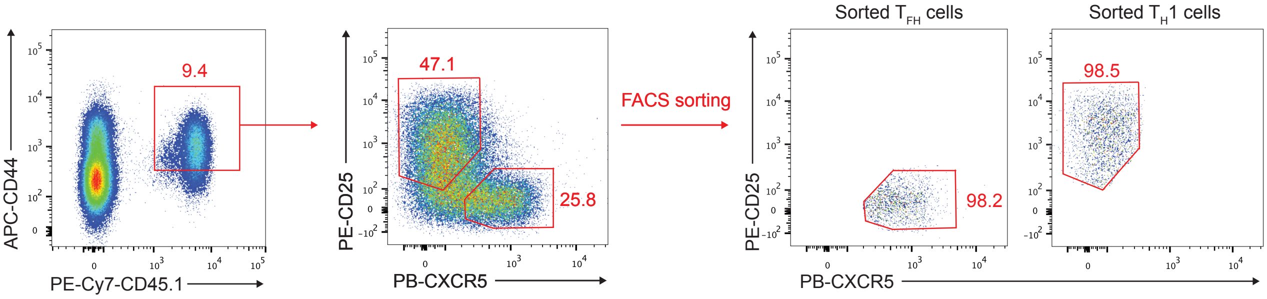

b **Sortig strategy of SM T_{FH} /T_H1 cells on day 5-post LCMV Armstrong infection**

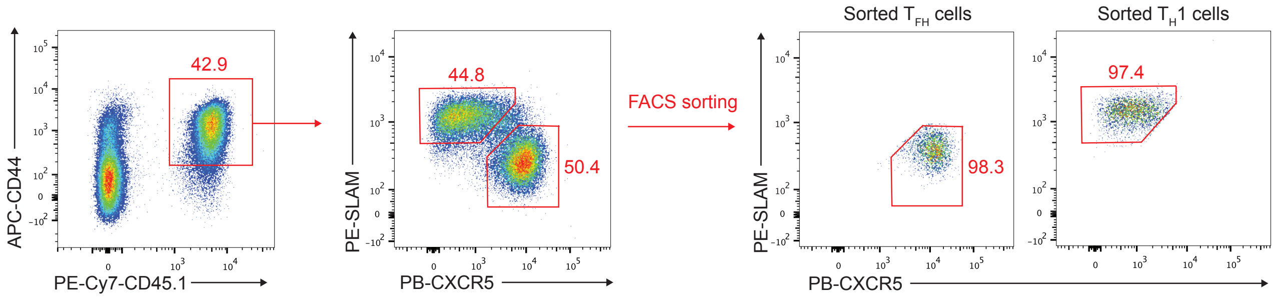

c **Sortig strategy of SM T_{FH} /T_H1 cells on day 8-post LCMV Armstrong infection**

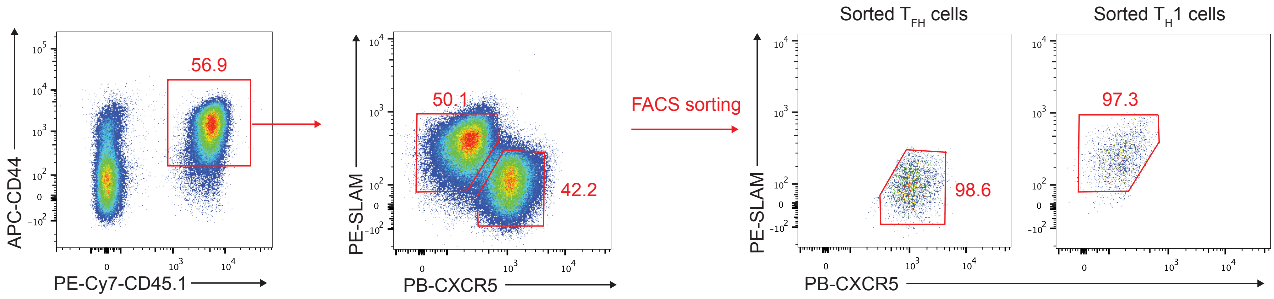

d **Sortig strategy of naïve SM $CD4^+$ T cells**

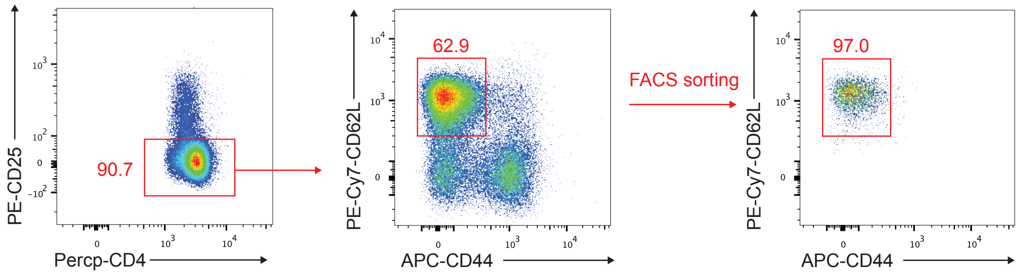

Figure S2

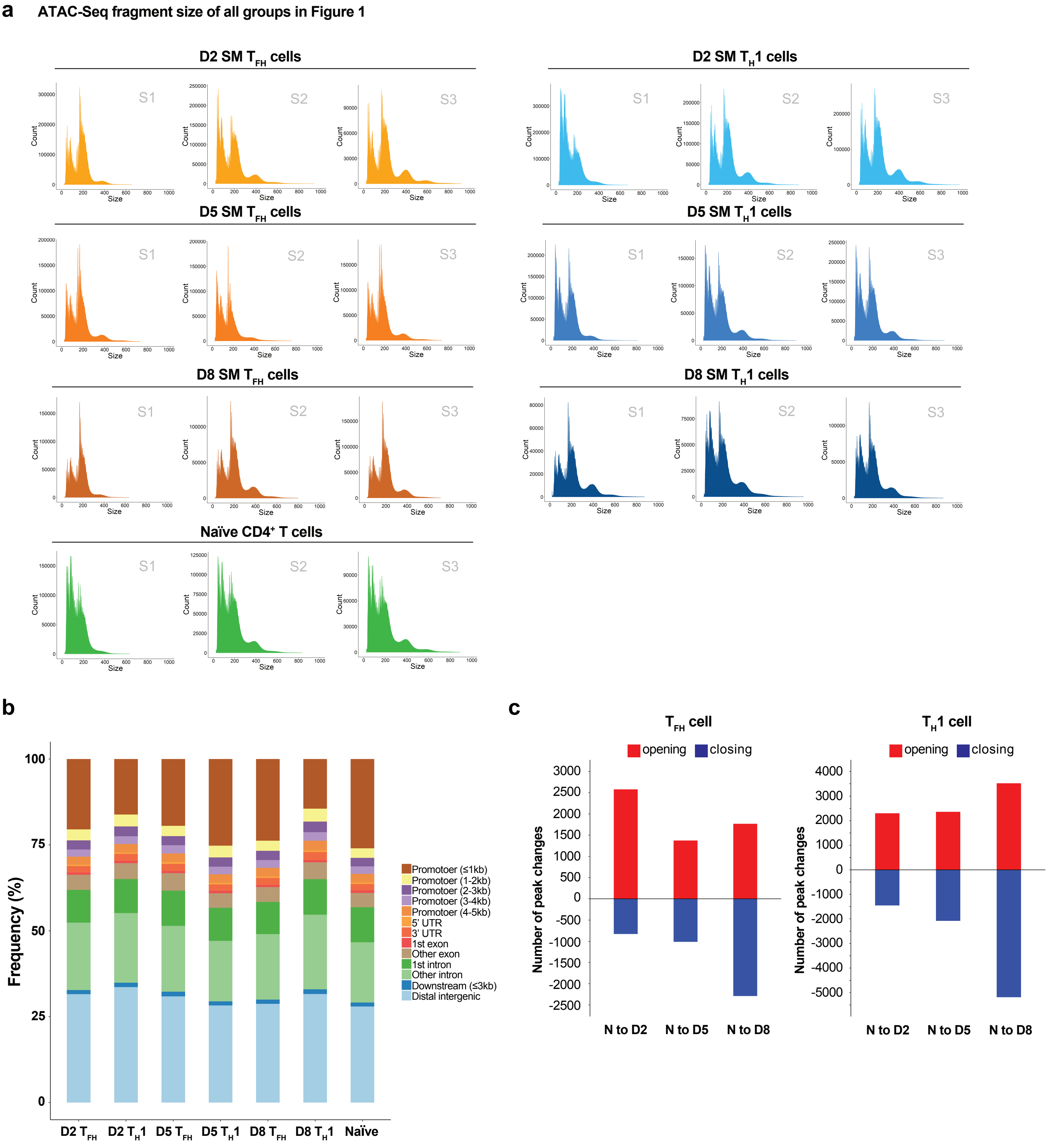
a
ATAC-Seq fragment size of all groups in Figure 1
D2 SM TFH cells
D2 SM TH1 cells
D5 SM TFH cells
D5 SM TH1 cells
D8 SM TFH cells
D8 SM TH1 cells
Naïve CD4+ T cells
S1
S2
S3
Count
Size
b
Frequency (%)
Promotoer (≤1kb)
Promotoer (1-2kb)
Promotoer (2-3kb)
Promotoer (3-4kb)
Promotoer (4-5kb)
5' UTR
3' UTR
1st exon
Other exon
1st intron
Other intron
Downstream (≤3kb)
Distal intergenic
D2 TFH
D2 TH1
D5 TFH
D5 TH1
D8 TFH
D8 TH1
Naïve
c
TFH cell
TH1 cell
opening
closing
Number of peak changes
N to D2
N to D5
N to D8

Figure S3

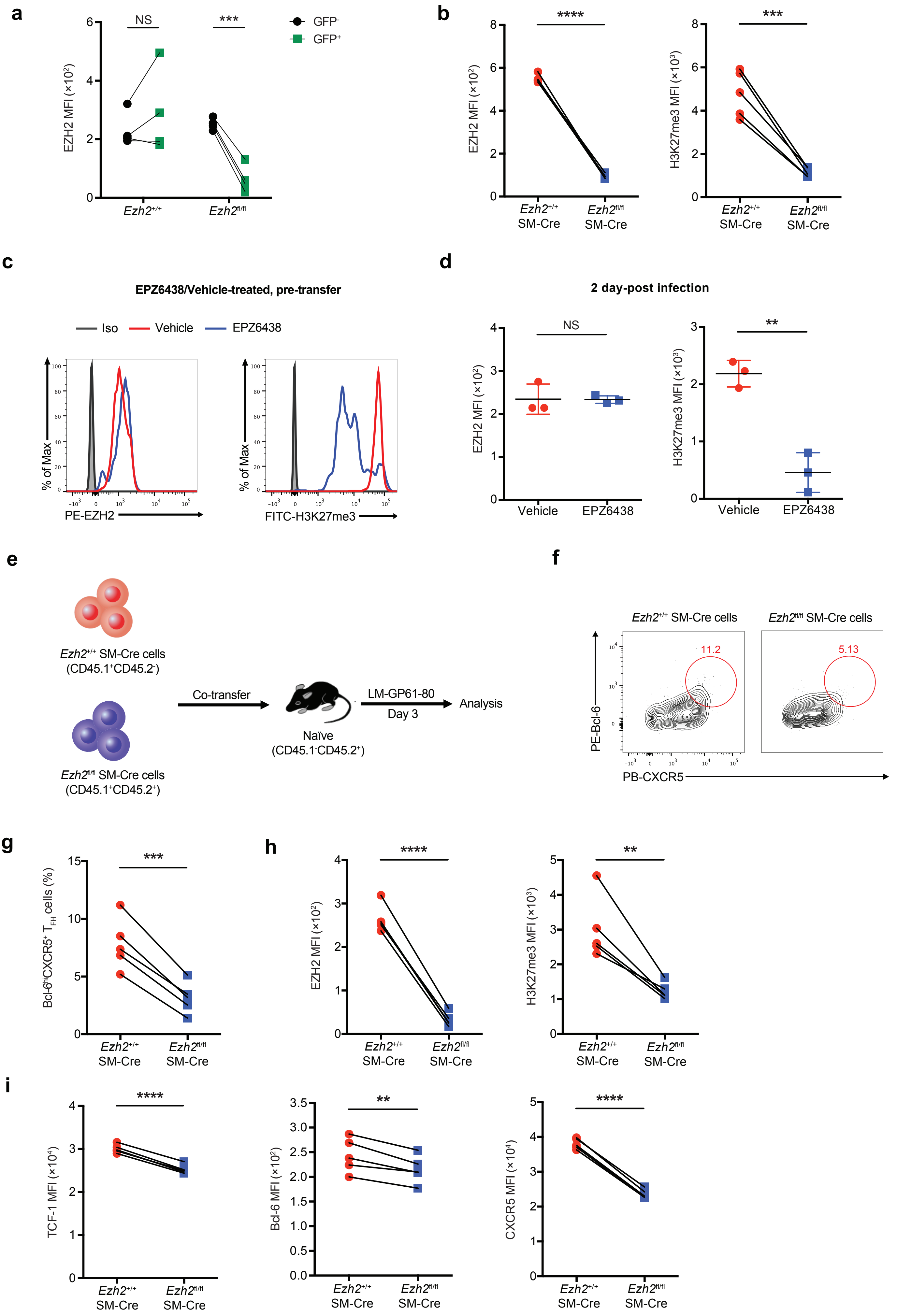

a
EZH2 MFI (×10²)
NS
***
GFP⁻
GFP⁺
Ezh2+/+
Ezh2fl/fl
b
EZH2 MFI (×10²)
H3K27me3 MFI (×10³)
****
***
Ezh2+/+ SM-Cre
Ezh2fl/fl SM-Cre
c
EPZ6438/Vehicle-treated, pre-transfer
Iso
Vehicle
EPZ6438
% of Max
PE-EZH2
FITC-H3K27me3
d
2 day-post infection
EZH2 MFI (×10²)
H3K27me3 MFI (×10³)
NS
**
Vehicle
EPZ6438
e
Ezh2+/+ SM-Cre cells (CD45.1⁺CD45.2⁻)
Ezh2fl/fl SM-Cre cells (CD45.1⁺CD45.2⁺)
Co-transfer
Naïve (CD45.1⁻CD45.2⁺)
LM-GP61-80
Day 3
Analysis
f
Ezh2+/+ SM-Cre cells
Ezh2fl/fl SM-Cre cells
11.2
5.13
PE-Bcl-6
PB-CXCR5
g
Bcl-6hiCXCR5⁺ TFH cells (%)
***
h
EZH2 MFI (×10²)
H3K27me3 MFI (×10³)
****
**
i
TCF-1 MFI (×10⁴)
Bcl-6 MFI (×10²)
CXCR5 MFI (×10⁴)
****
**
****
Ezh2+/+ SM-Cre
Ezh2fl/fl SM-Cre

Figure S4

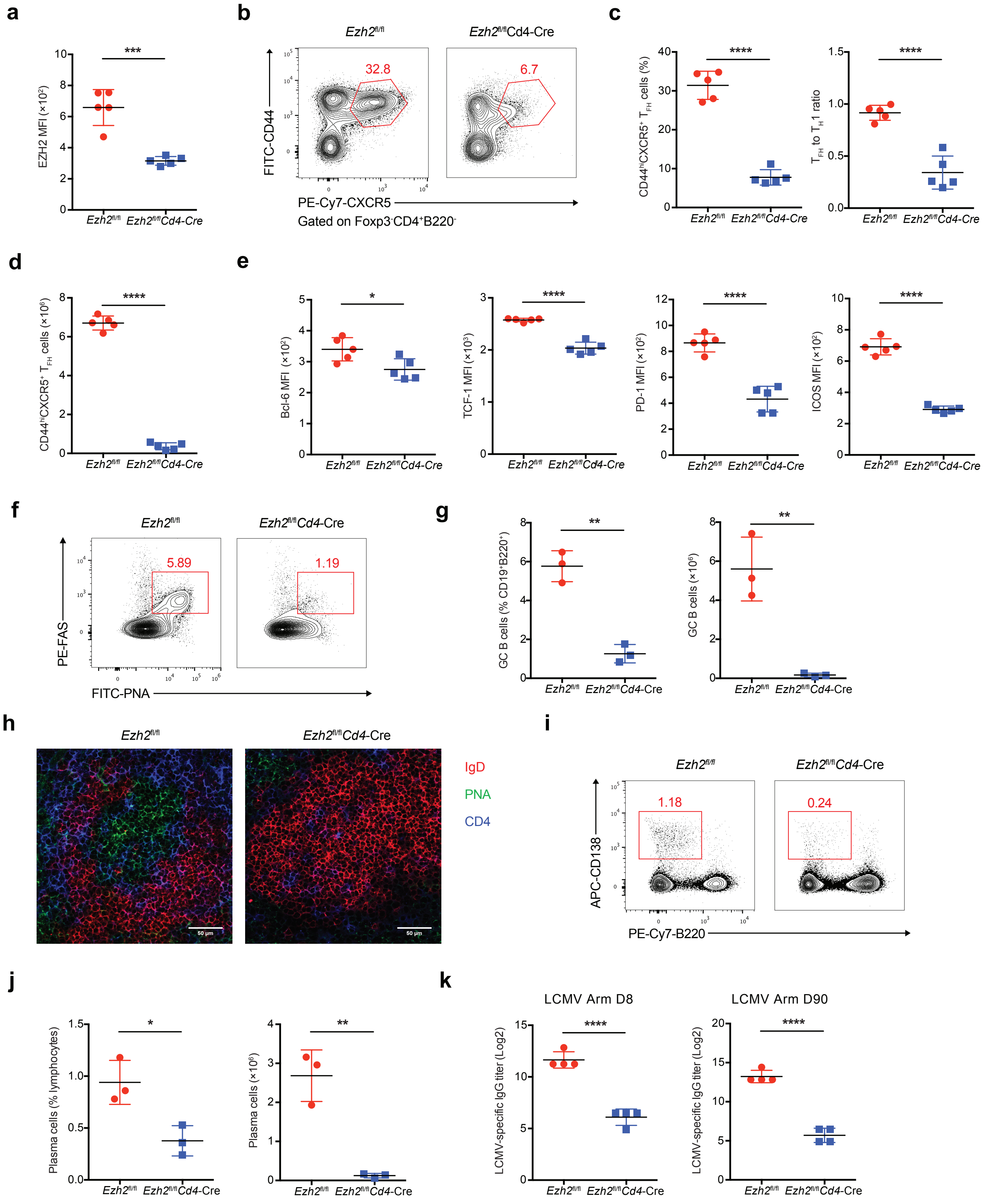
a
EZH2 MFI (×10²)
***
Ezh2fl/fl
Ezh2fl/flCd4-Cre
b
Ezh2fl/fl
Ezh2fl/flCd4-Cre
FITC-CD44
32.8
6.7
PE-Cy7-CXCR5
Gated on Foxp3⁻CD4⁺B220⁻
c
CD44hiCXCR5⁺ TFH cells (%)
TFH to TH1 ratio
****
d
CD44hiCXCR5⁺ TFH cells (×10⁶)
e
Bcl-6 MFI (×10²)
TCF-1 MFI (×10³)
PD-1 MFI (×10²)
ICOS MFI (×10²)
*
f
PE-FAS
FITC-PNA
5.89
1.19
g
GC B cells (% CD19⁺B220⁺)
GC B cells (×10⁶)
**
h
IgD
PNA
CD4
50 μm
i
APC-CD138
PE-Cy7-B220
1.18
0.24
j
Plasma cells (% lymphocytes)
Plasma cells (×10⁶)
k
LCMV Arm D8
LCMV Arm D90
LCMV-specific IgG titer (Log2)

Figure S5

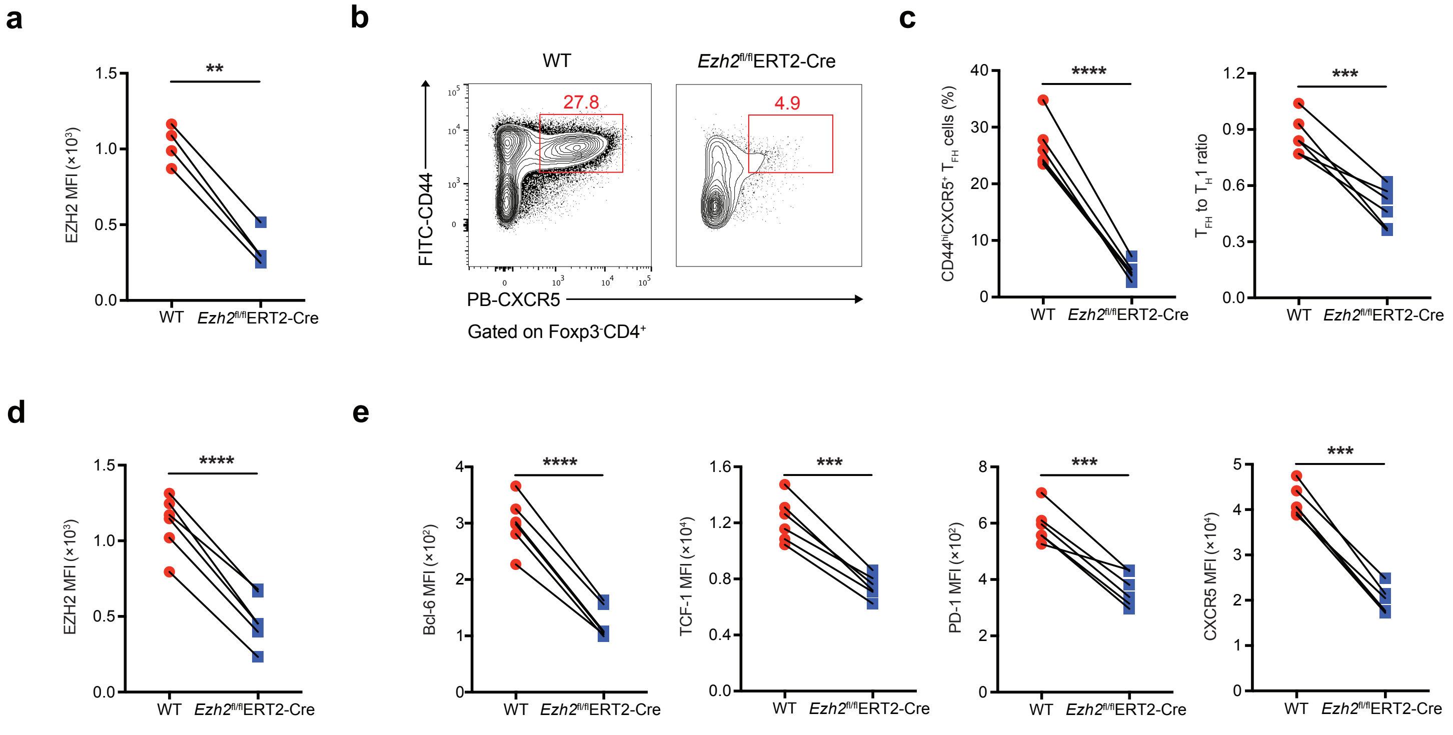
a
b
c
d
e
**
****
***
EZH2 MFI (×10³)
WT
Ezh2fl/flERT2-Cre
FITC-CD44
PB-CXCR5
Gated on Foxp3⁻CD4⁺
27.8
4.9
CD44hiCXCR5⁺ TFH cells (%)
TFH to TH1 ratio
Bcl-6 MFI (×10²)
TCF-1 MFI (×10⁴)
PD-1 MFI (×10²)
CXCR5 MFI (×10⁴)

Figure S6

a ATAC-Seq fragment size of all groups in Figure 6

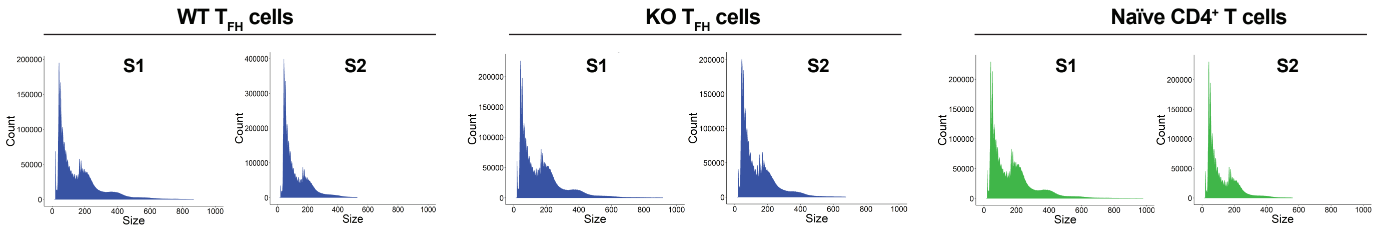

b

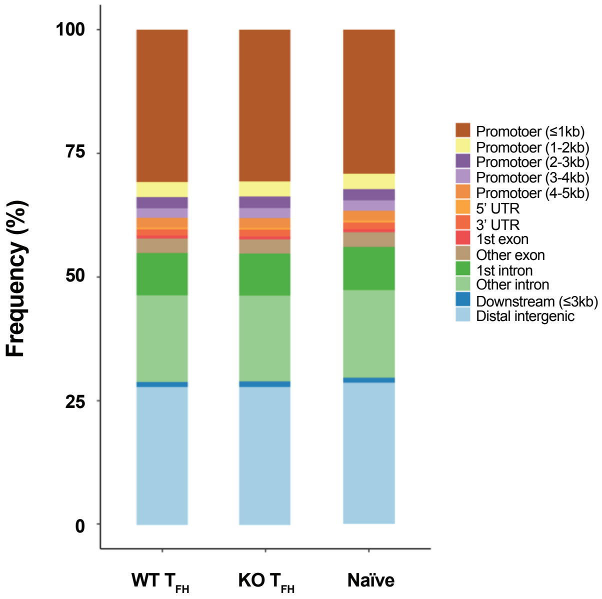

Supplement: Supplementary file 1 — Supplementary figures [file 41423_2019_219_MOESM1_ESM.pdf]
