# Supplementary material for: The histone methyltransferase EZH2 primes the early differentiation of follicular helper T cells during acute viral infection
Source: Cell Mol Immunol. 2019 Mar 6;17(3):247–60. doi: 10.1038/s41423-019-0219-z (PMC7052164; doi:10.1038/s41423-019-0219-z)
Supplement: Supplementary file 2 — Supplementary figure legends [file 41423_2019_219_MOESM2_ESM.docx]

**Supplementary Figure Legends**

**Figure S1. FACS sorting strategies of naïve CD4^+^ T cells, T_FH_ cells and T_H_1 cells in the ATAC-Seq profiles.** SMARTA (SM) T_FH_ cells or SM T_H_1 cells were sorted from the spleens of CD45.2^+^ recipients that underwent adoptive transfer of CD45.1^+^ SM cells on day 2 (**a**), day 5 (**b**) or day 8 (**c**) after LCMV Armstrong infection. (**d**) Naïve CD4^+^ T cells (CD4^+^CD25^-^CD62L^+^CD44^lo^) were sorted from naïve SM mice.

**Figure S2. Quality control of ATAC-Seq profiles of naïve CD4^+^ T cells, T_FH_ cells and T_H_1 cells.** (**a**) ATAC-Seq fragment sizes from all groups in Fig. 1. (**b**) Frequencies of chromatin-accessible regions in naïve CD4^+^ T cells (Naïve), SMARTA (SM) T_FH_ cells and SM T_H_1 cells partitioned into overlapping promoter regions (≤ 1 kb, 1-2 kb, 2-3 kb, 3-4 kb and 4-5 kb), 5’ untranslated regions (UTRs), 3’ UTRs, 1st exons, other exons, 1st introns, and other introns, and downstream (≤ 3 kb) and distal intergenic areas as indicated. (**c**) Numbers of chromatin peaks with differential accessibility (FDR < 0.05; FC > 1.5) between naïve CD4^+^ T cells (N) and SM T_FH_ cells (left) or SM T_H_1 cells (right) at indicated time-post infection.

**Figure S3. EZH2 is essential for early commitment to T_FH_ differentiation.** (**a**) Expression of EZH2 in *Ezh2*^+/+^ SMARTA (SM) cells and *Ezh2*^fl/fl^ SM cells that were transduced with a retrovirus expressing iCre (GFP^+^) or not transduced (GFP^-^) on day 2 after infection of the host with LCMV Armstrong. (**b**) EZH2 and H3K27me3 expression levels in CD25^lo^CXCR5^+^ SMARTA T_FH_ cells from the *Ezh2*^+/+^*Cd4*-Cre or *Ezh2*^fl/fl^*Cd4*-Cre lines as described in Fig. 3e. (**c**) Quantitation of EZH2 and H3K27me3 expression levels in SM cells treated for 3 days with 2 μM EPZ6438 or vehicle. (**d**) The expression levels of EZH2 and H3K27me3 in the CD25^lo^CXCR5^+^ T_FH_ cells described in Fig. 3j. (**e**) Experimental setup. *Ezh2*^+/+^*Cd4*-Cre SM cells (*Ezh2*^+/+^ SM-Cre; CD45.1^+^CD45.2^-^) and *Ezh2*^fl/fl^*Cd4*-Cre SM cells (*Ezh2*^fl/fl^ SM-Cre; CD45.1^+^CD45.2^+^) were co-transferred into WT recipients (CD45.1^-^CD45.2^+^) that were assessed on day 3 after infection with *Listeria monocytogenes* expressing GP61-80 (LM-GP61-80). (**f**) Flow cytometry analysis of the *Ezh2*^+/+^ SM-Cre and *Ezh2*^fl/fl^ SM-Cre SM cells in (e) on day 3 post-LM-GP61 infection. The numbers adjacent to the outlined areas indicate the percentages of Bcl-6^hi^CXCR5^+^ T_FH_ cells, which are summarized in (g). (**h**) EZH2 and H3K27me3 expression levels in the *Ezh2*^+/+^ SM-Cre and *Ezh2*^fl/fl^ SM-Cre SM cells described in (e). (**i**) Quantification of the TCF-1, Bcl-6 and CXCR5 expression levels in the *Ezh2*^+/+^ SM-Cre and *Ezh2*^fl/fl^ SM-Cre SM cells described in (e). NS, not significant; **P* < 0.05, ***P* < 0.01, ****P* < 0.001 and *****P* < 0.0001 (paired two-tailed t-test (a, b, g, h, i) or unpaired two-tailed t-test (d)). The data are representative of two independent experiments (a, b, d, g, h, i) with at least three mice per group (d; error bar, s.d.).

**Figure S4. Requirement of EZH2 expression for endogenous bulk T_FH_ cell differentiation and T_FH_ cell effector function.** (**a**) Expression of EZH2 in activated virus-specific bulk CD44^hi^CXCR5^+^ T_FH_ cells from the spleens of *Ezh2*^fl/fl^*Cd4*-Cre or *Ezh2*^fl/fl^ mice on day 8 post-LCMV Armstrong infection. (**b**) Flow cytometry analysis of Foxp3^-^CD4^+^ cells from the spleens described in (a). The numbers adjacent to the outlined areas indicate the proportions of CD44^hi^CXCR5^+^ T_FH_ cells. (**c**) Summary of CD44^hi^CXCR5^+^ T_FH_ cell proportions (left) and the ratios of CD44^hi^CXCR5^+^ T_FH_ cells to CD44^hi^CXCR5^-^ T_H_1 cells (right) in (b). (**d**) Numbers of CD44^hi^CXCR5^+^ T_FH_ cells in (b). (**e**) Quantification of Bcl-6, TCF-1, PD-1 and ICOS in the CD44^hi^CXCR5^+^ T_FH_ cells in (b). (**f**) Flow analysis of B220^+^CD19^+^ B cells from the spleens of *Ezh2*^fl/fl^*Cd4*-Cre or *Ezh2*^fl/fl^ mice on day 8 post-LCMV infection. The numbers adjacent to the outlined areas indicate the proportions of FAS^+^PNA^+^ GC B cells. (**g**) The percentages (left) and numbers (right) of GC B cells in (f). (**h**) Immunofluorescence analysis of the GCs from the spleens of *Ezh2*^fl/fl^*Cd4*-Cre or *Ezh2*^fl/fl^ mice on day 8 post-LCMV infection. Scale bar, 50 μm. (**i**) Flow cytometry analysis of lymphocytes in the spleens of *Ezh2*^fl/fl^*Cd4*-Cre or *Ezh2*^fl/fl^ mice on day 8 post-LCMV infection. The numbers adjacent to the outlined areas indicate the percentages of CD138^hi^B220^lo^ plasma cells. (**j**) The percentages (left) and numbers (right) of plasma cells in (i). (**k**) LCMV-specific IgG in the sera of the *Ezh2*^fl/fl^*Cd4*-cre or *Ezh2*^fl/fl^ mice was measured by ELISA on days 8 (left) and 90 (right) after LCMV Armstrong infection. NS, not significant; **P* < 0.05, ***P* < 0.01, ****P* < 0.001 and *****P* < 0.0001 (unpaired two-tailed t-test (a, c, d, e, g, j, k)). The data are representative of three independent experiments with at least three mice per group (a, c, d, e, g, j, k**)** (error bars (a, c, d, e, g, j, k), s.d.).

**Figure S5. The role of cell-autonomous EZH2 in the regulation of endogenous activated virus-specific bulk T_FH_ differentiation.** (**a**) EZH2 expression levels in bulk CD44^hi^CXCR5^+^ T_FH_ cells of WT and *Ezh2*^fl/fl^ERT2-Cre origin mice on day 8 after the infection of bone marrow chimera (BMC) recipients (Fig. 4d). (**b**) Flow cytometry analysis of CD4^+^Foxp3^-^ cells of the WT and *Ezh2*^fl/fl^ERT2-Cre origin spleens of the BMC mice described in Fig. 4d on day 8 after LCMV Armstrong infection. The numbers adjacent to the outlined areas indicate the percentages of CD44^hi^CXCR5^+^ T_FH_ cells. (**c**) The proportions of CD44^hi^CXCR5^+^ T_FH_ cells (left) and the ratio of CD44^hi^CXCR5^+^ T_FH_ cells to CD44^hi^CXCR5^-^ T_H_1 cells (right) in (b). (**d** and **e**) The expression levels of EZH2 (d), Bcl-6, TCF-1, PD-1 and ICOS (e) in the CD44^hi^CXCR5^+^ T_FH_ cells in (b). ***P* < 0.01, ****P* < 0.001 and *****P* < 0.0001 (paired two-tailed t-test (a, c, d, e)). The data are representative of two independent experiments with at least four mice per group (a, c, d, e).

**Figure S6. Quality control of ATAC-Seq profiles of *Ezh2*-WT and *Ezh2*-KO bulk T_FH_ cells.** (**a**) ATAC-Seq fragment sizes from WT T_FH_ cells or KO T_FH_ cells or naïve CD4^+^ T cells. (**b**) Frequencies of the chromatin-accessible regions of groups in **a** partitioned into overlapping promoter regions (≤ 1 kb, 1-2 kb, 2-3 kb, 3-4 kb and 4-5 kb), 5’ UTRs, 3’ UTRs, 1st exons, other exons, 1st introns, other introns and downstream (≤ 3 kb) and distal intergenic areas as indicated.
