# Supplementary material for: The histone methyltransferase EZH2 primes the early differentiation of follicular helper T cells during acute viral infection
Source: Cell Mol Immunol. 2019 Mar 6;17(3):247–60. doi: 10.1038/s41423-019-0219-z (PMC7052164; doi:10.1038/s41423-019-0219-z)
Supplement: Supplementary file 3 — Supplementary table 1 [file 41423_2019_219_MOESM3_ESM.docx]

**Supplementary Table 1. Differential peaks in cluster 3 in Fig. 1c**

| SYMBOL | GENENAME |
| --- | --- |
| 0610040F04Rik | RIKEN cDNA 0610040F04 gene |
| 1110037F02Rik | RIKEN cDNA 1110037F02 gene |
| 1110059E24Rik | RIKEN cDNA 1110059E24 gene |
| 1190002N15Rik | RIKEN cDNA 1190002N15 gene |
| 1500015L24Rik | RIKEN cDNA 1500015L24 gene |
| 1700011H14Rik | RIKEN cDNA 1700011H14 gene |
| 1700012B07Rik | RIKEN cDNA 1700012B07 gene |
| 1700016G22Rik | RIKEN cDNA 1700016G22 gene |
| 1700016L21Rik | RIKEN cDNA 1700016L21 gene |
| 1700017B05Rik | RIKEN cDNA 1700017B05 gene |
| 1700017D01Rik | RIKEN cDNA 1700017D01 gene |
| 1700018C11Rik | RIKEN cDNA 1700018C11 gene |
| 1700019C18Rik | RIKEN cDNA 1700019C18 gene |
| 1700021F07Rik | RIKEN cDNA 1700021F07 gene |
| 1700028K03Rik | RIKEN cDNA 1700028K03 gene |
| 1700029H14Rik | RIKEN cDNA 1700029H14 gene |
| 1700029J03Rik | RIKEN cDNA 1700029J03 gene |
| 1700029M20Rik | RIKEN cDNA 1700029M20 gene |
| 1700029N11Rik | RIKEN cDNA 1700029N11 gene |
| 1700030O20Rik | RIKEN cDNA 1700030O20 gene |
| 1700040L02Rik | RIKEN cDNA 1700040L02 gene |
| 1700041M19Rik | RIKEN cDNA 1700041M19 gene |
| 1700056E22Rik | RIKEN cDNA 1700056E22 gene |
| 1700065I16Rik | gasdermin pseudogene |
| 1700074H08Rik | RIKEN cDNA 1700074H08 gene |
| 1700094M24Rik | RIKEN cDNA 1700094M24 gene |
| 1700108F19Rik | RIKEN cDNA 1700108F19 gene |
| 1700126H18Rik | RIKEN cDNA 1700126H18 gene |
| 1810006J02Rik | RIKEN cDNA 1810006J02 gene |
| 1810013L24Rik | RIKEN cDNA 1810013L24 gene |
| 1810024B03Rik | RIKEN cDNA 1810024B03 gene |
| 1810065E05Rik | RIKEN cDNA 1810065E05 gene |
| 2010111I01Rik | RIKEN cDNA 2010111I01 gene |
| 2010204K13Rik | RIKEN cDNA 2010204K13 gene |
| 2210010C04Rik | RIKEN cDNA 2210010C04 gene |
| 2210414B05Rik | RIKEN cDNA 2210414B05 gene |
| 2410137M14Rik | RIKEN cDNA 2410137M14 gene |
| 2610020H08Rik | RIKEN cDNA 2610020H08 gene |
| 2700097O09Rik | RIKEN cDNA 2700097O09 gene |
| 2810001G20Rik | RIKEN cDNA 2810001G20 gene |
| 2810013P06Rik | RIKEN cDNA 2810013P06 gene |
| 2900011O08Rik | RIKEN cDNA 2900011O08 gene |
| 3830406C13Rik | RIKEN cDNA 3830406C13 gene |
| 4833427F10Rik | RIKEN cDNA 4833427F10 gene |
| 4833439L19Rik | RIKEN cDNA 4833439L19 gene |
| 4921509O07Rik | RIKEN cDNA 4921509O07 gene |
| 4921525O09Rik | RIKEN cDNA 4921525O09 gene |
| 4930404A05Rik | RIKEN cDNA 4930404A05 gene |
| 4930417O13Rik | RIKEN cDNA 4930417O13 gene |
| 4930430F21Rik | RIKEN cDNA 4930430F21 gene |
| 4930459C07Rik | RIKEN cDNA 4930459C07 gene |
| 4930487H11Rik | RIKEN cDNA 4930487H11 gene |
| 4930505K14Rik | RIKEN cDNA 4930505K14 gene |
| 4930519D14Rik | RIKEN cDNA 4930519D14 gene |
| 4930524N10Rik | RIKEN cDNA 4930524N10 gene |
| 4930529L06Rik | RIKEN cDNA 4930529L06 gene |
| 4930553E22Rik | RIKEN cDNA 4930553E22 gene |
| 4930554C24Rik | RIKEN cDNA 4930554C24 gene |
| 4930554G24Rik | RIKEN cDNA 4930554G24 gene |
| 4930556M19Rik | RIKEN cDNA 4930556M19 gene |
| 4930558F17Rik | RIKEN cDNA 4930558F17 gene |
| 4930559C10Rik | RIKEN cDNA 4930559C10 gene |
| 4930568D16Rik | RIKEN cDNA 4930568D16 gene |
| 4930572O13Rik | RIKEN cDNA 4930572O13 gene |
| 4930583K01Rik | RIKEN cDNA 4930583K01 gene |
| 4930590L20Rik | RIKEN cDNA 4930590L20 gene |
| 4931440P22Rik | RIKEN cDNA 4931440P22 gene |
| 4932414J04Rik | RIKEN cDNA 4932414J04 gene |
| 4932438H23Rik | RIKEN cDNA 4932438H23 gene |
| 4932443I19Rik | RIKEN cDNA 4932443I19 gene |
| 4933405E24Rik | RIKEN cDNA 4933405E24 gene |
| 4933411E08Rik | RIKEN cDNA 4933411E08 gene |
| 4933432G23Rik | RIKEN cDNA 4933432G23 gene |
| 4933433G19Rik | RIKEN cDNA 4933433G19 gene |
| 4933433H22Rik | RIKEN cDNA 4933433H22 gene |
| 4933440J02Rik | RIKEN cDNA 4933440J02 gene |
| 5031425F14Rik | RIKEN cDNA 5031425F14 gene |
| 5033428I22Rik | RIKEN cDNA 5033428I22 gene |
| 5430421F17Rik | RIKEN cDNA 5430421F17 gene |
| 5430427O19Rik | RIKEN cDNA 5430427O19 gene |
| 5430437J10Rik | RIKEN cDNA 5430437J10 gene |
| 5730460C07Rik | RIKEN cDNA 5730460C07 gene |
| 5730559C18Rik | RIKEN cDNA 5730559C18 gene |
| 5830428M24Rik | RIKEN cDNA 5830428M24 gene |
| 6530402F18Rik | RIKEN cDNA 6530402F18 gene |
| 8030442B05Rik | RIKEN cDNA 8030442B05 gene |
| 8430429K09Rik | RIKEN cDNA 8430429K09 gene |
| 8430430B14Rik | RIKEN cDNA 8430430B14 gene |
| 8430436N08Rik | RIKEN cDNA 8430436N08 gene |
| 9130015A21Rik | RIKEN cDNA 9130015A21 gene |
| 9430014N10Rik | RIKEN cDNA 9430014N10 gene |
| 9530026F06Rik | RIKEN cDNA 9530026F06 gene |
| 9930021J03Rik | RIKEN cDNA 9930021J03 gene |
| A1bg | alpha-1-B glycoprotein |
| A430035B10Rik | RIKEN cDNA A430035B10 gene |
| A530013C23Rik | RIKEN cDNA A530013C23 gene |
| A530058N18Rik | RIKEN cDNA A530058N18 gene |
| A930001A20Rik | RIKEN cDNA A930001A20 gene |
| Aak1 | AP2 associated kinase 1 |
| Abcb1b | ATP-binding cassette |
| Abcc2 | ATP-binding cassette |
| Abi2 | abl-interactor 2 |
| Ablim2 | actin-binding LIM protein 2 |
| Abo | ABO blood group (transferase A |
| Acaca | acetyl-Coenzyme A carboxylase alpha |
| Acadl | acyl-Coenzyme A dehydrogenase |
| Acadsb | acyl-Coenzyme A dehydrogenase |
| Acap2 | ArfGAP with coiled-coil |
| Acer1 | alkaline ceramidase 1 |
| Ache | acetylcholinesterase |
| Ackr2 | atypical chemokine receptor 2 |
| Acoxl | acyl-Coenzyme A oxidase-like |
| Acsl4 | acyl-CoA synthetase long-chain family member 4 |
| Acss2 | acyl-CoA synthetase short-chain family member 2 |
| Acss2os | acyl-CoA synthetase short-chain family member 2 |
| Acvr2a | activin receptor IIA |
| Acyp2 | acylphosphatase 2 |
| Adal | adenosine deaminase-like |
| Adam10 | a disintegrin and metallopeptidase domain 10 |
| Adam19 | a disintegrin and metallopeptidase domain 19 (meltrin beta) |
| Adam6b | a disintegrin and metallopeptidase domain 6B |
| Adam8 | a disintegrin and metallopeptidase domain 8 |
| Adamts20 | a disintegrin-like and metallopeptidase (reprolysin type) with thrombospondin type 1 motif |
| Adamtsl3 | ADAMTS-like 3 |
| Adamtsl4 | ADAMTS-like 4 |
| Adarb2 | adenosine deaminase |
| Adat1 | adenosine deaminase |
| Adcyap1 | adenylate cyclase activating polypeptide 1 |
| Add3 | adducin 3 (gamma) |
| Adgre5 | adhesion G protein-coupled receptor E5 |
| Adgrg1 | adhesion G protein-coupled receptor G1 |
| Adgrg6 | adhesion G protein-coupled receptor G6 |
| Adk | adenosine kinase |
| Ado | 2-aminoethanethiol (cysteamine) dioxygenase |
| Adrb2 | adrenergic receptor |
| Aebp2 | AE binding protein 2 |
| Aff2 | AF4/FMR2 family |
| Aff3 | AF4/FMR2 family |
| Afg3l2 | AFG3-like AAA ATPase 2 |
| Agfg2 | ArfGAP with FG repeats 2 |
| Agpat3 | 1-acylglycerol-3-phosphate O-acyltransferase 3 |
| Agpat4 | 1-acylglycerol-3-phosphate O-acyltransferase 4 (lysophosphatidic acid acyltransferase |
| Agrp | agouti related neuropeptide |
| Ahi1 | Abelson helper integration site 1 |
| AI197445 | expressed sequence AI197445 |
| Aim1l | absent in melanoma 1-like |
| Aim2 | absent in melanoma 2 |
| Akap13 | A kinase (PRKA) anchor protein 13 |
| Akap17b | A kinase (PRKA) anchor protein 17B |
| Akap7 | A kinase (PRKA) anchor protein 7 |
| Akap8l | A kinase (PRKA) anchor protein 8-like |
| Akirin1 | akirin 1 |
| Aknad1 | AKNA domain containing 1 |
| Akr1e1 | aldo-keto reductase family 1 |
| Akt2 | thymoma viral proto-oncogene 2 |
| Alcam | activated leukocyte cell adhesion molecule |
| Amfr | autocrine motility factor receptor |
| Amigo2 | adhesion molecule with Ig like domain 2 |
| Amph | amphiphysin |
| Anapc7 | anaphase promoting complex subunit 7 |
| Angpt1 | angiopoietin 1 |
| Ank2 | ankyrin 2 |
| Ank3 | ankyrin 3 |
| Ankmy1 | ankyrin repeat and MYND domain containing 1 |
| Ankrd16 | ankyrin repeat domain 16 |
| Ankrd55 | ankyrin repeat domain 55 |
| Anks1 | ankyrin repeat and SAM domain containing 1 |
| Anp32a | acidic (leucine-rich) nuclear phosphoprotein 32 family |
| Anp32b | acidic (leucine-rich) nuclear phosphoprotein 32 family |
| Antxr2 | anthrax toxin receptor 2 |
| Anxa1 | annexin A1 |
| Ap1b1 | adaptor protein complex AP-1 |
| Ap2a2 | adaptor-related protein complex 2 |
| Ap3s1 | adaptor-related protein complex 3 |
| Apbb1ip | amyloid beta (A4) precursor protein-binding |
| Apod | apolipoprotein D |
| Aqp4 | aquaporin 4 |
| Ar | androgen receptor |
| Arfip1 | ADP-ribosylation factor interacting protein 1 |
| Arg1 | arginase |
| Arglu1 | arginine and glutamate rich 1 |
| Arhgap10 | Rho GTPase activating protein 10 |
| Arhgap11a | Rho GTPase activating protein 11A |
| Arhgap15 | Rho GTPase activating protein 15 |
| Arhgap19 | Rho GTPase activating protein 19 |
| Arhgap26 | Rho GTPase activating protein 26 |
| Arhgap5 | Rho GTPase activating protein 5 |
| Arhgef10 | Rho guanine nucleotide exchange factor (GEF) 10 |
| Arhgef9 | CDC42 guanine nucleotide exchange factor (GEF) 9 |
| Arid1a | AT rich interactive domain 1A (SWI-like) |
| Arid2 | AT rich interactive domain 2 (ARID |
| Arid5b | AT rich interactive domain 5B (MRF1-like) |
| Arl15 | ADP-ribosylation factor-like 15 |
| Arl3 | ADP-ribosylation factor-like 3 |
| Arl4a | ADP-ribosylation factor-like 4A |
| Arl4c | ADP-ribosylation factor-like 4C |
| Arl8b | ADP-ribosylation factor-like 8B |
| Armc2 | armadillo repeat containing 2 |
| Armcx2 | armadillo repeat containing |
| Arnt2 | aryl hydrocarbon receptor nuclear translocator 2 |
| Arsb | arylsulfatase B |
| Art2a-ps | ADP-ribosyltransferase 2a |
| Art2b | ADP-ribosyltransferase 2b |
| Art4 | ADP-ribosyltransferase 4 |
| As3mt | arsenic (+3 oxidation state) methyltransferase |
| Asap2 | ArfGAP with SH3 domain |
| Asb1 | ankyrin repeat and SOCS box-containing 1 |
| Asb14 | ankyrin repeat and SOCS box-containing 14 |
| Asb4 | ankyrin repeat and SOCS box-containing 4 |
| Ascc1 | activating signal cointegrator 1 complex subunit 1 |
| Asmt | acetylserotonin O-methyltransferase |
| Asph | aspartate-beta-hydroxylase |
| Asprv1 | aspartic peptidase |
| Asrgl1 | asparaginase like 1 |
| Asxl2 | additional sex combs like 2 (Drosophila) |
| Atad1 | ATPase family |
| Atf7 | activating transcription factor 7 |
| Atg10 | autophagy related 10 |
| Atg3 | autophagy related 3 |
| Atg7 | autophagy related 7 |
| Atm | ataxia telangiectasia mutated |
| Atp10a | ATPase |
| Atp1a1 | ATPase |
| Atp2b1 | ATPase |
| Atp2c1 | ATPase |
| Atp8a2 | ATPase |
| Atp8b4 | ATPase |
| Atp8b5 | ATPase |
| Atrnl1 | attractin like 1 |
| Atxn1 | ataxin 1 |
| Atxn7l1 | ataxin 7-like 1 |
| AU022252 | expressed sequence AU022252 |
| Auh | AU RNA binding protein/enoyl-coenzyme A hydratase |
| Azi2 | 5-azacytidine induced gene 2 |
| B230208H11Rik | RIKEN cDNA B230208H11 gene |
| B230217O12Rik | RIKEN cDNA B230217O12 gene |
| B3galt2 | UDP-Gal:betaGlcNAc beta 1 |
| B4galnt2 | beta-1 |
| B4galnt3 | beta-1 |
| B4galnt4 | beta-1 |
| B4galt5 | UDP-Gal:betaGlcNAc beta 1 |
| B930018H19Rik | RIKEN cDNA B930018H19 gene |
| Bach2 | BTB and CNC homology |
| Bag3 | BCL2-associated athanogene 3 |
| Baiap2l1 | BAI1-associated protein 2-like 1 |
| Bard1 | BRCA1 associated RING domain 1 |
| Batf | basic leucine zipper transcription factor |
| Batf3 | basic leucine zipper transcription factor |
| Baz2b | bromodomain adjacent to zinc finger domain |
| Bbs9 | Bardet-Biedl syndrome 9 (human) |
| BC004004 | cDNA sequence BC004004 |
| Bcar1 | breast cancer anti-estrogen resistance 1 |
| Bcas3 | breast carcinoma amplified sequence 3 |
| Bcl10 | B cell leukemia/lymphoma 10 |
| Bcl2 | B cell leukemia/lymphoma 2 |
| Bcl2l11 | BCL2-like 11 (apoptosis facilitator) |
| Bcl7a | B cell CLL/lymphoma 7A |
| Bco1 | beta-carotene oxygenase 1 |
| Bco2 | beta-carotene oxygenase 2 |
| Best3 | bestrophin 3 |
| Bicd2 | bicaudal D homolog 2 (Drosophila) |
| Blk | B lymphoid kinase |
| Bmp2k | BMP2 inducible kinase |
| Bmpr1b | bone morphogenetic protein receptor |
| Braf | Braf transforming gene |
| Brd3 | bromodomain containing 3 |
| Bre | brain and reproductive organ-expressed protein |
| Brip1 | BRCA1 interacting protein C-terminal helicase 1 |
| Brwd1 | bromodomain and WD repeat domain containing 1 |
| Bsn | bassoon |
| Btbd11 | BTB (POZ) domain containing 11 |
| Btbd35f20 | BTB domain containing 35 |
| Btbd9 | BTB (POZ) domain containing 9 |
| Btf3l4 | basic transcription factor 3-like 4 |
| Btg1 | B cell translocation gene 1 |
| Btnl9 | butyrophilin-like 9 |
| Bvht | braveheart long non-coding RNA |
| C1qtnf6 | C1q and tumor necrosis factor related protein 6 |
| C2cd3 | C2 calcium-dependent domain containing 3 |
| C330011F03Rik | RIKEN cDNA C330011F03 gene |
| C430002N11Rik | RIKEN cDNA C430002N11 gene |
| C5ar1 | complement component 5a receptor 1 |
| C9 | complement component 9 |
| Cabin1 | calcineurin binding protein 1 |
| Cacna1b | calcium channel |
| Cacna1i | calcium channel |
| Cacng1 | calcium channel |
| Cacng2 | calcium channel |
| Cacng6 | calcium channel |
| Cacng8 | calcium channel |
| Cage1 | cancer antigen 1 |
| Calca | calcitonin/calcitonin-related polypeptide |
| Camk1d | calcium/calmodulin-dependent protein kinase ID |
| Camk2d | calcium/calmodulin-dependent protein kinase II |
| Capn2 | calpain 2 |
| Capn9 | calpain 9 |
| Car5b | carbonic anhydrase 5b |
| Card10 | caspase recruitment domain family |
| Carf | calcium response factor |
| Casp3 | caspase 3 |
| Casr | calcium-sensing receptor |
| Catip | ciliogenesis associated TTC17 interacting protein |
| Cbfa2t3 | core-binding factor |
| Cblb | Casitas B-lineage lymphoma b |
| Cbln2 | cerebellin 2 precursor protein |
| Ccdc121 | coiled-coil domain containing 121 |
| Ccdc148 | coiled-coil domain containing 148 |
| Ccdc160 | coiled-coil domain containing 160 |
| Ccdc162 | coiled-coil domain containing 162 |
| Ccdc187 | coiled-coil domain containing 187 |
| Ccdc58 | coiled-coil domain containing 58 |
| Ccdc6 | coiled-coil domain containing 6 |
| Ccdc69 | coiled-coil domain containing 69 |
| Ccdc80 | coiled-coil domain containing 80 |
| Ccdc88c | coiled-coil domain containing 88C |
| Cchcr1 | coiled-coil alpha-helical rod protein 1 |
| Ccl28 | chemokine (C-C motif) ligand 28 |
| Ccl3 | chemokine (C-C motif) ligand 3 |
| Ccl4 | chemokine (C-C motif) ligand 4 |
| Ccl5 | chemokine (C-C motif) ligand 5 |
| Ccl9 | chemokine (C-C motif) ligand 9 |
| Ccnd3 | cyclin D3 |
| Ccne1 | cyclin E1 |
| Ccnh | cyclin H |
| Ccnl1 | cyclin L1 |
| Ccr1l1 | chemokine (C-C motif) receptor 1-like 1 |
| Ccr2 | chemokine (C-C motif) receptor 2 |
| Ccr3 | chemokine (C-C motif) receptor 3 |
| Ccr9 | chemokine (C-C motif) receptor 9 |
| Ccser1 | coiled-coil serine rich 1 |
| Ccz1 | CCZ1 vacuolar protein trafficking and biogenesis associated |
| Cd226 | CD226 antigen |
| Cd274 | CD274 antigen |
| Cd302 | CD302 antigen |
| Cd320 | CD320 antigen |
| Cd38 | CD38 antigen |
| Cd44 | CD44 antigen |
| Cd55 | CD55 molecule |
| Cd5l | CD5 antigen-like |
| Cd6 | CD6 antigen |
| Cd7 | CD7 antigen |
| Cd86 | CD86 antigen |
| Cd96 | CD96 antigen |
| Cdadc1 | cytidine and dCMP deaminase domain containing 1 |
| Cdc20b | cell division cycle 20B |
| Cdc27 | cell division cycle 27 |
| Cdc42se2 | CDC42 small effector 2 |
| Cdca7l | cell division cycle associated 7 like |
| Cdh23 | cadherin 23 (otocadherin) |
| Cdk17 | cyclin-dependent kinase 17 |
| Cdk19 | cyclin-dependent kinase 19 |
| Cdk6 | cyclin-dependent kinase 6 |
| Cdkl3 | cyclin-dependent kinase-like 3 |
| Cdon | cell adhesion molecule-related/down-regulated by oncogenes |
| Cds2 | CDP-diacylglycerol synthase (phosphatidate cytidylyltransferase) 2 |
| Cdyl2 | chromodomain protein |
| Celf2 | CUGBP |
| Celsr1 | cadherin |
| Cenpc1 | centromere protein C1 |
| Cep192 | centrosomal protein 192 |
| Cep41 | centrosomal protein 41 |
| Cep57 | centrosomal protein 57 |
| Cep85l | centrosomal protein 85-like |
| Cep97 | centrosomal protein 97 |
| Cers4 | ceramide synthase 4 |
| Cers6 | ceramide synthase 6 |
| Cfap126 | cilia and flagella associated protein 126 |
| Cfdp1 | craniofacial development protein 1 |
| Chd7 | chromodomain helicase DNA binding protein 7 |
| Chic1 | cysteine-rich hydrophobic domain 1 |
| Chil5 | chitinase-like 5 |
| Chl1 | cell adhesion molecule L1-like |
| Chm | choroidermia (RAB escort protein 1) |
| Chmp4b | charged multivesicular body protein 4B |
| Chrna9 | cholinergic receptor |
| Chst10 | carbohydrate sulfotransferase 10 |
| Chst11 | carbohydrate sulfotransferase 11 |
| Chst2 | carbohydrate sulfotransferase 2 |
| Chsy1 | chondroitin sulfate synthase 1 |
| Cib3 | calcium and integrin binding family member 3 |
| Clasp2 | CLIP associating protein 2 |
| Cldn14 | claudin 14 |
| Cldn2 | claudin 2 |
| Clec12a | C-type lectin domain family 12 |
| Clec1a | C-type lectin domain family 1 |
| Clec1b | C-type lectin domain family 1 |
| Clec2g | C-type lectin domain family 2 |
| Clec2l | C-type lectin domain family 2 |
| Clic4 | chloride intracellular channel 4 (mitochondrial) |
| Clip1 | CAP-GLY domain containing linker protein 1 |
| Clmp | CXADR-like membrane protein |
| Cluap1 | clusterin associated protein 1 |
| Clybl | citrate lyase beta like |
| Cma1 | chymase 1 |
| Cmah | cytidine monophospho-N-acetylneuraminic acid hydroxylase |
| Cmc1 | COX assembly mitochondrial protein 1 |
| Cmip | c-Maf inducing protein |
| Cmklr1 | chemokine-like receptor 1 |
| Cmtm6 | CKLF-like MARVEL transmembrane domain containing 6 |
| Cnnm2 | cyclin M2 |
| Cnot6 | CCR4-NOT transcription complex |
| Cnr2 | cannabinoid receptor 2 (macrophage) |
| Cntln | centlein |
| Cobll1 | Cobl-like 1 |
| Coch | cochlin |
| Col8a2 | collagen |
| Commd3 | COMM domain containing 3 |
| Commd7 | COMM domain containing 7 |
| Copg2 | coatomer protein complex |
| Cops8 | COP9 signalosome subunit 8 |
| Coq10b | coenzyme Q10B |
| Coro2a | coronin |
| Coro2b | coronin |
| Cox10 | cytochrome c oxidase assembly protein 10 |
| Cpd | carboxypeptidase D |
| Cpn2 | carboxypeptidase N |
| Cpped1 | calcineurin-like phosphoesterase domain containing 1 |
| Cradd | CASP2 and RIPK1 domain containing adaptor with death domain |
| Creb1 | cAMP responsive element binding protein 1 |
| Crhbp | corticotropin releasing hormone binding protein |
| Crispld2 | cysteine-rich secretory protein LCCL domain containing 2 |
| Crlf3 | cytokine receptor-like factor 3 |
| Crmp1 | collapsin response mediator protein 1 |
| Crtc3 | CREB regulated transcription coactivator 3 |
| Cry1 | cryptochrome 1 (photolyase-like) |
| Cryba4 | crystallin |
| Crybb1 | crystallin |
| Csf1 | colony stimulating factor 1 (macrophage) |
| Csf2 | colony stimulating factor 2 (granulocyte-macrophage) |
| Csgalnact1 | chondroitin sulfate N-acetylgalactosaminyltransferase 1 |
| Ctcflos | CCCTC-binding factor (zinc finger protein)-like |
| Ctdp1 | CTD (carboxy-terminal domain |
| Cth | cystathionase (cystathionine gamma-lyase) |
| Ctla4 | cytotoxic T-lymphocyte-associated protein 4 |
| Ctnnd1 | catenin (cadherin associated protein) |
| Ctps2 | cytidine 5'-triphosphate synthase 2 |
| Ctsd | cathepsin D |
| Ctso | cathepsin O |
| Cuedc1 | CUE domain containing 1 |
| Cul3 | cullin 3 |
| Cx3cr1 | chemokine (C-X3-C motif) receptor 1 |
| Cxcl9 | chemokine (C-X-C motif) ligand 9 |
| Cyp2e1 | cytochrome P450 |
| Cyp2s1 | cytochrome P450 |
| Cyp4f16 | cytochrome P450 |
| Cyth1 | cytohesin 1 |
| Cytip | cytohesin 1 interacting protein |
| D030024E09Rik | RIKEN cDNA D030024E09 gene |
| D16Ertd472e | DNA segment |
| D17Wsu92e | DNA segment |
| D230030E09Rik | Riken cDNA D230030E09 gene |
| D330050G23Rik | RIKEN cDNA D330050G23 gene |
| D430041D05Rik | RIKEN cDNA D430041D05 gene |
| D830032E09Rik | RIKEN cDNA D830032E09 gene |
| D930015E06Rik | RIKEN cDNA D930015E06 gene |
| Dab2 | disabled 2 |
| Dab2ip | disabled 2 interacting protein |
| Dad1 | defender against cell death 1 |
| Dap | death-associated protein |
| Dapk2 | death-associated protein kinase 2 |
| Dars | aspartyl-tRNA synthetase |
| Dazl | deleted in azoospermia-like |
| Dcaf12 | DDB1 and CUL4 associated factor 12 |
| Dcaf5 | DDB1 and CUL4 associated factor 5 |
| Dclre1c | DNA cross-link repair 1C |
| Dcp1b | decapping mRNA 1B |
| Dcun1d3 | DCN1 |
| Dcun1d5 | DCN1 |
| Ddx43 | DEAD (Asp-Glu-Ala-Asp) box polypeptide 43 |
| Ddx59 | DEAD (Asp-Glu-Ala-Asp) box polypeptide 59 |
| Degs1 | delta(4)-desaturase |
| Dennd1b | DENN/MADD domain containing 1B |
| Dennd2c | DENN/MADD domain containing 2C |
| Depdc1a | DEP domain containing 1a |
| Desi2 | desumoylating isopeptidase 2 |
| Dgcr2 | DiGeorge syndrome critical region gene 2 |
| Dgkg | diacylglycerol kinase |
| Dgkh | diacylglycerol kinase |
| Dhcr7 | 7-dehydrocholesterol reductase |
| Dhrs9 | dehydrogenase/reductase (SDR family) member 9 |
| Diaph3 | diaphanous related formin 3 |
| Dip2b | disco interacting protein 2 homolog B |
| Disc1 | disrupted in schizophrenia 1 |
| Disp1 | dispatched RND transporter family member 1 |
| Dixdc1 | DIX domain containing 1 |
| Dkk3 | dickkopf WNT signaling pathway inhibitor 3 |
| Dld | dihydrolipoamide dehydrogenase |
| Dleu7 | deleted in lymphocytic leukemia |
| Dlg2 | discs |
| Dnah11 | dynein |
| Dnah6 | dynein |
| Dnah8 | dynein |
| Dnajb14 | DnaJ heat shock protein family (Hsp40) member B14 |
| Dnajc10 | DnaJ heat shock protein family (Hsp40) member C10 |
| Dnajc27 | DnaJ heat shock protein family (Hsp40) member C27 |
| Dock10 | dedicator of cytokinesis 10 |
| Dock2 | dedicator of cyto-kinesis 2 |
| Dock5 | dedicator of cytokinesis 5 |
| Dohh | deoxyhypusine hydroxylase/monooxygenase |
| Dok2 | docking protein 2 |
| Dok6 | docking protein 6 |
| Dpep2 | dipeptidase 2 |
| Dpy19l1 | dpy-19-like 1 (C. elegans) |
| Dstn | destrin |
| Dtd1 | D-tyrosyl-tRNA deacylase 1 |
| Dus2 | dihydrouridine synthase 2 |
| Dusp10 | dual specificity phosphatase 10 |
| Dusp16 | dual specificity phosphatase 16 |
| Dusp2 | dual specificity phosphatase 2 |
| Dusp6 | dual specificity phosphatase 6 |
| Dut | deoxyuridine triphosphatase |
| Dynll2 | dynein light chain LC8-type 2 |
| Dzank1 | double zinc ribbon and ankyrin repeat domains 1 |
| E030025P04Rik | RIKEN cDNA E030025P04 gene |
| E230029C05Rik | RIKEN cDNA E230029C05 gene |
| E2f3 | E2F transcription factor 3 |
| E430016F16Rik | RIKEN cDNA E430016F16 gene |
| Ebpl | emopamil binding protein-like |
| Ece1 | endothelin converting enzyme 1 |
| Edaradd | EDAR (ectodysplasin-A receptor)-associated death domain |
| Edem3 | ER degradation enhancer |
| Eed | embryonic ectoderm development |
| Efcab5 | EF-hand calcium binding domain 5 |
| Ehd2 | EH-domain containing 2 |
| Ehmt1 | euchromatic histone methyltransferase 1 |
| Eif4enif1 | eukaryotic translation initiation factor 4E nuclear import factor 1 |
| Eif4g3 | eukaryotic translation initiation factor 4 gamma |
| Eif5 | eukaryotic translation initiation factor 5 |
| Elac1 | elaC ribonuclease Z 1 |
| Elovl6 | ELOVL family member 6 |
| Emilin2 | elastin microfibril interfacer 2 |
| Enah | enabled homolog (Drosophila) |
| Enho | energy homeostasis associated |
| Entpd1 | ectonucleoside triphosphate diphosphohydrolase 1 |
| Entpd7 | ectonucleoside triphosphate diphosphohydrolase 7 |
| Epb41 | erythrocyte membrane protein band 4.1 |
| Epgn | epithelial mitogen |
| Epha7 | Eph receptor A7 |
| Ephx4 | epoxide hydrolase 4 |
| Eps8 | epidermal growth factor receptor pathway substrate 8 |
| Eps8l3 | EPS8-like 3 |
| Epx | eosinophil peroxidase |
| Erbb4 | erb-b2 receptor tyrosine kinase 4 |
| Ercc6l2 | excision repair cross-complementing rodent repair deficiency |
| Ergic1 | endoplasmic reticulum-golgi intermediate compartment (ERGIC) 1 |
| Erich5 | glutamate rich 5 |
| Ermap | erythroblast membrane-associated protein |
| Ermn | ermin |
| Ern1 | endoplasmic reticulum (ER) to nucleus signalling 1 |
| Esm1 | endothelial cell-specific molecule 1 |
| Ets1 | E26 avian leukemia oncogene 1 |
| Ets2 | E26 avian leukemia oncogene 2 |
| Etv5 | ets variant 5 |
| Exoc4 | exocyst complex component 4 |
| Exoc5 | exocyst complex component 5 |
| Exph5 | exophilin 5 |
| Eya3 | EYA transcriptional coactivator and phosphatase 3 |
| Ezr | ezrin |
| F630206G17Rik | RIKEN cDNA F630206G17 gene |
| F730043M19Rik | RIKEN cDNA F730043M19 gene |
| F9 | coagulation factor IX |
| Fabp12 | fatty acid binding protein 12 |
| Fabp5 | fatty acid binding protein 5 |
| Faf1 | Fas-associated factor 1 |
| Fam117b | family with sequence similarity 117 |
| Fam124b | family with sequence similarity 124 |
| Fam126a | family with sequence similarity 126 |
| Fam129a | family with sequence similarity 129 |
| Fam129b | family with sequence similarity 129 |
| Fam159b | family with sequence similarity 159 |
| Fam169b | family with sequence similarity 169 |
| Fam178b | family with sequence similarity 178 |
| Fam179a | family with sequence similarity 179 |
| Fam185a | family with sequence similarity 185 |
| Fam186b | family with sequence similarity 186 |
| Fam193a | family with sequence similarity 193 |
| Fam19a3 | family with sequence similarity 19 |
| Fam20a | family with sequence similarity 20 |
| Fam20c | family with sequence similarity 20 |
| Fam220a | family with sequence similarity 220 |
| Fam234a | family with sequence similarity 234 |
| Fam234b | family with sequence similarity 234 |
| Fam43b | family with sequence similarity 43 |
| Fam46a | family with sequence similarity 46 |
| Fam49a | family with sequence similarity 49 |
| Fam58b | family with sequence similarity 58 |
| Fam65c | family with sequence similarity 65 |
| Fam69a | family with sequence similarity 69 |
| Fam69b | family with sequence similarity 69 |
| Fanca | Fanconi anemia |
| Fancc | Fanconi anemia |
| Far1 | fatty acyl CoA reductase 1 |
| Fars2 | phenylalanine-tRNA synthetase 2 (mitochondrial) |
| Farsb | phenylalanyl-tRNA synthetase |
| Fasl | Fas ligand (TNF superfamily |
| Faxc | failed axon connections homolog |
| Fbxl2 | F-box and leucine-rich repeat protein 2 |
| Fbxl3 | F-box and leucine-rich repeat protein 3 |
| Fbxo27 | F-box protein 27 |
| Fbxo39 | F-box protein 39 |
| Fbxw8 | F-box and WD-40 domain protein 8 |
| Fcgr2b | Fc receptor |
| Fcho1 | FCH domain only 1 |
| Fcrl5 | Fc receptor-like 5 |
| Fcrl6 | Fc receptor-like 6 |
| Fcrla | Fc receptor-like A |
| Fcrls | Fc receptor-like S |
| Fdft1 | farnesyl diphosphate farnesyl transferase 1 |
| Ffar2 | free fatty acid receptor 2 |
| Fgf16 | fibroblast growth factor 16 |
| Fhl2 | four and a half LIM domains 2 |
| Fhl4 | four and a half LIM domains 4 |
| Filip1 | filamin A interacting protein 1 |
| Filip1l | filamin A interacting protein 1-like |
| Firre | functional intergenic repeating RNA element |
| Fkbp5 | FK506 binding protein 5 |
| Fkbp9 | FK506 binding protein 9 |
| Fmod | fibromodulin |
| Fnbp1 | formin binding protein 1 |
| Fnip2 | folliculin interacting protein 2 |
| Fntb | farnesyltransferase |
| Focad | focadhesin |
| Fosl2 | fos-like antigen 2 |
| Foxb1 | forkhead box B1 |
| Foxl1 | forkhead box L1 |
| Foxp1 | forkhead box P1 |
| Foxred2 | FAD-dependent oxidoreductase domain containing 2 |
| Frmpd4 | FERM and PDZ domain containing 4 |
| Frs2 | fibroblast growth factor receptor substrate 2 |
| Fryl | FRY like transcription coactivator |
| Furin | furin (paired basic amino acid cleaving enzyme) |
| Fut8 | fucosyltransferase 8 |
| Fyco1 | FYVE and coiled-coil domain containing 1 |
| G6pc | glucose-6-phosphatase |
| G730013B05Rik | RIKEN cDNA G730013B05 gene |
| Gab3 | growth factor receptor bound protein 2-associated protein 3 |
| Gabrr3 | gamma-aminobutyric acid (GABA) receptor |
| Gadd45g | growth arrest and DNA-damage-inducible 45 gamma |
| Galc | galactosylceramidase |
| Galnt1 | UDP-N-acetyl-alpha-D-galactosamine:polypeptide N-acetylgalactosaminyltransferase 1 |
| Galnt13 | UDP-N-acetyl-alpha-D-galactosamine:polypeptide N-acetylgalactosaminyltransferase 13 |
| Galnt14 | UDP-N-acetyl-alpha-D-galactosamine:polypeptide N-acetylgalactosaminyltransferase 14 |
| Galnt3 | UDP-N-acetyl-alpha-D-galactosamine:polypeptide N-acetylgalactosaminyltransferase 3 |
| Gas7 | growth arrest specific 7 |
| Gata3 | GATA binding protein 3 |
| Gatad2b | GATA zinc finger domain containing 2B |
| Gatsl2 | GATS protein-like 2 |
| Gbp10 | guanylate-binding protein 10 |
| Gbp2b | guanylate binding protein 2b |
| Gbp4 | guanylate binding protein 4 |
| Gcfc2 | GC-rich sequence DNA binding factor 2 |
| Gdpd5 | glycerophosphodiester phosphodiesterase domain containing 5 |
| Gfra1 | glial cell line derived neurotrophic factor family receptor alpha 1 |
| Gfra2 | glial cell line derived neurotrophic factor family receptor alpha 2 |
| Ggact | gamma-glutamylamine cyclotransferase |
| Ggps1 | geranylgeranyl diphosphate synthase 1 |
| Gimap3 | GTPase |
| Gipc2 | GIPC PDZ domain containing family |
| Gjb1 | gap junction protein |
| Glb1 | galactosidase |
| Glcci1 | glucocorticoid induced transcript 1 |
| Glmn | glomulin |
| Glod4 | glyoxalase domain containing 4 |
| Gltscr1 | glioma tumor suppressor candidate region gene 1 |
| Gm10069 | predicted gene 10069 |
| Gm10416 | zinc finger protein pseudogene |
| Gm1045 | predicted gene 1045 |
| Gm10787 | predicted gene 10787 |
| Gm11110 | predicted gene 11110 |
| Gm11468 | predicted gene 11468 |
| Gm12185 | predicted gene 12185 |
| Gm12216 | predicted gene 12216 |
| Gm12250 | predicted gene 12250 |
| Gm12596 | predicted gene 12596 |
| Gm12610 | predicted gene 12610 |
| Gm13830 | predicted gene 13830 |
| Gm14164 | predicted gene 14164 |
| Gm14718 | predicted gene 14718 |
| Gm156 | predicted gene 156 |
| Gm1661 | predicted gene 1661 |
| Gm16998 | predicted gene |
| Gm17746 | predicted gene |
| Gm19510 | predicted gene |
| Gm20098 | predicted gene |
| Gm20110 | predicted gene |
| Gm20139 | predicted gene |
| Gm20337 | predicted gene |
| Gm20362 | predicted gene |
| Gm20750 | predicted gene |
| Gm2447 | predicted gene 2447 |
| Gm27162 | predicted gene 27162 |
| Gm29685 | predicted gene |
| Gm29687 | predicted gene |
| Gm38404 | predicted gene |
| Gm38437 | predicted gene |
| Gm4251 | predicted gene 4251 |
| Gm4832 | predicted gene 4832 |
| Gm5086 | predicted gene 5086 |
| Gm5127 | predicted gene 5127 |
| Gm5148 | predicted gene 5148 |
| Gm5441 | predicted gene 5441 |
| Gm5523 | glyceraldehyde-3-phosphate dehydrogenase pseudogene |
| Gm5532 | predicted gene 5532 |
| Gm5535 | predicted gene 5535 |
| Gm6225 | predicted gene 6225 |
| Gm6455 | predicted gene 6455 |
| Gm8579 | spermatogenesis associated glutamate (E)-rich protein 4a pseudogene |
| Gm8817 | predicted gene 8817 |
| Gm8884 | predicted gene 8884 |
| Gm8989 | very large inducible GTPase 1 pseudogene |
| Gm9926 | predicted gene 9926 |
| Gna12 | guanine nucleotide binding protein |
| Gnaq | guanine nucleotide binding protein |
| Gnb1l | guanine nucleotide binding protein (G protein) |
| Golim4 | golgi integral membrane protein 4 |
| Gorab | golgin |
| Gosr1 | golgi SNAP receptor complex member 1 |
| Gpm6b | glycoprotein m6b |
| Gpr12 | G-protein coupled receptor 12 |
| Gpr174 | G protein-coupled receptor 174 |
| Gpr176 | G protein-coupled receptor 176 |
| Gpr55 | G protein-coupled receptor 55 |
| Gpsm2 | G-protein signalling modulator 2 (AGS3-like |
| Gpx8 | glutathione peroxidase 8 (putative) |
| Gramd3 | GRAM domain containing 3 |
| Grap2 | GRB2-related adaptor protein 2 |
| Grxcr1 | glutaredoxin |
| Gsap | gamma-secretase activating protein |
| Gsg1l | GSG1-like |
| Gstcd | glutathione S-transferase |
| Gtf2i | general transcription factor II I |
| Gxylt1 | glucoside xylosyltransferase 1 |
| Gyg | glycogenin |
| Gzmb | granzyme B |
| Gzmm | granzyme M (lymphocyte met-ase 1) |
| H2afy3 | H2A histone family |
| Haao | 3-hydroxyanthranilate 3 |
| Hadh | hydroxyacyl-Coenzyme A dehydrogenase |
| Hand1 | heart and neural crest derivatives expressed transcript 1 |
| Havcr2 | hepatitis A virus cellular receptor 2 |
| Hbs1l | Hbs1-like (S. cerevisiae) |
| Hc | hemolytic complement |
| Hcfc1 | host cell factor C1 |
| Hdac4 | histone deacetylase 4 |
| Heatr5a | HEAT repeat containing 5A |
| Heca | hdc homolog |
| Hectd1 | HECT domain containing 1 |
| Hecw2 | HECT |
| Heg1 | heart development protein with EGF-like domains 1 |
| Hells | helicase |
| Hemk1 | HemK methyltransferase family member 1 |
| Herc1 | HECT and RLD domain containing E3 ubiquitin protein ligase family member 1 |
| Herpud1 | homocysteine-inducible |
| Hhat | hedgehog acyltransferase |
| Hif1a | hypoxia inducible factor 1 |
| Hif1an | hypoxia-inducible factor 1 |
| Hipk2 | homeodomain interacting protein kinase 2 |
| Hivep2 | human immunodeficiency virus type I enhancer binding protein 2 |
| Hmbox1 | homeobox containing 1 |
| Hmg20a | high mobility group 20A |
| Hmgxb3 | HMG box domain containing 3 |
| Hmmr | hyaluronan mediated motility receptor (RHAMM) |
| Hmox1 | heme oxygenase 1 |
| Hnrnpa3 | heterogeneous nuclear ribonucleoprotein A3 |
| Hnrnpk | heterogeneous nuclear ribonucleoprotein K |
| Hnrnpul1 | heterogeneous nuclear ribonucleoprotein U-like 1 |
| Homer1 | homer scaffolding protein 1 |
| Hpse2 | heparanase 2 |
| Hs1bp3 | HCLS1 binding protein 3 |
| Hsbp1l1 | heat shock factor binding protein 1-like 1 |
| Hsd17b13 | hydroxysteroid (17-beta) dehydrogenase 13 |
| Huwe1 | HECT |
| Ica1 | islet cell autoantigen 1 |
| Icosl | icos ligand |
| Ifi203 | interferon activated gene 203 |
| Ifih1 | interferon induced with helicase C domain 1 |
| Ifitm1 | interferon induced transmembrane protein 1 |
| Ifng | interferon gamma |
| Ifngr1 | interferon gamma receptor 1 |
| Igbp1 | immunoglobulin (CD79A) binding protein 1 |
| Igf1r | insulin-like growth factor I receptor |
| Igf2r | insulin-like growth factor 2 receptor |
| Igfbp7 | insulin-like growth factor binding protein 7 |
| Ikzf2 | IKAROS family zinc finger 2 |
| Ikzf3 | IKAROS family zinc finger 3 |
| Il10 | interleukin 10 |
| Il10rb | interleukin 10 receptor |
| Il12b | interleukin 12b |
| Il12rb2 | interleukin 12 receptor |
| Il17a | interleukin 17A |
| Il17f | interleukin 17F |
| Il18r1 | interleukin 18 receptor 1 |
| Il18rap | interleukin 18 receptor accessory protein |
| Il1r1 | interleukin 1 receptor |
| Il1rap | interleukin 1 receptor accessory protein |
| Il23r | interleukin 23 receptor |
| Il27 | interleukin 27 |
| Il2ra | interleukin 2 receptor |
| Il31ra | interleukin 31 receptor A |
| Il33 | interleukin 33 |
| Il6 | interleukin 6 |
| Il6st | interleukin 6 signal transducer |
| Il7r | interleukin 7 receptor |
| Inpp4a | inositol polyphosphate-4-phosphatase |
| Insig1 | insulin induced gene 1 |
| Ints4 | integrator complex subunit 4 |
| Ints7 | integrator complex subunit 7 |
| Ipmk | inositol polyphosphate multikinase |
| Ipo11 | importin 11 |
| Iqcg | IQ motif containing G |
| Iqgap1 | IQ motif containing GTPase activating protein 1 |
| Iqgap3 | IQ motif containing GTPase activating protein 3 |
| Irf2 | interferon regulatory factor 2 |
| Irf2bp2 | interferon regulatory factor 2 binding protein 2 |
| Irf8 | interferon regulatory factor 8 |
| Itga1 | integrin alpha 1 |
| Itga11 | integrin alpha 11 |
| Itga2 | integrin alpha 2 |
| Itga3 | integrin alpha 3 |
| Itga4 | integrin alpha 4 |
| Itga9 | integrin alpha 9 |
| Itgad | integrin |
| Itgax | integrin alpha X |
| Itgb1 | integrin beta 1 (fibronectin receptor beta) |
| Itgb3 | integrin beta 3 |
| Itm2c | integral membrane protein 2C |
| Itpr1 | inositol 1 |
| Itpr2 | inositol 1 |
| Itpripl2 | inositol 1 |
| Itsn1 | intersectin 1 (SH3 domain protein 1A) |
| Itsn2 | intersectin 2 |
| Iyd | iodotyrosine deiodinase |
| Jak1 | Janus kinase 1 |
| Jaml | junction adhesion molecule like |
| Jdp2 | Jun dimerization protein 2 |
| Jpx | Jpx transcript |
| Jun | jun proto-oncogene |
| Kars | lysyl-tRNA synthetase |
| Kcna3 | potassium voltage-gated channel |
| Kcnc1 | potassium voltage gated channel |
| Kcng1 | potassium voltage-gated channel |
| Kcnh8 | potassium voltage-gated channel |
| Kcnip2 | Kv channel-interacting protein 2 |
| Kcnj15 | potassium inwardly-rectifying channel |
| Kcnj2 | potassium inwardly-rectifying channel |
| Kcnj3 | potassium inwardly-rectifying channel |
| Kcnj8 | potassium inwardly-rectifying channel |
| Kcnq5 | potassium voltage-gated channel |
| Kctd14 | potassium channel tetramerisation domain containing 14 |
| Kdm4a | lysine (K)-specific demethylase 4A |
| Kdm7a | lysine (K)-specific demethylase 7A |
| Kdm8 | lysine (K)-specific demethylase 8 |
| Kif20b | kinesin family member 20B |
| Kif23 | kinesin family member 23 |
| Kl | klotho |
| Klf13 | Kruppel-like factor 13 |
| Klf3 | Kruppel-like factor 3 (basic) |
| Klf4 | Kruppel-like factor 4 (gut) |
| Klf6 | Kruppel-like factor 6 |
| Klhl25 | kelch-like 25 |
| Klhl31 | kelch-like 31 |
| Klhl5 | kelch-like 5 |
| Klhl6 | kelch-like 6 |
| Klra1 | killer cell lectin-like receptor |
| Klra2 | killer cell lectin-like receptor |
| Klra22 | killer cell lectin-like receptor subfamily A |
| Klrb1 | killer cell lectin-like receptor subfamily B member 1 |
| Klrb1b | killer cell lectin-like receptor subfamily B member 1B |
| Klrb1f | killer cell lectin-like receptor subfamily B member 1F |
| Klrd1 | killer cell lectin-like receptor |
| Klre1 | killer cell lectin-like receptor family E member 1 |
| Klrg1 | killer cell lectin-like receptor subfamily G |
| Klrk1 | killer cell lectin-like receptor subfamily K |
| Kpnb1 | karyopherin (importin) beta 1 |
| Krt28 | keratin 28 |
| Krt42 | keratin 42 |
| Krt8 | keratin 8 |
| Ksr1 | kinase suppressor of ras 1 |
| Lair1 | leukocyte-associated Ig-like receptor 1 |
| Lama3 | laminin |
| Lamc1 | laminin |
| Lats2 | large tumor suppressor 2 |
| Lca5 | Leber congenital amaurosis 5 (human) |
| Lclat1 | lysocardiolipin acyltransferase 1 |
| Lcmt1 | leucine carboxyl methyltransferase 1 |
| Lcn4 | lipocalin 4 |
| Lef1 | lymphoid enhancer binding factor 1 |
| Lelp1 | late cornified envelope-like proline-rich 1 |
| Lhfpl1 | lipoma HMGIC fusion partner-like 1 |
| Lif | leukemia inhibitory factor |
| Lilr4b | leukocyte immunoglobulin-like receptor |
| Lilrb4a | leukocyte immunoglobulin-like receptor |
| Lim2 | lens intrinsic membrane protein 2 |
| Litaf | LPS-induced TN factor |
| Lmf1 | lipase maturation factor 1 |
| Lmo7 | LIM domain only 7 |
| Lncpint | long non-protein coding RNA |
| Lnpep | leucyl/cystinyl aminopeptidase |
| LOC106557447 | uncharacterized LOC106557447 |
| Lonp2 | lon peptidase 2 |
| Lpgat1 | lysophosphatidylglycerol acyltransferase 1 |
| Lpin1 | lipin 1 |
| Lpin2 | lipin 2 |
| Lpp | LIM domain containing preferred translocation partner in lipoma |
| Lrba | LPS-responsive beige-like anchor |
| Lrch3 | leucine-rich repeats and calponin homology (CH) domain containing 3 |
| Lrmp | lymphoid-restricted membrane protein |
| Lrrc20 | leucine rich repeat containing 20 |
| Lrrc71 | leucine rich repeat containing 71 |
| Lrrc74a | leucine rich repeat containing 74A |
| Lrrc8c | leucine rich repeat containing 8 family |
| Lrrc8d | leucine rich repeat containing 8D |
| Lrrfip1 | leucine rich repeat (in FLII) interacting protein 1 |
| Lrrk1 | leucine-rich repeat kinase 1 |
| Lrrk2 | leucine-rich repeat kinase 2 |
| Lrrn1 | leucine rich repeat protein 1 |
| Lsm6 | LSM6 homolog |
| Ltb4r2 | leukotriene B4 receptor 2 |
| Ly6c1 | lymphocyte antigen 6 complex |
| Ly75 | lymphocyte antigen 75 |
| Ly9 | lymphocyte antigen 9 |
| Ly96 | lymphocyte antigen 96 |
| Lyst | lysosomal trafficking regulator |
| Lyzl1 | lysozyme-like 1 |
| Lzic | leucine zipper and CTNNBIP1 domain containing |
| Macf1 | microtubule-actin crosslinking factor 1 |
| Maf | avian musculoaponeurotic fibrosarcoma oncogene homolog |
| Magi3 | membrane associated guanylate kinase |
| Magohb | mago homolog B |
| Magt1 | magnesium transporter 1 |
| Maml3 | mastermind like 3 (Drosophila) |
| Man1a | mannosidase 1 |
| Man1c1 | mannosidase |
| Manba | mannosidase |
| Map2k5 | mitogen-activated protein kinase kinase 5 |
| Map3k1 | mitogen-activated protein kinase kinase kinase 1 |
| Map3k2 | mitogen-activated protein kinase kinase kinase 2 |
| Map3k20 | mitogen-activated protein kinase kinase kinase 20 |
| Map3k5 | mitogen-activated protein kinase kinase kinase 5 |
| Map4k4 | mitogen-activated protein kinase kinase kinase kinase 4 |
| Mapk1 | mitogen-activated protein kinase 1 |
| Mapk15 | mitogen-activated protein kinase 15 |
| Mapkap1 | mitogen-activated protein kinase associated protein 1 |
| Mapre2 | microtubule-associated protein |
| 3-Mar | membrane-associated ring finger (C3HC4) 3 |
| Mars | methionine-tRNA synthetase |
| Mast2 | microtubule associated serine/threonine kinase 2 |
| Mast4 | microtubule associated serine/threonine kinase family member 4 |
| Max | Max protein |
| Mbnl1 | muscleblind-like 1 (Drosophila) |
| Mcm10 | minichromosome maintenance 10 replication initiation factor |
| Mctp2 | multiple C2 domains |
| Mdm1 | transformed mouse 3T3 cell double minute 1 |
| Med13 | mediator complex subunit 13 |
| Med13l | mediator complex subunit 13-like |
| Med4 | mediator complex subunit 4 |
| Mef2a | myocyte enhancer factor 2A |
| Mef2c | myocyte enhancer factor 2C |
| Megf10 | multiple EGF-like-domains 10 |
| Meioc | meiosis specific with coiled-coil domain |
| Metap2 | methionine aminopeptidase 2 |
| Metrnl | meteorin |
| Mettl24 | methyltransferase like 24 |
| Mettl7a1 | methyltransferase like 7A1 |
| Mex3c | mex3 RNA binding family member C |
| Mfsd4b2 | major facilitator superfamily domain containing 4B2 |
| Mfsd6l | major facilitator superfamily domain containing 6-like |
| Mgat4a | mannoside acetylglucosaminyltransferase 4 |
| Mgll | monoglyceride lipase |
| Mib1 | mindbomb E3 ubiquitin protein ligase 1 |
| Mier1 | MEIR1 treanscription regulator |
| Mir146 | microRNA 146 |
| Mir1892 | microRNA 1892 |
| Mir1893 | microRNA 1893 |
| Mir1907 | microRNA 1907 |
| Mir1929 | microRNA 1929 |
| Mir193a | microRNA 193a |
| Mir1967 | microRNA 1967 |
| Mir205 | microRNA 205 |
| Mir21c | microRNA 21c |
| Mir31 | microRNA 31 |
| Mir331 | microRNA 331 |
| Mir361 | microRNA 361 |
| Mir466p | microRNA 466p |
| Mir467h | microRNA 467h |
| Mir5118 | microRNA 5118 |
| Mir6337 | microRNA 6337 |
| Mir6361 | microRNA 6361 |
| Mir6373 | microRNA 6373 |
| Mir6387 | microRNA 6387 |
| Mir6388 | microRNA 6388 |
| Mir6906 | microRNA 6906 |
| Mir6937 | microRNA 6937 |
| Mir6951 | microRNA 6951 |
| Mir7094-1 | microRNA 7094-1 |
| Mir7094-2 | microRNA 7094-2 |
| Mir7216 | microRNA 7216 |
| Mir7218 | microRNA 7218 |
| Mir7237 | microRNA 7237 |
| Mir7661 | microRNA 7661 |
| Mir8096 | microRNA 8096 |
| Mirt1 | myocardial infarction associated transcript 1 |
| Mkl2 | MKL/myocardin-like 2 |
| Mknk2 | MAP kinase-interacting serine/threonine kinase 2 |
| Mkrn3 | makorin |
| Mllt1 | myeloid/lymphoid or mixed-lineage leukemia; translocated to |
| Mllt10 | myeloid/lymphoid or mixed-lineage leukemia; translocated to |
| Mmaa | methylmalonic aciduria (cobalamin deficiency) type A |
| Mmachc | methylmalonic aciduria cblC type |
| Mmp9 | matrix metallopeptidase 9 |
| Mms22l | MMS22-like |
| Mnd1 | meiotic nuclear divisions 1 |
| Moxd1 | monooxygenase |
| Mphosph9 | M-phase phosphoprotein 9 |
| Mplkip | M-phase specific PLK1 intereacting protein |
| Mpo | myeloperoxidase |
| Mpp6 | membrane protein |
| Mrpl1 | mitochondrial ribosomal protein L1 |
| Mrpl14 | mitochondrial ribosomal protein L14 |
| Mrpl16 | mitochondrial ribosomal protein L16 |
| Mrpl33 | mitochondrial ribosomal protein L33 |
| Mrvi1 | MRV integration site 1 |
| Ms4a4a | membrane-spanning 4-domains |
| Msh4 | mutS homolog 4 |
| Msi2 | musashi RNA-binding protein 2 |
| Mt3 | metallothionein 3 |
| Mt4 | metallothionein 4 |
| Mta3 | metastasis associated 3 |
| Mtap | methylthioadenosine phosphorylase |
| Mtg2 | mitochondrial ribosome associated GTPase 2 |
| Mthfd2l | methylenetetrahydrofolate dehydrogenase (NADP+ dependent) 2-like |
| Mthfs | 5 |
| Mtmr1 | myotubularin related protein 1 |
| Mtmr3 | myotubularin related protein 3 |
| Mtor | mechanistic target of rapamycin (serine/threonine kinase) |
| Mtpn | myotrophin |
| Mxi1 | MAX interactor 1 |
| Mybl2 | myeloblastosis oncogene-like 2 |
| Mybpc1 | myosin binding protein C |
| Myh10 | myosin |
| Myl1 | myosin |
| Mylip | myosin regulatory light chain interacting protein |
| Mylk | myosin |
| Mynn | myoneurin |
| Myo16 | myosin XVI |
| Myo1d | myosin ID |
| Myo6 | myosin VI |
| Myof | myoferlin |
| Myom2 | myomesin 2 |
| NA | NA |
| Nab1 | Ngfi-A binding protein 1 |
| Nabp1 | nucleic acid binding protein 1 |
| Nagk | N-acetylglucosamine kinase |
| Nap1l3 | nucleosome assembly protein 1-like 3 |
| Nav2 | neuron navigator 2 |
| Nbas | neuroblastoma amplified sequence |
| Ncapd3 | non-SMC condensin II complex |
| Ncaph | non-SMC condensin I complex |
| Ncbp1 | nuclear cap binding protein subunit 1 |
| Nccrp1 | non-specific cytotoxic cell receptor protein 1 homolog (zebrafish) |
| Nck1 | non-catalytic region of tyrosine kinase adaptor protein 1 |
| Nck2 | non-catalytic region of tyrosine kinase adaptor protein 2 |
| Nckap5 | NCK-associated protein 5 |
| Ncoa1 | nuclear receptor coactivator 1 |
| Ncoa2 | nuclear receptor coactivator 2 |
| Ndfip2 | Nedd4 family interacting protein 2 |
| Ndufa12 | NADH dehydrogenase (ubiquinone) 1 alpha subcomplex |
| Ndufa9 | NADH dehydrogenase (ubiquinone) 1 alpha subcomplex |
| Nedd4 | neural precursor cell expressed |
| Nedd4l | neural precursor cell expressed |
| Nedd9 | neural precursor cell expressed |
| Nek2 | NIMA (never in mitosis gene a)-related expressed kinase 2 |
| Nek7 | NIMA (never in mitosis gene a)-related expressed kinase 7 |
| Nemp2 | nuclear envelope integral membrane protein 2 |
| Nf2 | neurofibromin 2 |
| Nfat5 | nuclear factor of activated T cells 5 |
| Nfatc1 | nuclear factor of activated T cells |
| Nfatc2 | nuclear factor of activated T cells |
| Nfia | nuclear factor I/A |
| Nfil3 | nuclear factor |
| Nfkb1 | nuclear factor of kappa light polypeptide gene enhancer in B cells 1 |
| Nfkbiz | nuclear factor of kappa light polypeptide gene enhancer in B cells inhibitor |
| Nhlrc3 | NHL repeat containing 3 |
| Nhsl2 | NHS-like 2 |
| Nif3l1 | Ngg1 interacting factor 3-like 1 (S. pombe) |
| Ninj2 | ninjurin 2 |
| Nipal3 | NIPA-like domain containing 3 |
| Nkap | NFKB activating protein |
| Nkd1 | naked cuticle 1 homolog (Drosophila) |
| Nkx1-1 | NK1 transcription factor related |
| Nmnat2 | nicotinamide nucleotide adenylyltransferase 2 |
| Nmur1 | neuromedin U receptor 1 |
| Nnt | nicotinamide nucleotide transhydrogenase |
| Nob1 | NIN1/RPN12 binding protein 1 homolog |
| Nol10 | nucleolar protein 10 |
| Npl | N-acetylneuraminate pyruvate lyase |
| Nrarp | Notch-regulated ankyrin repeat protein |
| Nrip1 | nuclear receptor interacting protein 1 |
| Nsd1 | nuclear receptor-binding SET-domain protein 1 |
| Nsdhl | NAD(P) dependent steroid dehydrogenase-like |
| Nsmaf | neutral sphingomyelinase (N-SMase) activation associated factor |
| Nt5c2 | 5'-nucleotidase |
| Nt5dc3 | 5'-nucleotidase domain containing 3 |
| Nudt3 | nudix (nucleotide diphosphate linked moiety X)-type motif 3 |
| Nudt4 | nudix (nucleoside diphosphate linked moiety X)-type motif 4 |
| Numb | numb homolog (Drosophila) |
| Nup107 | nucleoporin 107 |
| Nupr1 | nuclear protein transcription regulator 1 |
| Nvl | nuclear VCP-like |
| Nyap2 | neuronal tyrosine-phophorylated phosphoinositide 3-kinase adaptor 2 |
| Odf1 | outer dense fiber of sperm tails 1 |
| Olfm3 | olfactomedin 3 |
| Olfr1423 | olfactory receptor 1423 |
| Olfr183 | olfactory receptor 183 |
| Olfr186 | olfactory receptor 186 |
| Olfr30 | olfactory receptor 30 |
| Olfr401 | olfactory receptor 401 |
| Olfr521 | olfactory receptor 521 |
| Olfr525 | olfactory receptor 525 |
| Olfr822 | olfactory receptor 822 |
| Optc | opticin |
| Osbp2 | oxysterol binding protein 2 |
| Osbpl3 | oxysterol binding protein-like 3 |
| Ostf1 | osteoclast stimulating factor 1 |
| Otogl | otogelin-like |
| Otud1 | OTU domain containing 1 |
| Oxnad1 | oxidoreductase NAD-binding domain containing 1 |
| Oxr1 | oxidation resistance 1 |
| Oxsr1 | oxidative-stress responsive 1 |
| Oxtr | oxytocin receptor |
| P2ry12 | purinergic receptor P2Y |
| P2ry13 | purinergic receptor P2Y |
| P2ry2 | purinergic receptor P2Y |
| P3h2 | prolyl 3-hydroxylase 2 |
| Pabpc1 | poly(A) binding protein |
| Pabpc2 | poly(A) binding protein |
| Pacs2 | phosphofurin acidic cluster sorting protein 2 |
| Pak4 | p21 protein (Cdc42/Rac)-activated kinase 4 |
| Pak6 | p21 protein (Cdc42/Rac)-activated kinase 6 |
| Pald1 | phosphatase domain containing |
| Pank1 | pantothenate kinase 1 |
| Pank3 | pantothenate kinase 3 |
| Paox | polyamine oxidase (exo-N4-amino) |
| Papd5 | PAP associated domain containing 5 |
| Park7 | Parkinson disease (autosomal recessive |
| Parl | presenilin associated |
| Parp1 | poly (ADP-ribose) polymerase family |
| Parp11 | poly (ADP-ribose) polymerase family |
| Parvg | parvin |
| Paxbp1 | PAX3 and PAX7 binding protein 1 |
| Pbx3 | pre B cell leukemia homeobox 3 |
| Pbx4 | pre B cell leukemia homeobox 4 |
| Pcdh7 | protocadherin 7 |
| Pced1b | PC-esterase domain containing 1B |
| Pcmtd1 | protein-L-isoaspartate (D-aspartate) O-methyltransferase domain containing 1 |
| Pdcl3 | phosducin-like 3 |
| Pde11a | phosphodiesterase 11A |
| Pde2a | phosphodiesterase 2A |
| Pde4a | phosphodiesterase 4A |
| Pde4b | phosphodiesterase 4B |
| Pde4d | phosphodiesterase 4D |
| Pde5a | phosphodiesterase 5A |
| Pde6d | phosphodiesterase 6D |
| Pde7a | phosphodiesterase 7A |
| Pdlim5 | PDZ and LIM domain 5 |
| Pdxdc1 | pyridoxal-dependent decarboxylase domain containing 1 |
| Pdzd2 | PDZ domain containing 2 |
| Peak1 | pseudopodium-enriched atypical kinase 1 |
| Pelo | pelota homolog (Drosophila) |
| Perp | PERP |
| Pgam1 | phosphoglycerate mutase 1 |
| Phactr4 | phosphatase and actin regulator 4 |
| Phf14 | PHD finger protein 14 |
| Phf2 | PHD finger protein 2 |
| Phf21a | PHD finger protein 21A |
| Phf6 | PHD finger protein 6 |
| Phf8 | PHD finger protein 8 |
| Phip | pleckstrin homology domain interacting protein |
| Phldb2 | pleckstrin homology like domain |
| Phrf1 | PHD and ring finger domains 1 |
| Piezo1 | piezo-type mechanosensitive ion channel component 1 |
| Piga | phosphatidylinositol glycan anchor biosynthesis |
| Pigk | phosphatidylinositol glycan anchor biosynthesis |
| Pigt | phosphatidylinositol glycan anchor biosynthesis |
| Pigv | phosphatidylinositol glycan anchor biosynthesis |
| Pik3ap1 | phosphoinositide-3-kinase adaptor protein 1 |
| Pik3cg | phosphoinositide-3-kinase |
| Pik3r1 | phosphatidylinositol 3-kinase |
| Pik3r3 | phosphatidylinositol 3 kinase |
| Pik3r6 | phosphoinositide-3-kinase |
| Pilrb1 | paired immunoglobin-like type 2 receptor beta 1 |
| Pim1 | proviral integration site 1 |
| Pip5k1b | phosphatidylinositol-4-phosphate 5-kinase |
| Pir | pirin |
| Pitpnc1 | phosphatidylinositol transfer protein |
| Pkn2 | protein kinase N2 |
| Pkp4 | plakophilin 4 |
| Pla2g10 | phospholipase A2 |
| Plat | plasminogen activator |
| Plcg2 | phospholipase C |
| Plcl1 | phospholipase C-like 1 |
| Plcl2 | phospholipase C-like 2 |
| Plek | pleckstrin |
| Plekha5 | pleckstrin homology domain containing |
| Plekhf1 | pleckstrin homology domain containing |
| Plekhg3 | pleckstrin homology domain containing |
| Plet1 | placenta expressed transcript 1 |
| Plet1os | placenta expressed transcript 1 |
| Plg | plasminogen |
| Plgrkt | plasminogen receptor |
| Plin2 | perilipin 2 |
| Plxna4 | plexin A4 |
| Plxnc1 | plexin C1 |
| Plxnd1 | plexin D1 |
| Pmaip1 | phorbol-12-myristate-13-acetate-induced protein 1 |
| Pnisr | PNN interacting serine/arginine-rich |
| Poc1a | POC1 centriolar protein A |
| Podxl | podocalyxin-like |
| Pofut2 | protein O-fucosyltransferase 2 |
| Pogk | pogo transposable element with KRAB domain |
| Pola1 | polymerase (DNA directed) |
| Pold2 | polymerase (DNA directed) |
| Polk | polymerase (DNA directed) |
| Polq | polymerase (DNA directed) |
| Polr3b | polymerase (RNA) III (DNA directed) polypeptide B |
| Pon2 | paraoxonase 2 |
| Ppa2 | pyrophosphatase (inorganic) 2 |
| Ppef2 | protein phosphatase |
| Pphln1 | periphilin 1 |
| Ppip5k2 | diphosphoinositol pentakisphosphate kinase 2 |
| Ppm1b | protein phosphatase 1B |
| Ppp1cb | protein phosphatase 1 |
| Ppp1r13b | protein phosphatase 1 |
| Ppp1r3b | protein phosphatase 1 |
| Ppp2r2c | protein phosphatase 2 |
| Ppp2r5a | protein phosphatase 2 |
| Ppp2r5c | protein phosphatase 2 |
| Ppp2r5d | protein phosphatase 2 |
| Ppp3ca | protein phosphatase 3 |
| Ppp4r1l-ps | protein phosphatase 4 |
| Ppp4r3a | protein phosphatase 4 regulatory subunit 3A |
| Prcp | prolylcarboxypeptidase (angiotensinase C) |
| Prdm1 | PR domain containing 1 |
| Prdx1 | peroxiredoxin 1 |
| Prep | prolyl endopeptidase |
| Prex1 | phosphatidylinositol-3 |
| Prkca | protein kinase C |
| Prkcb | protein kinase C |
| Prkce | protein kinase C |
| Prkcq | protein kinase C |
| Prkcsh | protein kinase C substrate 80K-H |
| Prkd3 | protein kinase D3 |
| Prkrip1 | Prkr interacting protein 1 (IL11 inducible) |
| Prkx | protein kinase |
| Prlr | prolactin receptor |
| Prmt3 | protein arginine N-methyltransferase 3 |
| Prmt7 | protein arginine N-methyltransferase 7 |
| Prok2 | prokineticin 2 |
| Prps2 | phosphoribosyl pyrophosphate synthetase 2 |
| Prpsap2 | phosphoribosyl pyrophosphate synthetase-associated protein 2 |
| Prrg1 | proline rich Gla (G-carboxyglutamic acid) 1 |
| Prrt4 | proline-rich transmembrane protein 4 |
| Prss12 | protease |
| Prss38 | protease |
| Pstpip1 | proline-serine-threonine phosphatase-interacting protein 1 |
| Ptafr | platelet-activating factor receptor |
| Ptch1 | patched 1 |
| Pten | phosphatase and tensin homolog |
| Ptgdr2 | prostaglandin D2 receptor 2 |
| Ptger2 | prostaglandin E receptor 2 (subtype EP2) |
| Ptger4 | prostaglandin E receptor 4 (subtype EP4) |
| Ptk2 | PTK2 protein tyrosine kinase 2 |
| Ptp4a2 | protein tyrosine phosphatase 4a2 |
| Ptpn1 | protein tyrosine phosphatase |
| Ptpn13 | protein tyrosine phosphatase |
| Ptpn2 | protein tyrosine phosphatase |
| Ptpn4 | protein tyrosine phosphatase |
| Ptpn5 | protein tyrosine phosphatase |
| Ptprc | protein tyrosine phosphatase |
| Ptprj | protein tyrosine phosphatase |
| Ptprk | protein tyrosine phosphatase |
| Ptprv | protein tyrosine phosphatase |
| Pum2 | pumilio RNA-binding family member 2 |
| Pus7 | pseudouridylate synthase 7 |
| Pvt1 | plasmacytoma variant translocation 1 |
| Pwwp2a | PWWP domain containing 2A |
| Pxylp1 | 2-phosphoxylose phosphatase 1 |
| Pygm | muscle glycogen phosphorylase |
| Qsox1 | quiescin Q6 sulfhydryl oxidase 1 |
| Rab11a | RAB11A |
| Rab28 | RAB28 |
| Rab2a | RAB2A |
| Rab39b | RAB39B |
| Rab3ip | RAB3A interacting protein |
| Rab44 | RAB44 |
| Rab4a | RAB4A |
| Rabgap1l | RAB GTPase activating protein 1-like |
| Rad50 | RAD50 double strand break repair protein |
| Rad51b | RAD51 paralog B |
| Rad54l2 | RAD54 like 2 (S. cerevisiae) |
| Rai14 | retinoic acid induced 14 |
| Ralgapa2 | Ral GTPase activating protein |
| Ralgapb | Ral GTPase activating protein |
| Ralgps2 | Ral GEF with PH domain and SH3 binding motif 2 |
| Raly | hnRNP-associated with lethal yellow |
| Ranbp9 | RAN binding protein 9 |
| Rap1a | RAS-related protein-1a |
| Rap1b | RAS related protein 1b |
| Rap1gap2 | RAP1 GTPase activating protein 2 |
| Rap1gds1 | RAP1 |
| Rap2a | RAS related protein 2a |
| Rapgef2 | Rap guanine nucleotide exchange factor (GEF) 2 |
| Rapgef6 | Rap guanine nucleotide exchange factor (GEF) 6 |
| Raph1 | Ras association (RalGDS/AF-6) and pleckstrin homology domains 1 |
| Rasa1 | RAS p21 protein activator 1 |
| Rasa3 | RAS p21 protein activator 3 |
| Rasal1 | RAS protein activator like 1 (GAP1 like) |
| Rasgef1c | RasGEF domain family |
| Rasgrf1 | RAS protein-specific guanine nucleotide-releasing factor 1 |
| Rasl11a | RAS-like |
| Rasl12 | RAS-like |
| Rb1 | RB transcriptional corepressor 1 |
| Rbm15 | RNA binding motif protein 15 |
| Rbm19 | RNA binding motif protein 19 |
| Rbm24 | RNA binding motif protein 24 |
| Rbm47 | RNA binding motif protein 47 |
| Rbm5 | RNA binding motif protein 5 |
| Rbms1 | RNA binding motif |
| Rbmx | RNA binding motif protein |
| Rbpj | recombination signal binding protein for immunoglobulin kappa J region |
| Rbpms2 | RNA binding protein with multiple splicing 2 |
| Rcbtb2 | regulator of chromosome condensation (RCC1) and BTB (POZ) domain containing protein 2 |
| Rcn1 | reticulocalbin 1 |
| Rcsd1 | RCSD domain containing 1 |
| Rdx | radixin |
| Relt | RELT tumor necrosis factor receptor |
| Reps1 | RalBP1 associated Eps domain containing protein |
| Rest | RE1-silencing transcription factor |
| Rftn1 | raftlin lipid raft linker 1 |
| Rfx3 | regulatory factor X |
| Rgs1 | regulator of G-protein signaling 1 |
| Rgs13 | regulator of G-protein signaling 13 |
| Rgs8 | regulator of G-protein signaling 8 |
| Rgs9 | regulator of G-protein signaling 9 |
| Rhobtb3 | Rho-related BTB domain containing 3 |
| Rhoh | ras homolog family member H |
| Rhoq | ras homolog family member Q |
| Rhov | ras homolog family member V |
| Rin3 | Ras and Rab interactor 3 |
| Rinl | Ras and Rab interactor-like |
| Riok1 | RIO kinase 1 (yeast) |
| Riox2 | ribosomal oxygenase 2 |
| Rmdn2 | regulator of microtubule dynamics 2 |
| Rnf138 | ring finger protein 138 |
| Rnf150 | ring finger protein 150 |
| Rnf19a | ring finger protein 19A |
| Rnft1 | ring finger protein |
| Rnls | renalase |
| Rnu6 | U6 small nuclear RNA |
| Rora | RAR-related orphan receptor alpha |
| Rpa2 | replication protein A2 |
| Rpap2 | RNA polymerase II associated protein 2 |
| Rpia | ribose 5-phosphate isomerase A |
| Rps6ka3 | ribosomal protein S6 kinase polypeptide 3 |
| Rps6ka5 | ribosomal protein S6 kinase |
| Rptor | regulatory associated protein of MTOR |
| Rras2 | related RAS viral (r-ras) oncogene 2 |
| Rreb1 | ras responsive element binding protein 1 |
| Rsf1os1 | remodeling and spacing factor 1 |
| Rsu1 | Ras suppressor protein 1 |
| Rtca | RNA 3'-terminal phosphate cyclase |
| Rtn4ip1 | reticulon 4 interacting protein 1 |
| Rubcnl | RUN and cysteine rich domain containing beclin 1 interacting protein like |
| Runx1 | runt related transcription factor 1 |
| Runx3 | runt related transcription factor 3 |
| Rxra | retinoid X receptor alpha |
| S1pr5 | sphingosine-1-phosphate receptor 5 |
| Samd12 | sterile alpha motif domain containing 12 |
| Samd3 | sterile alpha motif domain containing 3 |
| Samd4 | sterile alpha motif domain containing 4 |
| Samd9l | sterile alpha motif domain containing 9-like |
| Samsn1 | SAM domain |
| Sass6 | SAS-6 centriolar assembly protein |
| Satb1 | special AT-rich sequence binding protein 1 |
| Sbno1 | strawberry notch homolog 1 (Drosophila) |
| Scamp2 | secretory carrier membrane protein 2 |
| Scaper | S phase cyclin A-associated protein in the ER |
| Scarb2 | scavenger receptor class B |
| Scgb1a1 | secretoglobin |
| Scly | selenocysteine lyase |
| Scp2 | sterol carrier protein 2 |
| Scrg1 | scrapie responsive gene 1 |
| Sdccag8 | serologically defined colon cancer antigen 8 |
| Sdr39u1 | short chain dehydrogenase/reductase family 39U |
| Sec24d | Sec24 related gene family |
| Selenoi | selenoprotein I |
| Selenok | selenoprotein K |
| Sema4a | sema domain |
| Sema4b | sema domain |
| Sema6a | sema domain |
| Senp6 | SUMO/sentrin specific peptidase 6 |
| Senp7 | SUMO1/sentrin specific peptidase 7 |
| 1-Sep | septin 1 |
| 9-Sep | septin 9 |
| Serp2 | stress-associated endoplasmic reticulum protein family member 2 |
| Serpina3g | serine (or cysteine) peptidase inhibitor |
| Serpinb1a | serine (or cysteine) peptidase inhibitor |
| Serpinb1c | serine (or cysteine) peptidase inhibitor |
| Serping1 | serine (or cysteine) peptidase inhibitor |
| Setbp1 | SET binding protein 1 |
| Setd4 | SET domain containing 4 |
| Sf3b3 | splicing factor 3b |
| Sfmbt2 | Scm-like with four mbt domains 2 |
| Sgip1 | SH3-domain GRB2-like (endophilin) interacting protein 1 |
| Sgms1 | sphingomyelin synthase 1 |
| Sgsh | N-sulfoglucosamine sulfohydrolase (sulfamidase) |
| Sh2d1a | SH2 domain containing 1A |
| Sh2d4a | SH2 domain containing 4A |
| Sh3bgrl | SH3-binding domain glutamic acid-rich protein like |
| Sh3bp5 | SH3-domain binding protein 5 (BTK-associated) |
| Sh3glb1 | SH3-domain GRB2-like B1 (endophilin) |
| Shc3 | src homology 2 domain-containing transforming protein C3 |
| Shoc2 | soc-2 (suppressor of clear) homolog (C. elegans) |
| Shq1 | SHQ1 homolog (S. cerevisiae) |
| Siah1a | seven in absentia 1A |
| Sik1 | salt inducible kinase 1 |
| Sik2 | salt inducible kinase 2 |
| Sik3 | SIK family kinase 3 |
| Sipa1l1 | signal-induced proliferation-associated 1 like 1 |
| Skap1 | src family associated phosphoprotein 1 |
| Skint11 | selection and upkeep of intraepithelial T cells 11 |
| Sla2 | Src-like-adaptor 2 |
| Slamf1 | signaling lymphocytic activation molecule family member 1 |
| Slamf7 | SLAM family member 7 |
| Slc10a7 | solute carrier family 10 (sodium/bile acid cotransporter family) |
| Slc11a1 | solute carrier family 11 (proton-coupled divalent metal ion transporters) |
| Slc15a2 | solute carrier family 15 (H+/peptide transporter) |
| Slc16a10 | solute carrier family 16 (monocarboxylic acid transporters) |
| Slc16a11 | solute carrier family 16 (monocarboxylic acid transporters) |
| Slc16a3 | solute carrier family 16 (monocarboxylic acid transporters) |
| Slc17a5 | solute carrier family 17 (anion/sugar transporter) |
| Slc17a8 | solute carrier family 17 (sodium-dependent inorganic phosphate cotransporter) |
| Slc1a1 | solute carrier family 1 (neuronal/epithelial high affinity glutamate transporter |
| Slc1a5 | solute carrier family 1 (neutral amino acid transporter) |
| Slc22a8 | solute carrier family 22 (organic anion transporter) |
| Slc24a5 | solute carrier family 24 |
| Slc25a24 | solute carrier family 25 (mitochondrial carrier |
| Slc25a43 | solute carrier family 25 |
| Slc25a53 | solute carrier family 25 |
| Slc26a3 | solute carrier family 26 |
| Slc27a1 | solute carrier family 27 (fatty acid transporter) |
| Slc2a10 | solute carrier family 2 (facilitated glucose transporter) |
| Slc30a7 | solute carrier family 30 (zinc transporter) |
| Slc38a10 | solute carrier family 38 |
| Slc38a2 | solute carrier family 38 |
| Slc4a4 | solute carrier family 4 (anion exchanger) |
| Slc4a5 | solute carrier family 4 |
| Slc5a11 | solute carrier family 5 (sodium/glucose cotransporter) |
| Slc6a6 | solute carrier family 6 (neurotransmitter transporter |
| Slc9a1 | solute carrier family 9 (sodium/hydrogen exchanger) |
| Slc9a4 | solute carrier family 9 (sodium/hydrogen exchanger) |
| Slc9a9 | solute carrier family 9 (sodium/hydrogen exchanger) |
| Slco3a1 | solute carrier organic anion transporter family |
| Slco5a1 | solute carrier organic anion transporter family |
| Slitrk3 | SLIT and NTRK-like family |
| Slmap | sarcolemma associated protein |
| Slpi | secretory leukocyte peptidase inhibitor |
| Slx4ip | SLX4 interacting protein |
| Smad3 | SMAD family member 3 |
| Smap2 | small ArfGAP 2 |
| Smarcc1 | SWI/SNF related |
| Smc6 | structural maintenance of chromosomes 6 |
| Smg6 | Smg-6 homolog |
| Smim3 | small integral membrane protein 3 |
| Smpd4 | sphingomyelin phosphodiesterase 4 |
| Smpdl3a | sphingomyelin phosphodiesterase |
| Snap47 | synaptosomal-associated protein |
| Snhg5 | small nucleolar RNA host gene 5 |
| Snn | stannin |
| Snord12 | small nucleolar RNA |
| Snrnp25 | small nuclear ribonucleoprotein 25 (U11/U12) |
| Sntb1 | syntrophin |
| Snx10 | sorting nexin 10 |
| Snx13 | sorting nexin 13 |
| Snx29 | sorting nexin 29 |
| Snx9 | sorting nexin 9 |
| Sobp | sine oculis-binding protein homolog (Drosophila) |
| Sorbs1 | sorbin and SH3 domain containing 1 |
| Sorl1 | sortilin-related receptor |
| Sos1 | son of sevenless homolog 1 (Drosophila) |
| Sox5 | SRY (sex determining region Y)-box 5 |
| Sox6 | SRY (sex determining region Y)-box 6 |
| Sp1 | trans-acting transcription factor 1 |
| Sp100 | nuclear antigen Sp100 |
| Sp3 | trans-acting transcription factor 3 |
| Sp4 | trans-acting transcription factor 4 |
| Spast | spastin |
| Spata2 | spermatogenesis associated 2 |
| Spata25 | spermatogenesis associated 25 |
| Spata5 | spermatogenesis associated 5 |
| Spats2 | spermatogenesis associated |
| Spef2 | sperm flagellar 2 |
| Spg21 | SPG21 |
| Spire2 | spire homolog 2 (Drosophila) |
| Sppl3 | signal peptide peptidase 3 |
| Spr | sepiapterin reductase |
| Sptlc2 | serine palmitoyltransferase |
| Srbd1 | S1 RNA binding domain 1 |
| Srd5a2 | steroid 5 alpha-reductase 2 |
| Srgap2 | SLIT-ROBO Rho GTPase activating protein 2 |
| Srl | sarcalumenin |
| Srpk1 | serine/arginine-rich protein specific kinase 1 |
| Ss18 | SS18 |
| Ssbp2 | single-stranded DNA binding protein 2 |
| Ssbp3 | single-stranded DNA binding protein 3 |
| Ssh2 | slingshot homolog 2 (Drosophila) |
| St3gal2 | ST3 beta-galactoside alpha-2 |
| St3gal6 | ST3 beta-galactoside alpha-2 |
| St6galnac5 | ST6 (alpha-N-acetyl-neuraminyl-2 |
| St7 | suppression of tumorigenicity 7 |
| St8sia3 | ST8 alpha-N-acetyl-neuraminide alpha-2 |
| St8sia6 | ST8 alpha-N-acetyl-neuraminide alpha-2 |
| Stag3 | stromal antigen 3 |
| Stard10 | START domain containing 10 |
| Stard3nl | STARD3 N-terminal like |
| Stard7 | START domain containing 7 |
| Stat4 | signal transducer and activator of transcription 4 |
| Steap4 | STEAP family member 4 |
| Stim1 | stromal interaction molecule 1 |
| Stim2 | stromal interaction molecule 2 |
| Stk24 | serine/threonine kinase 24 |
| Stk32c | serine/threonine kinase 32C |
| Stk38 | serine/threonine kinase 38 |
| Stk38l | serine/threonine kinase 38 like |
| Stk39 | serine/threonine kinase 39 |
| Stn1 | STN1 |
| Stom | stomatin |
| Stx11 | syntaxin 11 |
| Stx8 | syntaxin 8 |
| Stxbp4 | syntaxin binding protein 4 |
| Stxbp5 | syntaxin binding protein 5 (tomosyn) |
| Styk1 | serine/threonine/tyrosine kinase 1 |
| Sub1 | SUB1 homolog (S. cerevisiae) |
| Sucla2 | succinate-Coenzyme A ligase |
| Suclg2 | succinate-Coenzyme A ligase |
| Sucnr1 | succinate receptor 1 |
| Suco | SUN domain containing ossification factor |
| Sugct | succinyl-CoA glutarate-CoA transferase |
| Sult2b1 | sulfotransferase family |
| Sun1 | Sad1 and UNC84 domain containing 1 |
| Suox | sulfite oxidase |
| Supt16 | suppressor of Ty 16 |
| Supt3 | suppressor of Ty 3 |
| Svop | SV2 related protein |
| Swap70 | SWA-70 protein |
| Swt1 | SWT1 RNA endoribonuclease homolog (S. cerevisiae) |
| Syap1 | synapse associated protein 1 |
| Syf2 | SYF2 homolog |
| Synj1 | synaptojanin 1 |
| Synrg | synergin |
| Syt8 | synaptotagmin VIII |
| Sytl3 | synaptotagmin-like 3 |
| Tab2 | TGF-beta activated kinase 1/MAP3K7 binding protein 2 |
| Taf3 | TATA-box binding protein associated factor 3 |
| Tagap | T cell activation Rho GTPase activating protein |
| Tanc1 | tetratricopeptide repeat |
| Tanc2 | tetratricopeptide repeat |
| Tango6 | transport and golgi organization 6 |
| Taok1 | TAO kinase 1 |
| Taok3 | TAO kinase 3 |
| Tardbp | TAR DNA binding protein |
| Tax1bp1 | Tax1 (human T cell leukemia virus type I) binding protein 1 |
| Tbc1d16 | TBC1 domain family |
| Tbc1d2 | TBC1 domain family |
| Tbc1d22b | TBC1 domain family |
| Tbc1d4 | TBC1 domain family |
| Tbc1d5 | TBC1 domain family |
| Tbcd | tubulin-specific chaperone d |
| Tbl1x | transducin (beta)-like 1 X-linked |
| Tbl1xr1 | transducin (beta)-like 1X-linked receptor 1 |
| Tbx21 | T-box 21 |
| Tcf20 | transcription factor 20 |
| Tcf7l1 | transcription factor 7 like 1 (T cell specific |
| Tecpr1 | tectonin beta-propeller repeat containing 1 |
| Tef | thyrotroph embryonic factor |
| Tex33 | testis expressed 33 |
| Tfdp2 | transcription factor Dp 2 |
| Tg | thyroglobulin |
| Tgfbr2 | transforming growth factor |
| Tgfbr3 | transforming growth factor |
| Tgm3 | transglutaminase 3 |
| Tgs1 | trimethylguanosine synthase 1 |
| Th | tyrosine hydroxylase |
| Thada | thyroid adenoma associated |
| Thap6 | THAP domain containing 6 |
| Thbs4 | thrombospondin 4 |
| Themis | thymocyte selection associated |
| Thsd7b | thrombospondin |
| Thy1 | thymus cell antigen 1 |
| Tiam1 | T cell lymphoma invasion and metastasis 1 |
| Tiam2 | T cell lymphoma invasion and metastasis 2 |
| Tigar | Trp53 induced glycolysis repulatory phosphatase |
| Tk2 | thymidine kinase 2 |
| Tle3 | transducin-like enhancer of split 3 |
| Tle4 | transducin-like enhancer of split 4 |
| Tlk1 | tousled-like kinase 1 |
| Tm2d1 | TM2 domain containing 1 |
| Tmco3 | transmembrane and coiled-coil domains 3 |
| Tmem114 | transmembrane protein 114 |
| Tmem116 | transmembrane protein 116 |
| Tmem123 | transmembrane protein 123 |
| Tmem131 | transmembrane protein 131 |
| Tmem163 | transmembrane protein 163 |
| Tmem164 | transmembrane protein 164 |
| Tmem17 | transmembrane protein 17 |
| Tmem176a | transmembrane protein 176A |
| Tmem176b | transmembrane protein 176B |
| Tmem181c-ps | transmembrane protein 181C |
| Tmem189 | transmembrane protein 189 |
| Tmem2 | transmembrane protein 2 |
| Tmem230 | transmembrane protein 230 |
| Tmem231 | transmembrane protein 231 |
| Tmem232 | transmembrane protein 232 |
| Tmem243 | transmembrane protein 243 |
| Tmem50b | transmembrane protein 50B |
| Tmem64 | transmembrane protein 64 |
| Tmem65 | transmembrane protein 65 |
| Tmevpg1 | Theiler's murine encephalomyelitis virus persistence candidate gene 1 |
| Tmlhe | trimethyllysine hydroxylase |
| Tmprss11c | transmembrane protease |
| Tmprss12 | transmembrane (C-terminal) protease |
| Tmprss13 | transmembrane protease |
| Tnfaip3 | tumor necrosis factor |
| Tnfrsf26 | tumor necrosis factor receptor superfamily |
| Tnfsf14 | tumor necrosis factor (ligand) superfamily |
| Tnfsf15 | tumor necrosis factor (ligand) superfamily |
| Tnik | TRAF2 and NCK interacting kinase |
| Tnip3 | TNFAIP3 interacting protein 3 |
| Tnrc6b | trinucleotide repeat containing 6b |
| Tob1 | transducer of ErbB-2.1 |
| Tom1 | target of myb1 trafficking protein |
| Top1 | topoisomerase (DNA) I |
| Top2a | topoisomerase (DNA) II alpha |
| Top2b | topoisomerase (DNA) II beta |
| Top3a | topoisomerase (DNA) III alpha |
| Topbp1 | topoisomerase (DNA) II binding protein 1 |
| Tox | thymocyte selection-associated high mobility group box |
| Tpk1 | thiamine pyrophosphokinase |
| Tpp2 | tripeptidyl peptidase II |
| Traf3ip1 | TRAF3 interacting protein 1 |
| Tram1 | translocating chain-associating membrane protein 1 |
| Tram2 | translocating chain-associating membrane protein 2 |
| Trerf1 | transcriptional regulating factor 1 |
| Trim13 | tripartite motif-containing 13 |
| Trim16 | tripartite motif-containing 16 |
| Trim24 | tripartite motif-containing 24 |
| Trim25 | tripartite motif-containing 25 |
| Trim34a | tripartite motif-containing 34A |
| Trim62 | tripartite motif-containing 62 |
| Trim72 | tripartite motif-containing 72 |
| Triobp | TRIO and F-actin binding protein |
| Trmt11 | tRNA methyltransferase 11 |
| Trp53bp2 | transformation related protein 53 binding protein 2 |
| Trp53rkb | transformation related protein 53 regulating kinase B |
| Trpm1 | transient receptor potential cation channel |
| Trpm2 | transient receptor potential cation channel |
| Trub2 | TruB pseudouridine (psi) synthase family member 2 |
| Tsen2 | tRNA splicing endonuclease subunit 2 |
| Tspan14 | tetraspanin 14 |
| Tspan2 | tetraspanin 2 |
| Tspan32 | tetraspanin 32 |
| Tspan5 | tetraspanin 5 |
| Tspan6 | tetraspanin 6 |
| Ttc17 | tetratricopeptide repeat domain 17 |
| Ttc27 | tetratricopeptide repeat domain 27 |
| Ttc28 | tetratricopeptide repeat domain 28 |
| Ttc37 | tetratricopeptide repeat domain 37 |
| Ttc7b | tetratricopeptide repeat domain 7B |
| Ttll11 | tubulin tyrosine ligase-like family |
| Ttll5 | tubulin tyrosine ligase-like family |
| Ttyh3 | tweety family member 3 |
| Tulp1 | tubby like protein 1 |
| Txk | TXK tyrosine kinase |
| Tyrobp | TYRO protein tyrosine kinase binding protein |
| Ubac2 | ubiquitin associated domain containing 2 |
| Ube2cbp | ubiquitin-conjugating enzyme E2C binding protein |
| Ube2e3 | ubiquitin-conjugating enzyme E2E 3 |
| Ube2f | ubiquitin-conjugating enzyme E2F (putative) |
| Ube2g2 | ubiquitin-conjugating enzyme E2G 2 |
| Ube2h | ubiquitin-conjugating enzyme E2H |
| Ubl3 | ubiquitin-like 3 |
| Ubr1 | ubiquitin protein ligase E3 component n-recognin 1 |
| Ubr3 | ubiquitin protein ligase E3 component n-recognin 3 |
| Ubr5 | ubiquitin protein ligase E3 component n-recognin 5 |
| Ubtd2 | ubiquitin domain containing 2 |
| Ubxn2b | UBX domain protein 2B |
| Ufc1 | ubiquitin-fold modifier conjugating enzyme 1 |
| Ulbp1 | UL16 binding protein 1 |
| Unc13b | unc-13 homolog B (C. elegans) |
| Unc5b | unc-5 netrin receptor B |
| Unc79 | unc-79 homolog (C. elegans) |
| Upf1 | UPF1 regulator of nonsense transcripts homolog (yeast) |
| Uqcrfs1 | ubiquinol-cytochrome c reductase |
| Urad | ureidoimidazoline (2-oxo-4-hydroxy-4-carboxy-5) decarboxylase |
| Urgcp | upregulator of cell proliferation |
| Usp15 | ubiquitin specific peptidase 15 |
| Usp20 | ubiquitin specific peptidase 20 |
| Usp30 | ubiquitin specific peptidase 30 |
| Usp37 | ubiquitin specific peptidase 37 |
| Usp48 | ubiquitin specific peptidase 48 |
| Usp6nl | USP6 N-terminal like |
| Ust | uronyl-2-sulfotransferase |
| Utp6 | UTP6 small subunit processome component |
| Uts2 | urotensin 2 |
| Uvrag | UV radiation resistance associated gene |
| Vamp4 | vesicle-associated membrane protein 4 |
| Vav3 | vav 3 oncogene |
| Vcan | versican |
| Vmn2r100 | vomeronasal 2 |
| Vmn2r79 | vomeronasal 2 |
| Vmn2r85 | vomeronasal 2 |
| Vmn2r86 | vomeronasal 2 |
| Vmn2r88 | vomeronasal 2 |
| Vmn2r95 | vomeronasal 2 |
| Vmn2r96 | vomeronasal 2 |
| Vmn2r97 | vomeronasal 2 |
| Vmp1 | vacuole membrane protein 1 |
| Vopp1 | vesicular |
| Vps13a | vacuolar protein sorting 13A |
| Vps13b | vacuolar protein sorting 13B |
| Vps26b | VPS26 retromer complex component B |
| Vps54 | VPS54 GARP complex subunit |
| Vps8 | VPS8 CORVET complex subunit |
| Vwc2 | von Willebrand factor C domain containing 2 |
| Vwf | Von Willebrand factor |
| Wars2 | tryptophanyl tRNA synthetase 2 (mitochondrial) |
| Washc3 | WASH complex subunit 3 |
| Wdr41 | WD repeat domain 41 |
| Wdr47 | WD repeat domain 47 |
| Wdr95 | WD40 repeat domain 95 |
| Wdtc1 | WD and tetratricopeptide repeats 1 |
| Wfdc21 | WAP four-disulfide core domain 21 |
| Wisp1 | WNT1 inducible signaling pathway protein 1 |
| Wnt10b | wingless-type MMTV integration site family |
| Wscd2 | WSC domain containing 2 |
| Wwox | WW domain-containing oxidoreductase |
| Xcl1 | chemokine (C motif) ligand 1 |
| Xirp1 | xin actin-binding repeat containing 1 |
| Xpnpep1 | X-prolyl aminopeptidase (aminopeptidase P) 1 |
| Xrcc4 | X-ray repair complementing defective repair in Chinese hamster cells 4 |
| Xrn2 | 5'-3' exoribonuclease 2 |
| Xylt1 | xylosyltransferase 1 |
| Yaf2 | YY1 associated factor 2 |
| Ypel2 | yippee-like 2 (Drosophila) |
| Ypel5 | yippee-like 5 (Drosophila) |
| Ythdf3 | YTH domain family 3 |
| Ywhaq | tyrosine 3-monooxygenase/tryptophan 5-monooxygenase activation protein |
| Zbtb16 | zinc finger and BTB domain containing 16 |
| Zbtb20 | zinc finger and BTB domain containing 20 |
| Zbtb38 | zinc finger and BTB domain containing 38 |
| Zbtb41 | zinc finger and BTB domain containing 41 |
| Zc3h12c | zinc finger CCCH type containing 12C |
| Zc3h13 | zinc finger CCCH type containing 13 |
| Zc3h7a | zinc finger CCCH type containing 7 A |
| Zdhhc20 | zinc finger |
| Zdhhc22 | zinc finger |
| Zdhhc7 | zinc finger |
| Zeb2 | zinc finger E-box binding homeobox 2 |
| Zfat | zinc finger and AT hook domain containing |
| Zfp236 | zinc finger protein 236 |
| Zfp24 | zinc finger protein 24 |
| Zfp330 | zinc finger protein 330 |
| Zfp362 | zinc finger protein 362 |
| Zfp366 | zinc finger protein 366 |
| Zfp395 | zinc finger protein 395 |
| Zfp414 | zinc finger protein 414 |
| Zfp438 | zinc finger protein 438 |
| Zfp473 | zinc finger protein 473 |
| Zfp512 | zinc finger protein 512 |
| Zfp57 | zinc finger protein 57 |
| Zfp608 | zinc finger protein 608 |
| Zfp652 | zinc finger protein 652 |
| Zfp664 | zinc finger protein 664 |
| Zfp949 | zinc finger protein 949 |
| Zfpm1 | zinc finger protein |
| Zmiz1 | zinc finger |
| Zranb3 | zinc finger |
| Zswim6 | zinc finger SWIM-type containing 6 |
