# Supplementary material for: The histone methyltransferase EZH2 primes the early differentiation of follicular helper T cells during acute viral infection
Source: Cell Mol Immunol. 2019 Mar 6;17(3):247–60. doi: 10.1038/s41423-019-0219-z (PMC7052164; doi:10.1038/s41423-019-0219-z)
Supplement: Supplementary file 4 — Supplementary table 2 [file 41423_2019_219_MOESM4_ESM.docx]

**Supplementary Table 2. Differential peaks in cluster 4 in Fig. 1c**

| SYMBOL | GENENAME |
| --- | --- |
| 0610005C13Rik | RIKEN cDNA 0610005C13 gene |
| 0610031O16Rik | RIKEN cDNA 0610031O16 gene |
| 0610040F04Rik | RIKEN cDNA 0610040F04 gene |
| 0610040J01Rik | RIKEN cDNA 0610040J01 gene |
| 1110008L16Rik | RIKEN cDNA 1110008L16 gene |
| 1110020A21Rik | RIKEN cDNA 1110020A21 gene |
| 1110032F04Rik | RIKEN cDNA 1110032F04 gene |
| 1110034G24Rik | RIKEN cDNA 1110034G24 gene |
| 1110059E24Rik | RIKEN cDNA 1110059E24 gene |
| 1300002E11Rik | RIKEN cDNA 1300002E11 gene |
| 1500009L16Rik | RIKEN cDNA 1500009L16 gene |
| 1600002D24Rik | RIKEN cDNA 1600002D24 gene |
| 1600014C10Rik | RIKEN cDNA 1600014C10 gene |
| 1700001F09Rik | RIKEN cDNA 1700001F09 gene |
| 1700007F19Rik | RIKEN cDNA 1700007F19 gene |
| 1700008K24Rik | RIKEN cDNA 1700008K24 gene |
| 1700010K23Rik | RIKEN cDNA 1700010K23 gene |
| 1700011I03Rik | RIKEN cDNA 1700011I03 gene |
| 1700012B09Rik | RIKEN cDNA 1700012B09 gene |
| 1700012H19Rik | RIKEN cDNA 1700012H19 gene |
| 1700012I11Rik | RIKEN cDNA 1700012I11 gene |
| 1700016C15Rik | RIKEN cDNA 1700016C15 gene |
| 1700016G22Rik | RIKEN cDNA 1700016G22 gene |
| 1700016K19Rik | RIKEN cDNA 1700016K19 gene |
| 1700016L21Rik | RIKEN cDNA 1700016L21 gene |
| 1700017B05Rik | RIKEN cDNA 1700017B05 gene |
| 1700017G19Rik | RIKEN cDNA 1700017G19 gene |
| 1700017J07Rik | RIKEN cDNA 1700017J07 gene |
| 1700017L05Rik | RIKEN cDNA 1700017L05 gene |
| 1700017N19Rik | RIKEN cDNA 1700017N19 gene |
| 1700018A04Rik | RIKEN cDNA 1700018A04 gene |
| 1700019E08Rik | RIKEN cDNA 1700019E08 gene |
| 1700020M21Rik | RIKEN cDNA 1700020M21 gene |
| 1700021F05Rik | RIKEN cDNA 1700021F05 gene |
| 1700024G13Rik | RIKEN cDNA 1700024G13 gene |
| 1700025F24Rik | RIKEN cDNA 1700025F24 gene |
| 1700025G04Rik | RIKEN cDNA 1700025G04 gene |
| 1700027F09Rik | RIKEN cDNA 1700027F09 gene |
| 1700028D13Rik | RIKEN cDNA 1700028D13 gene |
| 1700029N11Rik | RIKEN cDNA 1700029N11 gene |
| 1700030C10Rik | RIKEN cDNA 1700030C10 gene |
| 1700030F04Rik | RIKEN cDNA 1700030F04 gene |
| 1700031P21Rik | RIKEN cDNA 1700031P21 gene |
| 1700034G24Rik | RIKEN cDNA 1700034G24 gene |
| 1700034K08Rik | RIKEN cDNA 1700034K08 gene |
| 1700041M19Rik | RIKEN cDNA 1700041M19 gene |
| 1700042G15Rik | RIKEN cDNA 1700042G15 gene |
| 1700042O10Rik | RIKEN cDNA 1700042O10 gene |
| 1700044C05Rik | RIKEN cDNA 1700044C05 gene |
| 1700054A03Rik | RIKEN cDNA 1700054A03 gene |
| 1700054K19Rik | RIKEN cDNA 1700054K19 gene |
| 1700056E22Rik | RIKEN cDNA 1700056E22 gene |
| 1700060C20Rik | RIKEN cDNA 1700060C20 gene |
| 1700061F12Rik | RIKEN cDNA 1700061F12 gene |
| 1700061G19Rik | RIKEN cDNA 1700061G19 gene |
| 1700061I17Rik | RIKEN cDNA 1700061I17 gene |
| 1700064J06Rik | RIKEN cDNA 1700064J06 gene |
| 1700065I16Rik | gasdermin pseudogene |
| 1700072I22Rik | RIKEN cDNA 1700072I22 gene |
| 1700080N15Rik | RIKEN cDNA 1700080N15 gene |
| 1700086L19Rik | RIKEN cDNA 1700086L19 gene |
| 1700094M24Rik | RIKEN cDNA 1700094M24 gene |
| 1700104L18Rik | RIKEN cDNA 1700104L18 gene |
| 1700108F19Rik | RIKEN cDNA 1700108F19 gene |
| 1700109K24Rik | RIKEN cDNA 1700109K24 gene |
| 1700112E06Rik | RIKEN cDNA 1700112E06 gene |
| 1700112H15Rik | RIKEN cDNA 1700112H15 gene |
| 1700112J16Rik | RIKEN cDNA 1700112J16 gene |
| 1700113H08Rik | RIKEN cDNA 1700113H08 gene |
| 1700116B05Rik | RIKEN cDNA 1700116B05 gene |
| 1700121L16Rik | RIKEN cDNA 1700121L16 gene |
| 1700121N20Rik | RIKEN cDNA 1700121N20 gene |
| 1700123J17Rik | RIKEN cDNA 1700123J17 gene |
| 1700123M08Rik | RIKEN cDNA 1700123M08 gene |
| 1700123O12Rik | RIKEN cDNA 1700123O12 gene |
| 1810006J02Rik | RIKEN cDNA 1810006J02 gene |
| 1810007D17Rik | RIKEN cDNA 1810007D17 gene |
| 1810013L24Rik | RIKEN cDNA 1810013L24 gene |
| 1810041L15Rik | RIKEN cDNA 1810041L15 gene |
| 1810053B23Rik | RIKEN cDNA 1810053B23 gene |
| 2010009K17Rik | RIKEN cDNA 2010009K17 gene |
| 2010111I01Rik | RIKEN cDNA 2010111I01 gene |
| 2010300C02Rik | RIKEN cDNA 2010300C02 gene |
| 2210408I21Rik | RIKEN cDNA 2210408I21 gene |
| 2310010J17Rik | RIKEN cDNA 2310010J17 gene |
| 2310020H05Rik | RIKEN cDNA 2310020H05 gene |
| 2310022B05Rik | RIKEN cDNA 2310022B05 gene |
| 2310034O05Rik | RIKEN cDNA 2310034O05 gene |
| 2310035C23Rik | RIKEN cDNA 2310035C23 gene |
| 2310043O21Rik | RIKEN cDNA 2310043O21 gene |
| 2410004P03Rik | RIKEN cDNA 2410004P03 gene |
| 2410018L13Rik | RIKEN cDNA 2410018L13 gene |
| 2410089E03Rik | RIKEN cDNA 2410089E03 gene |
| 2510009E07Rik | RIKEN cDNA 2510009E07 gene |
| 2610035D17Rik | RIKEN cDNA 2610035D17 gene |
| 2610037D02Rik | RIKEN cDNA 2610037D02 gene |
| 2610206C17Rik | RIKEN cDNA 2610206C17 gene |
| 2610307P16Rik | RIKEN cDNA 2610307P16 gene |
| 2700049A03Rik | RIKEN cDNA 2700049A03 gene |
| 2810454H06Rik | RIKEN cDNA 2810454H06 gene |
| 2900026A02Rik | RIKEN cDNA 2900026A02 gene |
| 2900092D14Rik | RIKEN cDNA 2900092D14 gene |
| 3110009E18Rik | RIKEN cDNA 3110009E18 gene |
| 3110009F21Rik | RIKEN cDNA 3110009F21 gene |
| 3110039I08Rik | RIKEN cDNA 3110039I08 gene |
| 3110039M20Rik | RIKEN cDNA 3110039M20 gene |
| 3930402G23Rik | RIKEN cDNA 3930402G23 gene |
| 4732490B19Rik | RIKEN cDNA 4732490B19 gene |
| 4833420G17Rik | RIKEN cDNA 4833420G17 gene |
| 4921504E06Rik | RIKEN cDNA 4921504E06 gene |
| 4921509O07Rik | RIKEN cDNA 4921509O07 gene |
| 4921511I17Rik | RIKEN cDNA 4921511I17 gene |
| 4921517D22Rik | RIKEN cDNA 4921517D22 gene |
| 4921525O09Rik | RIKEN cDNA 4921525O09 gene |
| 4921531P14Rik | RIKEN cDNA 4921531P14 gene |
| 4922502D21Rik | RIKEN cDNA 4922502D21 gene |
| 4922502H24Rik | RIKEN cDNA 4922502H24 gene |
| 4930405L22Rik | RIKEN cDNA 4930405L22 gene |
| 4930406D18Rik | RIKEN cDNA 4930406D18 gene |
| 4930413E15Rik | RIKEN cDNA 4930413E15 gene |
| 4930413G21Rik | RIKEN cDNA 4930413G21 gene |
| 4930417O13Rik | RIKEN cDNA 4930417O13 gene |
| 4930425K10Rik | RIKEN cDNA 4930425K10 gene |
| 4930425L21Rik | RIKEN cDNA 4930425L21 gene |
| 4930425O10Rik | RIKEN cDNA 4930425O10 gene |
| 4930426L09Rik | RIKEN cDNA 4930426L09 gene |
| 4930428G15Rik | RIKEN cDNA 4930428G15 gene |
| 4930429D17Rik | RIKEN cDNA 4930429D17 gene |
| 4930429F11Rik | RIKEN cDNA 4930429F11 gene |
| 4930432M17Rik | RIKEN cDNA 4930432M17 gene |
| 4930440C22Rik | RIKEN cDNA 4930440C22 gene |
| 4930441H08Rik | RIKEN cDNA 4930441H08 gene |
| 4930445N18Rik | RIKEN cDNA 4930445N18 gene |
| 4930447C04Rik | RIKEN cDNA 4930447C04 gene |
| 4930447K03Rik | RIKEN cDNA 4930447K03 gene |
| 4930447M23Rik | RIKEN cDNA 4930447M23 gene |
| 4930449E01Rik | RIKEN cDNA 4930449E01 gene |
| 4930459C07Rik | RIKEN cDNA 4930459C07 gene |
| 4930465M20Rik | RIKEN cDNA 4930465M20 gene |
| 4930471C04Rik | RIKEN cDNA 4930471C04 gene |
| 4930479D17Rik | RIKEN cDNA 4930479D17 gene |
| 4930512M02Rik | RIKEN cDNA 4930512M02 gene |
| 4930515B02Rik | RIKEN cDNA 4930515B02 gene |
| 4930515G16Rik | myc induced nuclear antigen pseudogene |
| 4930518J21Rik | RIKEN cDNA 4930518J21 gene |
| 4930523C07Rik | RIKEN cDNA 4930523C07 gene |
| 4930524N10Rik | RIKEN cDNA 4930524N10 gene |
| 4930524O05Rik | RIKEN cDNA 4930524O05 gene |
| 4930529L06Rik | RIKEN cDNA 4930529L06 gene |
| 4930539C22Rik | RIKEN cDNA 4930539C22 gene |
| 4930544G11Rik | RIKEN cDNA 4930544G11 gene |
| 4930546C10Rik | RIKEN cDNA 4930546C10 gene |
| 4930552P12Rik | RIKEN cDNA 4930552P12 gene |
| 4930554C24Rik | RIKEN cDNA 4930554C24 gene |
| 4930554H23Rik | RIKEN cDNA 4930554H23 gene |
| 4930556N09Rik | RIKEN cDNA 4930556N09 gene |
| 4930557F10Rik | RIKEN cDNA 4930557F10 gene |
| 4930559C10Rik | RIKEN cDNA 4930559C10 gene |
| 4930563H07Rik | RIKEN cDNA 4930563H07 gene |
| 4930563M20Rik | RIKEN cDNA 4930563M20 gene |
| 4930564D02Rik | RIKEN cDNA 4930564D02 gene |
| 4930570G19Rik | RIKEN cDNA 4930570G19 gene |
| 4930572O13Rik | RIKEN cDNA 4930572O13 gene |
| 4930593A02Rik | RIKEN cDNA 4930593A02 gene |
| 4931403G20Rik | RIKEN cDNA 4931403G20 gene |
| 4931406P16Rik | RIKEN cDNA 4931406P16 gene |
| 4931419H13Rik | RIKEN cDNA 4931419H13 gene |
| 4932435O22Rik | RIKEN cDNA 4932435O22 gene |
| 4932438A13Rik | RIKEN cDNA 4932438A13 gene |
| 4932443I19Rik | RIKEN cDNA 4932443I19 gene |
| 4933405E24Rik | RIKEN cDNA 4933405E24 gene |
| 4933406D12Rik | RIKEN cDNA 4933406D12 gene |
| 4933406J10Rik | RIKEN cDNA 4933406J10 gene |
| 4933406K04Rik | RIKEN cDNA 4933406K04 gene |
| 4933407L21Rik | RIKEN cDNA 4933407L21 gene |
| 4933412E24Rik | RIKEN cDNA 4933412E24 gene |
| 4933427D14Rik | RIKEN cDNA 4933427D14 gene |
| 4933430H16Rik | RIKEN cDNA 4933430H16 gene |
| 4933430I17Rik | RIKEN cDNA 4933430I17 gene |
| 4933432G23Rik | RIKEN cDNA 4933432G23 gene |
| 4933433H22Rik | RIKEN cDNA 4933433H22 gene |
| 4933440J02Rik | RIKEN cDNA 4933440J02 gene |
| 4933440M02Rik | RIKEN cDNA 4933440M02 gene |
| 5330411J11Rik | RIKEN cDNA 5330411J11 gene |
| 5430403N17Rik | RIKEN cDNA 5430403N17 gene |
| 5430434I15Rik | RIKEN cDNA 5430434I15 gene |
| 5430437J10Rik | RIKEN cDNA 5430437J10 gene |
| 5730460C07Rik | RIKEN cDNA 5730460C07 gene |
| 5730508B09Rik | RIKEN cDNA 5730508B09 gene |
| 5830454E08Rik | RIKEN cDNA 5830454E08 gene |
| 5930412G12Rik | RIKEN cDNA 5930412G12 gene |
| 6030407O03Rik | RIKEN cDNA 6030407O03 gene |
| 6030458C11Rik | RIKEN cDNA 6030458C11 gene |
| 6030471H07Rik | RIKEN cDNA 6030471H07 gene |
| 6330410L21Rik | RIKEN cDNA 6330410L21 gene |
| 6430573F11Rik | RIKEN cDNA 6430573F11 gene |
| 8030423F21Rik | RIKEN cDNA 8030423F21 gene |
| 8030442B05Rik | RIKEN cDNA 8030442B05 gene |
| 8030451O07Rik | RIKEN cDNA 8030451O07 gene |
| 8430423G03Rik | RIKEN cDNA 8430423G03 gene |
| 8430430B14Rik | RIKEN cDNA 8430430B14 gene |
| 8430436N08Rik | RIKEN cDNA 8430436N08 gene |
| 9030617O03Rik | RIKEN cDNA 9030617O03 gene |
| 9130011E15Rik | RIKEN cDNA 9130011E15 gene |
| 9130015A21Rik | RIKEN cDNA 9130015A21 gene |
| 9130019P16Rik | RIKEN cDNA 9130019P16 gene |
| 9130024F11Rik | RIKEN cDNA 9130024F11 gene |
| 9130221F21Rik | RIKEN cDNA 9130221F21 gene |
| 9130227L01Rik | RIKEN cDNA 9130227L01 gene |
| 9130230L23Rik | RIKEN cDNA 9130230L23 gene |
| 9330111N05Rik | RIKEN cDNA 9330111N05 gene |
| 9330175E14Rik | RIKEN cDNA 9330175E14 gene |
| 9330188P03Rik | RIKEN cDNA 9330188P03 gene |
| 9430019J16Rik | RIKEN cDNA 9430019J16 gene |
| 9430078K24Rik | RIKEN cDNA 9430078K24 gene |
| 9530068E07Rik | RIKEN cDNA 9530068E07 gene |
| 9530077C05Rik | RIKEN cDNA 9530077C05 gene |
| 9630013K17Rik | RIKEN cDNA 9630013K17 gene |
| 9830132P13Rik | RIKEN cDNA 9830132P13 gene |
| a | nonagouti |
| A130077B15Rik | RIKEN cDNA A130077B15 gene |
| A1cf | APOBEC1 complementation factor |
| A230028O05Rik | RIKEN cDNA A230028O05 gene |
| A330076C08Rik | RIKEN cDNA A330076C08 gene |
| A330093E20Rik | RIKEN cDNA A330093E20 gene |
| A430078G23Rik | RIKEN cDNA A430078G23 gene |
| A530013C23Rik | RIKEN cDNA A530013C23 gene |
| A530046M15Rik | RIKEN cDNA A530046M15 gene |
| A530050N04Rik | RIKEN cDNA A530050N04 gene |
| A530058N18Rik | RIKEN cDNA A530058N18 gene |
| A630023P12Rik | RIKEN cDNA A630023P12 gene |
| A730020M07Rik | RIKEN cDNA A730020M07 gene |
| A730043L09Rik | RIKEN cDNA A730043L09 gene |
| A830019L24Rik | RIKEN cDNA A830019L24 gene |
| A830052D11Rik | RIKEN cDNA A830052D11 gene |
| A930001A20Rik | RIKEN cDNA A930001A20 gene |
| A930003O13Rik | RIKEN cDNA A930003O13 gene |
| A930005H10Rik | RIKEN cDNA A930005H10 gene |
| A930011G23Rik | RIKEN cDNA A930011G23 gene |
| A930019D19Rik | RIKEN cDNA A930019D19 gene |
| Aaed1 | AhpC/TSA antioxidant enzyme domain containing 1 |
| Abca1 | ATP-binding cassette |
| Abca5 | ATP-binding cassette |
| Abcb1a | ATP-binding cassette |
| Abcb7 | ATP-binding cassette |
| Abcb8 | ATP-binding cassette |
| Abcc4 | ATP-binding cassette |
| Abcd2 | ATP-binding cassette |
| Abcd3 | ATP-binding cassette |
| Abce1 | ATP-binding cassette |
| Abcg1 | ATP-binding cassette |
| Abcg3 | ATP-binding cassette |
| Abhd17c | abhydrolase domain containing 17C |
| Abhd2 | abhydrolase domain containing 2 |
| Abi1 | abl-interactor 1 |
| Abl1 | c-abl oncogene 1 |
| Ablim1 | actin-binding LIM protein 1 |
| Acadm | acyl-Coenzyme A dehydrogenase |
| Acmsd | amino carboxymuconate semialdehyde decarboxylase |
| Acot12 | acyl-CoA thioesterase 12 |
| Acot7 | acyl-CoA thioesterase 7 |
| Acsl1 | acyl-CoA synthetase long-chain family member 1 |
| Acsl3 | acyl-CoA synthetase long-chain family member 3 |
| Actbl2 | actin |
| Actn1 | actinin |
| Actn2 | actinin alpha 2 |
| Actn4 | actinin alpha 4 |
| Actr3 | ARP3 actin-related protein 3 |
| Acvr2a | activin receptor IIA |
| Adam23 | a disintegrin and metallopeptidase domain 23 |
| Adam29 | a disintegrin and metallopeptidase domain 29 |
| Adam30 | a disintegrin and metallopeptidase domain 30 |
| Adam6b | a disintegrin and metallopeptidase domain 6B |
| Adamts17 | a disintegrin-like and metallopeptidase (reprolysin type) with thrombospondin type 1 motif |
| Adamts20 | a disintegrin-like and metallopeptidase (reprolysin type) with thrombospondin type 1 motif |
| Adamts3 | a disintegrin-like and metallopeptidase (reprolysin type) with thrombospondin type 1 motif |
| Adamtsl1 | ADAMTS-like 1 |
| Adamtsl3 | ADAMTS-like 3 |
| Adamtsl4 | ADAMTS-like 4 |
| Adarb2 | adenosine deaminase |
| Adck1 | aarF domain containing kinase 1 |
| Adcy1 | adenylate cyclase 1 |
| Adcyap1r1 | adenylate cyclase activating polypeptide 1 receptor 1 |
| Add2 | adducin 2 (beta) |
| Adgra3 | adhesion G protein-coupled receptor A3 |
| Adgrb1 | adhesion G protein-coupled receptor B1 |
| Adgrf5 | adhesion G protein-coupled receptor F5 |
| Adgrl2 | adhesion G protein-coupled receptor L2 |
| Adgrv1 | adhesion G protein-coupled receptor V1 |
| Adh1 | alcohol dehydrogenase 1 (class I) |
| Adipoq | adiponectin |
| Adk | adenosine kinase |
| Adm | adrenomedullin |
| Adnp2 | ADNP homeobox 2 |
| Adra1b | adrenergic receptor |
| Adrb3 | adrenergic receptor |
| Adtrp | androgen dependent TFPI regulating protein |
| Aebp2 | AE binding protein 2 |
| Afap1 | actin filament associated protein 1 |
| Aff1 | AF4/FMR2 family |
| Aff3 | AF4/FMR2 family |
| Aftph | aftiphilin |
| Agbl1 | ATP/GTP binding protein-like 1 |
| Agbl4 | ATP/GTP binding protein-like 4 |
| Agfg1 | ArfGAP with FG repeats 1 |
| Aggf1 | angiogenic factor with G patch and FHA domains 1 |
| Agk | acylglycerol kinase |
| Agmat | agmatine ureohydrolase (agmatinase) |
| Ago2 | argonaute RISC catalytic subunit 2 |
| Agps | alkylglycerone phosphate synthase |
| Agr3 | anterior gradient 3 |
| Agtpbp1 | ATP/GTP binding protein 1 |
| Ahi1 | Abelson helper integration site 1 |
| Ahr | aryl-hydrocarbon receptor |
| AI115009 | expressed sequence AI115009 |
| AI427809 | expressed sequence AI427809 |
| AI464131 | expressed sequence AI464131 |
| AI662270 | expressed sequence AI662270 |
| Aig1 | androgen-induced 1 |
| Ak7 | adenylate kinase 7 |
| Akap13 | A kinase (PRKA) anchor protein 13 |
| Akap6 | A kinase (PRKA) anchor protein 6 |
| Akap7 | A kinase (PRKA) anchor protein 7 |
| Akap8l | A kinase (PRKA) anchor protein 8-like |
| Akap9 | A kinase (PRKA) anchor protein (yotiao) 9 |
| Akip1 | A kinase (PRKA) interacting protein 1 |
| Akirin2 | akirin 2 |
| Akp3 | alkaline phosphatase 3 |
| Akr1b7 | aldo-keto reductase family 1 |
| Akr1d1 | aldo-keto reductase family 1 |
| Akr1e1 | aldo-keto reductase family 1 |
| Aktip | thymoma viral proto-oncogene 1 interacting protein |
| Alas1 | aminolevulinic acid synthase 1 |
| Alb | albumin |
| Aldh1a2 | aldehyde dehydrogenase family 1 |
| Aldh1a3 | aldehyde dehydrogenase family 1 |
| Aldh1l1 | aldehyde dehydrogenase 1 family |
| Alg14 | asparagine-linked glycosylation 14 |
| Alg9 | asparagine-linked glycosylation 9 (alpha 1 |
| Alk | anaplastic lymphoma kinase |
| Alox12 | arachidonate 12-lipoxygenase |
| Alox5ap | arachidonate 5-lipoxygenase activating protein |
| Alpk2 | alpha-kinase 2 |
| Als2 | amyotrophic lateral sclerosis 2 (juvenile) |
| Als2cl | ALS2 C-terminal like |
| Alx4 | aristaless-like homeobox 4 |
| Ambra1 | autophagy/beclin 1 regulator 1 |
| Amer2 | APC membrane recruitment 2 |
| Amer3 | APC membrane recruitment 3 |
| Amigo1 | adhesion molecule with Ig like domain 1 |
| Amigo2 | adhesion molecule with Ig like domain 2 |
| Ammecr1 | Alport syndrome |
| Amn1 | antagonist of mitotic exit network 1 |
| Amotl1 | angiomotin-like 1 |
| Amotl2 | angiomotin-like 2 |
| Anapc4 | anaphase promoting complex subunit 4 |
| Ankdd1b | ankyrin repeat and death domain containing 1B |
| Ankef1 | ankyrin repeat and EF-hand domain containing 1 |
| Ankfy1 | ankyrin repeat and FYVE domain containing 1 |
| Ankrd11 | ankyrin repeat domain 11 |
| Ankrd12 | ankyrin repeat domain 12 |
| Ankrd17 | ankyrin repeat domain 17 |
| Ankrd33b | ankyrin repeat domain 33B |
| Ankrd46 | ankyrin repeat domain 46 |
| Ankrd50 | ankyrin repeat domain 50 |
| Ankrd55 | ankyrin repeat domain 55 |
| Ankrd6 | ankyrin repeat domain 6 |
| Anks1b | ankyrin repeat and sterile alpha motif domain containing 1B |
| Ano10 | anoctamin 10 |
| Ano4 | anoctamin 4 |
| Ano6 | anoctamin 6 |
| Antxr1 | anthrax toxin receptor 1 |
| Antxr2 | anthrax toxin receptor 2 |
| Anxa13 | annexin A13 |
| Anxa6 | annexin A6 |
| Aoc1 | amine oxidase |
| Ap1ar | adaptor-related protein complex 1 associated regulatory protein |
| Ap1g1 | adaptor protein complex AP-1 |
| Ap3b1 | adaptor-related protein complex 3 |
| Ap5m1 | adaptor-related protein complex 5 |
| Apaf1 | apoptotic peptidase activating factor 1 |
| Apba1 | amyloid beta (A4) precursor protein binding |
| Apba2 | amyloid beta (A4) precursor protein-binding |
| Apbb1ip | amyloid beta (A4) precursor protein-binding |
| Apc | adenomatosis polyposis coli |
| Apon | apolipoprotein N |
| App | amyloid beta (A4) precursor protein |
| Appl1 | adaptor protein |
| Arap2 | ArfGAP with RhoGAP domain |
| Arc | activity regulated cytoskeletal-associated protein |
| Arcn1 | archain 1 |
| Arf6 | ADP-ribosylation factor 6 |
| Arfip1 | ADP-ribosylation factor interacting protein 1 |
| Arhgap10 | Rho GTPase activating protein 10 |
| Arhgap11a | Rho GTPase activating protein 11A |
| Arhgap12 | Rho GTPase activating protein 12 |
| Arhgap15 | Rho GTPase activating protein 15 |
| Arhgap20os | Rho GTPase activating protein 20 |
| Arhgap24 | Rho GTPase activating protein 24 |
| Arhgap26 | Rho GTPase activating protein 26 |
| Arhgap30 | Rho GTPase activating protein 30 |
| Arhgap31 | Rho GTPase activating protein 31 |
| Arhgap32 | Rho GTPase activating protein 32 |
| Arhgap35 | Rho GTPase activating protein 35 |
| Arhgap39 | Rho GTPase activating protein 39 |
| Arhgap5 | Rho GTPase activating protein 5 |
| Arhgef10 | Rho guanine nucleotide exchange factor (GEF) 10 |
| Arhgef10l | Rho guanine nucleotide exchange factor (GEF) 10-like |
| Arhgef3 | Rho guanine nucleotide exchange factor (GEF) 3 |
| Arhgef38 | Rho guanine nucleotide exchange factor (GEF) 38 |
| Arhgef5 | Rho guanine nucleotide exchange factor (GEF) 5 |
| Arid1b | AT rich interactive domain 1B (SWI-like) |
| Arid2 | AT rich interactive domain 2 (ARID |
| Arid4b | AT rich interactive domain 4B (RBP1-like) |
| Arid5b | AT rich interactive domain 5B (MRF1-like) |
| Arih2 | ariadne RBR E3 ubiquitin protein ligase 2 |
| Arl14ep | ADP-ribosylation factor-like 14 effector protein |
| Arl4a | ADP-ribosylation factor-like 4A |
| Arl4c | ADP-ribosylation factor-like 4C |
| Arl5b | ADP-ribosylation factor-like 5B |
| Arl6ip6 | ADP-ribosylation factor-like 6 interacting protein 6 |
| Armcx1 | armadillo repeat containing |
| Arnt2 | aryl hydrocarbon receptor nuclear translocator 2 |
| Arntl | aryl hydrocarbon receptor nuclear translocator-like |
| Arpc4 | actin related protein 2/3 complex |
| Arpp21 | cyclic AMP-regulated phosphoprotein |
| Arrb1 | arrestin |
| Arsb | arylsulfatase B |
| Art3 | ADP-ribosyltransferase 3 |
| Art4 | ADP-ribosyltransferase 4 |
| Arvcf | armadillo repeat gene deleted in velocardiofacial syndrome |
| Arxes2 | adipocyte-related X-chromosome expressed sequence 2 |
| Asap1 | ArfGAP with SH3 domain |
| Asb1 | ankyrin repeat and SOCS box-containing 1 |
| Ascc2 | activating signal cointegrator 1 complex subunit 2 |
| Ascc3 | activating signal cointegrator 1 complex subunit 3 |
| Ascl2 | achaete-scute family bHLH transcription factor 2 |
| Aspm | asp (abnormal spindle)-like |
| Aspn | asporin |
| Asprv1 | aspartic peptidase |
| Astn2 | astrotactin 2 |
| Atf6 | activating transcription factor 6 |
| Atg10 | autophagy related 10 |
| Atg7 | autophagy related 7 |
| Atl2 | atlastin GTPase 2 |
| Atn1 | atrophin 1 |
| Atoh7 | atonal bHLH transcription factor 7 |
| Atoh8 | atonal bHLH transcription factor 8 |
| Atp10a | ATPase |
| Atp10d | ATPase |
| Atp13a2 | ATPase type 13A2 |
| Atp1a1 | ATPase |
| Atp1a2 | ATPase |
| Atp1b1 | ATPase |
| Atp1b3 | ATPase |
| Atp2a2 | ATPase |
| Atp2b3 | ATPase |
| Atp2c1 | ATPase |
| Atp4b | ATPase |
| Atp6v1g3 | ATPase |
| Atp7a | ATPase |
| Atp8a1 | ATPase |
| Atp8a2 | ATPase |
| Atp8b4 | ATPase |
| Atp9a | ATPase |
| Atp9b | ATPase |
| Atrnl1 | attractin like 1 |
| Atxn1 | ataxin 1 |
| Atxn10 | ataxin 10 |
| Atxn2 | ataxin 2 |
| AU016765 | expressed sequence AU016765 |
| AU019990 | expressed sequence AU019990 |
| Auh | AU RNA binding protein/enoyl-coenzyme A hydratase |
| Auts2 | autism susceptibility candidate 2 |
| Aven | apoptosis |
| Axin2 | axin 2 |
| AY702102 | cDNA sequence AY702102 |
| Azi2 | 5-azacytidine induced gene 2 |
| B230118H07Rik | RIKEN cDNA B230118H07 gene |
| B230208H11Rik | RIKEN cDNA B230208H11 gene |
| B230323A14Rik | RIKEN cDNA B230323A14 gene |
| B3galnt1 | UDP-GalNAc:betaGlcNAc beta 1 |
| B3galt2 | UDP-Gal:betaGlcNAc beta 1 |
| B3gnt2 | UDP-GlcNAc:betaGal beta-1 |
| B3gnt7 | UDP-GlcNAc:betaGal beta-1 |
| B430306N03Rik | RIKEN cDNA B430306N03 gene |
| B4galt1 | UDP-Gal:betaGlcNAc beta 1 |
| B4galt5 | UDP-Gal:betaGlcNAc beta 1 |
| B4galt6 | UDP-Gal:betaGlcNAc beta 1 |
| B930018H19Rik | RIKEN cDNA B930018H19 gene |
| B930025P03Rik | RIKEN cDNA B930025P03 gene |
| B930092H01Rik | RIKEN cDNA B930092H01 gene |
| Bach1 | BTB and CNC homology 1 |
| Bach2 | BTB and CNC homology |
| Bach2os | BTB and CNC homology 2 |
| Baiap2l1 | BAI1-associated protein 2-like 1 |
| Bard1 | BRCA1 associated RING domain 1 |
| Basp1 | brain abundant |
| Batf3 | basic leucine zipper transcription factor |
| Baz2b | bromodomain adjacent to zinc finger domain |
| BB123696 | expressed sequence BB123696 |
| Bbs10 | Bardet-Biedl syndrome 10 (human) |
| Bbx | bobby sox HMG box containing |
| BC035044 | cDNA sequence BC035044 |
| BC039771 | cDNA sequence BC039771 |
| BC049352 | cDNA sequence BC049352 |
| Bc1 | brain cytoplasmic RNA 1 |
| Bcam | basal cell adhesion molecule |
| Bcap29 | B cell receptor associated protein 29 |
| Bcar1 | breast cancer anti-estrogen resistance 1 |
| Bcar3 | breast cancer anti-estrogen resistance 3 |
| Bcas1os2 | breast carcinoma amplified sequence 1 |
| Bcat1 | branched chain aminotransferase 1 |
| Bckdhb | branched chain ketoacid dehydrogenase E1 |
| Bcl11b | B cell leukemia/lymphoma 11B |
| Bcl2 | B cell leukemia/lymphoma 2 |
| Bcl2l11 | BCL2-like 11 (apoptosis facilitator) |
| Bcl2l14 | BCL2-like 14 (apoptosis facilitator) |
| Bcl3 | B cell leukemia/lymphoma 3 |
| Bcl6 | B cell leukemia/lymphoma 6 |
| Bcl7c | B cell CLL/lymphoma 7C |
| Bcl9 | B cell CLL/lymphoma 9 |
| Bcor | BCL6 interacting corepressor |
| Bdh2 | 3-hydroxybutyrate dehydrogenase |
| Bean1 | brain expressed |
| Begain | brain-enriched guanylate kinase-associated |
| Bend7 | BEN domain containing 7 |
| Bicc1 | BicC family RNA binding protein 1 |
| Bicdl1 | BICD family like cargo adaptor 1 |
| Bid | BH3 interacting domain death agonist |
| Bin1 | bridging integrator 1 |
| Birc6 | baculoviral IAP repeat-containing 6 |
| Bmp2k | BMP2 inducible kinase |
| Bmp7 | bone morphogenetic protein 7 |
| Bmp8b | bone morphogenetic protein 8b |
| Bmper | BMP-binding endothelial regulator |
| Bmpr1b | bone morphogenetic protein receptor |
| Bmyc | brain expressed myelocytomatosis oncogene |
| Bnc1 | basonuclin 1 |
| Boc | biregional cell adhesion molecule-related/down-regulated by oncogenes (Cdon) binding protein |
| Bod1 | biorientation of chromosomes in cell division 1 |
| Boll | bol |
| Bpifc | BPI fold containing family C |
| Brdt | bromodomain |
| Bre | brain and reproductive organ-expressed protein |
| Brwd1 | bromodomain and WD repeat domain containing 1 |
| Btbd35f20 | BTB domain containing 35 |
| Btbd7 | BTB (POZ) domain containing 7 |
| Btbd9 | BTB (POZ) domain containing 9 |
| Btg1 | B cell translocation gene 1 |
| Btla | B and T lymphocyte associated |
| Btnl9 | butyrophilin-like 9 |
| C130026L21Rik | RIKEN cDNA C130026L21 gene |
| C130060K24Rik | RIKEN cDNA C130060K24 gene |
| C130074G19Rik | RIKEN cDNA C130074G19 gene |
| C1ql2 | complement component 1 |
| C1ql3 | C1q-like 3 |
| C1qtnf2 | C1q and tumor necrosis factor related protein 2 |
| C230014O12Rik | RIKEN cDNA C230014O12 gene |
| C230024C17Rik | RIKEN cDNA C230024C17 gene |
| C2cd4a | C2 calcium-dependent domain containing 4A |
| C2cd4b | C2 calcium-dependent domain containing 4B |
| C2cd4d | C2 calcium-dependent domain containing 4D |
| C2cd5 | C2 calcium-dependent domain containing 5 |
| C330004P14Rik | RIKEN cDNA C330004P14 gene |
| C330024C12Rik | RIKEN cDNA C330024C12 gene |
| C530044C16Rik | RIKEN cDNA C530044C16 gene |
| C730036E19Rik | RIKEN cDNA C730036E19 gene |
| C9 | complement component 9 |
| Cables1 | CDK5 and Abl enzyme substrate 1 |
| Cacna1b | calcium channel |
| Cacna1d | calcium channel |
| Cacna1i | calcium channel |
| Cacna2d1 | calcium channel |
| Cacna2d2 | calcium channel |
| Cacnb3 | calcium channel |
| Cacnb4 | calcium channel |
| Cadm1 | cell adhesion molecule 1 |
| Calcrl | calcitonin receptor-like |
| Calhm1 | calcium homeostasis modulator 1 |
| Calm1 | calmodulin 1 |
| Calm2 | calmodulin 2 |
| Calml3 | calmodulin-like 3 |
| Calr4 | calreticulin 4 |
| Camk1g | calcium/calmodulin-dependent protein kinase I gamma |
| Camk2d | calcium/calmodulin-dependent protein kinase II |
| Camk4 | calcium/calmodulin-dependent protein kinase IV |
| Camkmt | calmodulin-lysine N-methyltransferase |
| Camsap2 | calmodulin regulated spectrin-associated protein family |
| Cand1 | cullin associated and neddylation disassociated 1 |
| Canx | calnexin |
| Cap2 | CAP |
| Car8 | carbonic anhydrase 8 |
| Carm1 | coactivator-associated arginine methyltransferase 1 |
| Carmil1 | capping protein regulator and myosin 1 linker 1 |
| Cartpt | CART prepropeptide |
| Casz1 | castor zinc finger 1 |
| Cav1 | caveolin 1 |
| Cbfa2t3 | core-binding factor |
| Cblb | Casitas B-lineage lymphoma b |
| Cbln2 | cerebellin 2 precursor protein |
| Cbr4 | carbonyl reductase 4 |
| Ccdc105 | coiled-coil domain containing 105 |
| Ccdc141 | coiled-coil domain containing 141 |
| Ccdc148 | coiled-coil domain containing 148 |
| Ccdc149 | coiled-coil domain containing 149 |
| Ccdc150 | coiled-coil domain containing 150 |
| Ccdc152 | coiled-coil domain containing 152 |
| Ccdc162 | coiled-coil domain containing 162 |
| Ccdc170 | coiled-coil domain containing 170 |
| Ccdc172 | coiled-coil domain containing 172 |
| Ccdc185 | coiled-coil domain containing 185 |
| Ccdc191 | coiled-coil domain containing 191 |
| Ccdc3 | coiled-coil domain containing 3 |
| Ccdc33 | coiled-coil domain containing 33 |
| Ccdc57 | coiled-coil domain containing 57 |
| Ccdc6 | coiled-coil domain containing 6 |
| Ccdc60 | coiled-coil domain containing 60 |
| Ccdc80 | coiled-coil domain containing 80 |
| Ccdc88a | coiled coil domain containing 88A |
| Ccm2 | cerebral cavernous malformation 2 |
| Ccnc | cyclin C |
| Ccnd2 | cyclin D2 |
| Ccnd3 | cyclin D3 |
| Ccnh | cyclin H |
| Ccnjl | cyclin J-like |
| Ccnk | cyclin K |
| Ccp110 | centriolar coiled coil protein 110 |
| Ccr4 | chemokine (C-C motif) receptor 4 |
| Ccr5 | chemokine (C-C motif) receptor 5 |
| Ccr6 | chemokine (C-C motif) receptor 6 |
| Ccr7 | chemokine (C-C motif) receptor 7 |
| Ccser1 | coiled-coil serine rich 1 |
| Cd101 | CD101 antigen |
| Cd180 | CD180 antigen |
| Cd2 | CD2 antigen |
| Cd200 | CD200 antigen |
| Cd200r3 | CD200 receptor 3 |
| Cd226 | CD226 antigen |
| Cd28 | CD28 antigen |
| Cd2ap | CD2-associated protein |
| Cd300a | CD300A molecule |
| Cd300lb | CD300 molecule like family member B |
| Cd33 | CD33 antigen |
| Cd40lg | CD40 ligand |
| Cd44 | CD44 antigen |
| Cd47 | CD47 antigen (Rh-related antigen |
| Cd55 | CD55 molecule |
| Cd6 | CD6 antigen |
| Cd83 | CD83 antigen |
| Cdc20b | cell division cycle 20B |
| Cdc42bpa | CDC42 binding protein kinase alpha |
| Cdc42ep3 | CDC42 effector protein (Rho GTPase binding) 3 |
| Cdc7 | cell division cycle 7 (S. cerevisiae) |
| Cdca8 | cell division cycle associated 8 |
| Cdcp1 | CUB domain containing protein 1 |
| Cdh17 | cadherin 17 |
| Cdh22 | cadherin 22 |
| Cdh23 | cadherin 23 (otocadherin) |
| Cdh26 | cadherin-like 26 |
| Cdh4 | cadherin 4 |
| Cdh5 | cadherin 5 |
| Cdhr3 | cadherin-related family member 3 |
| Cdk1 | cyclin-dependent kinase 1 |
| Cdk14 | cyclin-dependent kinase 14 |
| Cdk17 | cyclin-dependent kinase 17 |
| Cdk19 | cyclin-dependent kinase 19 |
| Cdk6 | cyclin-dependent kinase 6 |
| Cdk8 | cyclin-dependent kinase 8 |
| Cdkal1 | CDK5 regulatory subunit associated protein 1-like 1 |
| Cdkn2a | cyclin-dependent kinase inhibitor 2A |
| Cdon | cell adhesion molecule-related/down-regulated by oncogenes |
| Cdx2 | caudal type homeobox 2 |
| Cdyl | chromodomain protein |
| Ceacam2 | carcinoembryonic antigen-related cell adhesion molecule 2 |
| Ceacam9 | carcinoembryonic antigen-related cell adhesion molecule 9 |
| Cebpa | CCAAT/enhancer binding protein (C/EBP) |
| Celf1 | CUGBP |
| Celf2 | CUGBP |
| Celf5 | CUGBP |
| Celrr | cerebellum expressed regulatory RNA |
| Cenpf | centromere protein F |
| Cenpu | centromere protein U |
| Cep128 | centrosomal protein 128 |
| Cep135 | centrosomal protein 135 |
| Cep164 | centrosomal protein 164 |
| Cep41 | centrosomal protein 41 |
| Cep63 | centrosomal protein 63 |
| Cep85l | centrosomal protein 85-like |
| Cers3 | ceramide synthase 3 |
| Cers4 | ceramide synthase 4 |
| Cers6 | ceramide synthase 6 |
| Cfap20 | cilia and flagella associated protein 20 |
| Cfap53 | cilia and flagella associated protein 53 |
| Cfap70 | cilia and flagella associated protein 70 |
| Cfap97 | cilia and flagella associated protein 97 |
| Cfc1 | cripto |
| Cga | glycoprotein hormones |
| Cgnl1 | cingulin-like 1 |
| Chchd3 | coiled-coil-helix-coiled-coil-helix domain containing 3 |
| Chd2 | chromodomain helicase DNA binding protein 2 |
| Chd6 | chromodomain helicase DNA binding protein 6 |
| Chd7 | chromodomain helicase DNA binding protein 7 |
| Chdh | choline dehydrogenase |
| Chil3 | chitinase-like 3 |
| Chl1 | cell adhesion molecule L1-like |
| Chm | choroidermia (RAB escort protein 1) |
| Chn1os3 | chimerin 1 |
| Chn2 | chimerin 2 |
| Chrm3 | cholinergic receptor |
| Chrna5 | cholinergic receptor |
| Chrnb3 | cholinergic receptor |
| Chst1 | carbohydrate (keratan sulfate Gal-6) sulfotransferase 1 |
| Chst11 | carbohydrate sulfotransferase 11 |
| Chst14 | carbohydrate (N-acetylgalactosamine 4-0) sulfotransferase 14 |
| Chst15 | carbohydrate (N-acetylgalactosamine 4-sulfate 6-O) sulfotransferase 15 |
| Chst2 | carbohydrate sulfotransferase 2 |
| Chuk | conserved helix-loop-helix ubiquitous kinase |
| Cit | citron |
| Cited2 | Cbp/p300-interacting transactivator |
| Clasp1 | CLIP associating protein 1 |
| Clcc1 | chloride channel CLIC-like 1 |
| Cldn10 | claudin 10 |
| Cldn11 | claudin 11 |
| Cldn34b1 | claudin 34B1 |
| Cldn5 | claudin 5 |
| Clec12a | C-type lectin domain family 12 |
| Clec16a | C-type lectin domain family 16 |
| Clic5 | chloride intracellular channel 5 |
| Clint1 | clathrin interactor 1 |
| Clock | circadian locomotor output cycles kaput |
| Clpb | ClpB caseinolytic peptidase B |
| Clrn3 | clarin 3 |
| Clstn3 | calsyntenin 3 |
| Clvs1 | clavesin 1 |
| Cmah | cytidine monophospho-N-acetylneuraminic acid hydroxylase |
| Cmc1 | COX assembly mitochondrial protein 1 |
| Cmip | c-Maf inducing protein |
| Cmss1 | cms small ribosomal subunit 1 |
| Cngb1 | cyclic nucleotide gated channel beta 1 |
| Cnih3 | cornichon family AMPA receptor auxiliary protein 3 |
| Cnih4 | cornichon family AMPA receptor auxiliary protein 4 |
| Cnksr3 | Cnksr family member 3 |
| Cnn3 | calponin 3 |
| Cnot2 | CCR4-NOT transcription complex |
| Cnot6 | CCR4-NOT transcription complex |
| Cnr1 | cannabinoid receptor 1 (brain) |
| Cnr2 | cannabinoid receptor 2 (macrophage) |
| Cnst | consortin |
| Cntn4 | contactin 4 |
| Cntn5 | contactin 5 |
| Cobll1 | Cobl-like 1 |
| Cog5 | component of oligomeric golgi complex 5 |
| Cog7 | component of oligomeric golgi complex 7 |
| Col12a1 | collagen |
| Col15a1 | collagen |
| Col19a1 | collagen |
| Col26a1 | collagen |
| Col4a3 | collagen |
| Col5a3 | collagen |
| Colq | collagen-like tail subunit (single strand of homotrimer) of asymmetric acetylcholinesterase |
| Commd7 | COMM domain containing 7 |
| Copb1 | coatomer protein complex |
| Cops2 | COP9 signalosome subunit 2 |
| Cops7b | COP9 signalosome subunit 7B |
| Cops8 | COP9 signalosome subunit 8 |
| Corin | corin |
| Coro1c | coronin |
| Cox10 | cytochrome c oxidase assembly protein 10 |
| Cox7a2l | cytochrome c oxidase subunit VIIa polypeptide 2-like |
| Cox7c | cytochrome c oxidase subunit VIIc |
| Cp | ceruloplasmin |
| Cpa6 | carboxypeptidase A6 |
| Cpb1 | carboxypeptidase B1 (tissue) |
| Cpd | carboxypeptidase D |
| Cpe | carboxypeptidase E |
| Cpeb1 | cytoplasmic polyadenylation element binding protein 1 |
| Cpeb4 | cytoplasmic polyadenylation element binding protein 4 |
| Cplx2 | complexin 2 |
| Cpne5 | copine V |
| Cpxm2 | carboxypeptidase X 2 (M14 family) |
| Crb1 | crumbs family member 1 |
| Crb2 | crumbs family member 2 |
| Creb1 | cAMP responsive element binding protein 1 |
| Creb3l2 | cAMP responsive element binding protein 3-like 2 |
| Creg2 | cellular repressor of E1A-stimulated genes 2 |
| Crhbp | corticotropin releasing hormone binding protein |
| Crhr1 | corticotropin releasing hormone receptor 1 |
| Crim1 | cysteine rich transmembrane BMP regulator 1 (chordin like) |
| Crisp4 | cysteine-rich secretory protein 4 |
| Crnde | colorectal neoplasia differentially expressed (non-protein coding) |
| Crtam | cytotoxic and regulatory T cell molecule |
| Crtc3 | CREB regulated transcription coactivator 3 |
| Csgalnact1 | chondroitin sulfate N-acetylgalactosaminyltransferase 1 |
| Csnk1d | casein kinase 1 |
| Csnk1g1 | casein kinase 1 |
| Csnk1g3 | casein kinase 1 |
| Csrp2bp | cysteine and glycine-rich protein 2 binding protein |
| Cst10 | cystatin 10 (chondrocytes) |
| Cstad | CSA-conditional |
| Cstf1 | cleavage stimulation factor |
| Ctgf | connective tissue growth factor |
| Ctla4 | cytotoxic T-lymphocyte-associated protein 4 |
| Ctnna3 | catenin (cadherin associated protein) |
| Ctnnal1 | catenin (cadherin associated protein) |
| Ctnnd1 | catenin (cadherin associated protein) |
| Ctnnd2 | catenin (cadherin associated protein) |
| Ctsc | cathepsin C |
| Ctss | cathepsin S |
| Cubn | cubilin (intrinsic factor-cobalamin receptor) |
| Cuedc1 | CUE domain containing 1 |
| Cul1 | cullin 1 |
| Cux1 | cut-like homeobox 1 |
| Cwc22 | CWC22 spliceosome-associated protein |
| Cwh43 | cell wall biogenesis 43 C-terminal homolog |
| Cxcl16 | chemokine (C-X-C motif) ligand 16 |
| Cxcl5 | chemokine (C-X-C motif) ligand 5 |
| Cxcr4 | chemokine (C-X-C motif) receptor 4 |
| Cxcr5 | chemokine (C-X-C motif) receptor 5 |
| Cxx1c | CAAX box 1C |
| Cxxc1 | CXXC finger 1 (PHD domain) |
| Cxxc5 | CXXC finger 5 |
| Cyb5b | cytochrome b5 type B |
| Cyp1b1 | cytochrome P450 |
| Cyp4f39 | cytochrome P450 |
| Cyth3 | cytohesin 3 |
| D030024E09Rik | RIKEN cDNA D030024E09 gene |
| D030025E07Rik | RIKEN cDNA D030025E07 gene |
| D10Jhu81e | DNA segment |
| D130043K22Rik | RIKEN cDNA D130043K22 gene |
| D14Ertd670e | DNA segment |
| D16Ertd472e | DNA segment |
| D17Wsu92e | DNA segment |
| D1Ertd622e | DNA segment |
| D330046F09Rik | RIKEN cDNA D330046F09 gene |
| D430041D05Rik | RIKEN cDNA D430041D05 gene |
| D430042O09Rik | RIKEN cDNA D430042O09 gene |
| D630003M21Rik | RIKEN cDNA D630003M21 gene |
| D630010B17Rik | RIKEN cDNA D630010B17 gene |
| D630024D03Rik | RIKEN cDNA D630024D03 gene |
| D630045J12Rik | RIKEN cDNA D630045J12 gene |
| D6Ertd474e | DNA segment |
| D6Ertd527e | DNA segment |
| D730001G18Rik | RIKEN cDNA D730001G18 gene |
| D830013O20Rik | RIKEN cDNA D830013O20 gene |
| D930015E06Rik | RIKEN cDNA D930015E06 gene |
| D930020B18Rik | RIKEN cDNA D930020B18 gene |
| Daam1 | dishevelled associated activator of morphogenesis 1 |
| Dab1 | disabled 1 |
| Dab2 | disabled 2 |
| Dact3 | dishevelled-binding antagonist of beta-catenin 3 |
| Dad1 | defender against cell death 1 |
| Dapk1 | death associated protein kinase 1 |
| Dapk2 | death-associated protein kinase 2 |
| Dapl1 | death associated protein-like 1 |
| Dapp1 | dual adaptor for phosphotyrosine and 3-phosphoinositides 1 |
| Dazap1 | DAZ associated protein 1 |
| Dcaf12 | DDB1 and CUL4 associated factor 12 |
| Dchs1 | dachsous 1 (Drosophila) |
| Dclk1 | doublecortin-like kinase 1 |
| Dctn1 | dynactin 1 |
| Dctn6 | dynactin 6 |
| Ddx10 | DEAD (Asp-Glu-Ala-Asp) box polypeptide 10 |
| Ddx3y | DEAD (Asp-Glu-Ala-Asp) box polypeptide 3 |
| Ddx4 | DEAD (Asp-Glu-Ala-Asp) box polypeptide 4 |
| Ddx41 | DEAD (Asp-Glu-Ala-Asp) box polypeptide 41 |
| Ddx60 | DEAD (Asp-Glu-Ala-Asp) box polypeptide 60 |
| Decr1 | 2 |
| Decr2 | 2-4-dienoyl-Coenzyme A reductase 2 |
| Dennd1a | DENN/MADD domain containing 1A |
| Dennd2a | DENN/MADD domain containing 2A |
| Dennd3 | DENN/MADD domain containing 3 |
| Depdc5 | DEP domain containing 5 |
| Dera | deoxyribose-phosphate aldolase (putative) |
| Derl3 | Der1-like domain family |
| Deup1 | deuterosome assembly protein 1 |
| Dgcr2 | DiGeorge syndrome critical region gene 2 |
| Dgka | diacylglycerol kinase |
| Dgkb | diacylglycerol kinase |
| Dgkg | diacylglycerol kinase |
| Dgkh | diacylglycerol kinase |
| Dgki | diacylglycerol kinase |
| Dgkz | diacylglycerol kinase zeta |
| Dhrs7 | dehydrogenase/reductase (SDR family) member 7 |
| Dhx29 | DEAH (Asp-Glu-Ala-His) box polypeptide 29 |
| Dhx32 | DEAH (Asp-Glu-Ala-His) box polypeptide 32 |
| Dhx34 | DEAH (Asp-Glu-Ala-His) box polypeptide 34 |
| Dhx35 | DEAH (Asp-Glu-Ala-His) box polypeptide 35 |
| Diaph2 | diaphanous related formin 2 |
| Diaph3 | diaphanous related formin 3 |
| Dio3 | deiodinase |
| Dio3os | deiodinase |
| Dip2b | disco interacting protein 2 homolog B |
| Dip2c | disco interacting protein 2 homolog C |
| Dis3l2 | DIS3 like 3'-5' exoribonuclease 2 |
| Disp1 | dispatched RND transporter family member 1 |
| Dleu2 | deleted in lymphocytic leukemia |
| Dleu7 | deleted in lymphocytic leukemia |
| Dlg1 | discs |
| Dlg3 | discs |
| Dlg5 | discs |
| Dlgap1 | discs |
| Dlgap4 | discs |
| Dmbt1 | deleted in malignant brain tumors 1 |
| Dmrt3 | doublesex and mab-3 related transcription factor 3 |
| Dmrta1 | doublesex and mab-3 related transcription factor like family A1 |
| Dmrtb1 | DMRT-like family B with proline-rich C-terminal |
| Dnaaf5 | dynein |
| Dnah17 | dynein |
| Dnah6 | dynein |
| Dnah7c | dynein |
| Dnaja2 | DnaJ heat shock protein family (Hsp40) member A2 |
| Dnajb4 | DnaJ heat shock protein family (Hsp40) member B4 |
| Dnajc10 | DnaJ heat shock protein family (Hsp40) member C10 |
| Dnajc24 | DnaJ heat shock protein family (Hsp40) member C24 |
| Dnajc3 | DnaJ heat shock protein family (Hsp40) member C3 |
| Dnajc6 | DnaJ heat shock protein family (Hsp40) member C6 |
| Dnase1l3 | deoxyribonuclease 1-like 3 |
| Dnd1 | DND microRNA-mediated repression inhibitor 1 |
| Dnm1 | dynamin 1 |
| Dnm2 | dynamin 2 |
| Dnm3 | dynamin 3 |
| Dnmbp | dynamin binding protein |
| Dnmt3a | DNA methyltransferase 3A |
| Dnmt3l | DNA (cytosine-5-)-methyltransferase 3-like |
| Dntt | deoxynucleotidyltransferase |
| Dock1 | dedicator of cytokinesis 1 |
| Dock10 | dedicator of cytokinesis 10 |
| Dock4 | dedicator of cytokinesis 4 |
| Dock9 | dedicator of cytokinesis 9 |
| Dok5 | docking protein 5 |
| Dok6 | docking protein 6 |
| Dpf1 | D4 |
| Dpf3 | D4 |
| Dph6 | diphthamine biosynthesis 6 |
| Dpp6 | dipeptidylpeptidase 6 |
| Dpp8 | dipeptidylpeptidase 8 |
| Dppa3 | developmental pluripotency-associated 3 |
| Dpy19l1 | dpy-19-like 1 (C. elegans) |
| Dpy19l2 | dpy-19-like 2 (C. elegans) |
| Dpy19l3 | dpy-19-like 3 (C. elegans) |
| Drp2 | dystrophin related protein 2 |
| Dsc3 | desmocollin 3 |
| Dst | dystonin |
| Dtd1 | D-tyrosyl-tRNA deacylase 1 |
| Dtl | denticleless E3 ubiquitin protein ligase |
| Dtnb | dystrobrevin |
| Dtwd2 | DTW domain containing 2 |
| Dusp10 | dual specificity phosphatase 10 |
| Dusp12 | dual specificity phosphatase 12 |
| Dusp14 | dual specificity phosphatase 14 |
| Dusp15 | dual specificity phosphatase-like 15 |
| Dusp16 | dual specificity phosphatase 16 |
| Dusp22 | dual specificity phosphatase 22 |
| Dusp6 | dual specificity phosphatase 6 |
| Dusp8 | dual specificity phosphatase 8 |
| Dux | double homeobox |
| Dync1i1 | dynein cytoplasmic 1 intermediate chain 1 |
| Dync1li2 | dynein |
| Dync2h1 | dynein cytoplasmic 2 heavy chain 1 |
| Dync2li1 | dynein cytoplasmic 2 light intermediate chain 1 |
| Dynlrb2 | dynein light chain roadblock-type 2 |
| Dyrk2 | dual-specificity tyrosine-(Y)-phosphorylation regulated kinase 2 |
| Dyrk3 | dual-specificity tyrosine-(Y)-phosphorylation regulated kinase 3 |
| Dyrk4 | dual-specificity tyrosine-(Y)-phosphorylation regulated kinase 4 |
| E130006D01Rik | RIKEN cDNA E130006D01 gene |
| E130114P18Rik | RIKEN cDNA E130114P18 gene |
| E230016M11Rik | RIKEN cDNA E230016M11 gene |
| E2f3 | E2F transcription factor 3 |
| E330011O21Rik | RIKEN cDNA E330011O21 gene |
| E330017L17Rik | RIKEN cDNA E330017L17 gene |
| E330021D16Rik | RIKEN cDNA E330021D16 gene |
| E430016F16Rik | RIKEN cDNA E430016F16 gene |
| Eci2 | enoyl-Coenzyme A delta isomerase 2 |
| Eci3 | enoyl-Coenzyme A delta isomerase 3 |
| Edil3 | EGF-like repeats and discoidin I-like domains 3 |
| Edn2 | endothelin 2 |
| Edn3 | endothelin 3 |
| Ednrb | endothelin receptor type B |
| Edrf1 | erythroid differentiation regulatory factor 1 |
| Eea1 | early endosome antigen 1 |
| Eef2k | eukaryotic elongation factor-2 kinase |
| Eef2kmt | eukaryotic elongation factor 2 lysine methyltransferase |
| Eefsec | eukaryotic elongation factor |
| Eepd1 | endonuclease/exonuclease/phosphatase family domain containing 1 |
| Efcab11 | EF-hand calcium binding domain 11 |
| Efcab3 | EF-hand calcium binding domain 3 |
| Efcc1 | EF hand and coiled-coil domain containing 1 |
| Efhc1 | EF-hand domain (C-terminal) containing 1 |
| Efna5 | ephrin A5 |
| Efr3b | EFR3 homolog B |
| Egfl7 | EGF-like domain 7 |
| Egfr | epidermal growth factor receptor |
| Egln3 | egl-9 family hypoxia-inducible factor 3 |
| Egr4 | early growth response 4 |
| Ehbp1 | EH domain binding protein 1 |
| Ehf | ets homologous factor |
| Ehmt1 | euchromatic histone methyltransferase 1 |
| Ei24 | etoposide induced 2.4 mRNA |
| Eif2ak3 | eukaryotic translation initiation factor 2 alpha kinase 3 |
| Eif2b2 | eukaryotic translation initiation factor 2B |
| Eif3h | eukaryotic translation initiation factor 3 |
| Eif3m | eukaryotic translation initiation factor 3 |
| Eif4a2 | eukaryotic translation initiation factor 4A2 |
| Eif4e3 | eukaryotic translation initiation factor 4E member 3 |
| Elac2 | elaC ribonuclease Z 2 |
| Elavl1 | ELAV (embryonic lethal |
| Elavl2 | ELAV (embryonic lethal |
| Elfn1 | leucine rich repeat and fibronectin type III |
| Elfn2 | leucine rich repeat and fibronectin type III |
| Elmo1 | engulfment and cell motility 1 |
| Elovl5 | ELOVL family member 5 |
| Elovl6 | ELOVL family member 6 |
| Emb | embigin |
| Emilin3 | elastin microfibril interfacer 3 |
| Eml4 | echinoderm microtubule associated protein like 4 |
| Emp1 | epithelial membrane protein 1 |
| Emx1 | empty spiracles homeobox 1 |
| En2 | engrailed 2 |
| Enc1 | ectodermal-neural cortex 1 |
| Endod1 | endonuclease domain containing 1 |
| Enox1 | ecto-NOX disulfide-thiol exchanger 1 |
| Enpp1 | ectonucleotide pyrophosphatase/phosphodiesterase 1 |
| Enpp3 | ectonucleotide pyrophosphatase/phosphodiesterase 3 |
| Enpp6 | ectonucleotide pyrophosphatase/phosphodiesterase 6 |
| Enthd1 | ENTH domain containing 1 |
| Entpd7 | ectonucleoside triphosphate diphosphohydrolase 7 |
| Epas1 | endothelial PAS domain protein 1 |
| Epb41l2 | erythrocyte membrane protein band 4.1 like 2 |
| Epb41l4aos | erythrocyte membrane protein band 4.1 like 4a |
| Epb41l4b | erythrocyte membrane protein band 4.1 like 4b |
| Epb41l5 | erythrocyte membrane protein band 4.1 like 5 |
| Epc2 | enhancer of polycomb homolog 2 (Drosophila) |
| Epcam | epithelial cell adhesion molecule |
| Ephb2 | Eph receptor B2 |
| Epn2 | epsin 2 |
| Eps15 | epidermal growth factor receptor pathway substrate 15 |
| Erbb4 | erb-b2 receptor tyrosine kinase 4 |
| Erc1 | ELKS/RAB6-interacting/CAST family member 1 |
| Ercc3 | excision repair cross-complementing rodent repair deficiency |
| Erg | avian erythroblastosis virus E-26 (v-ets) oncogene related |
| Ermn | ermin |
| Esm1 | endothelial cell-specific molecule 1 |
| Esrp1 | epithelial splicing regulatory protein 1 |
| Etnk1 | ethanolamine kinase 1 |
| Ets1 | E26 avian leukemia oncogene 1 |
| Etv3 | ets variant 3 |
| Etv6 | ets variant 6 |
| Evx1os | even skipped homeotic gene 1 |
| Exoc2 | exocyst complex component 2 |
| Exoc4 | exocyst complex component 4 |
| Ext1 | exostoses (multiple) 1 |
| Extl1 | exostoses (multiple)-like 1 |
| Extl3 | exostoses (multiple)-like 3 |
| Eya1 | EYA transcriptional coactivator and phosphatase 1 |
| Eya3 | EYA transcriptional coactivator and phosphatase 3 |
| Ezh1 | enhancer of zeste 1 polycomb repressive complex 2 subunit |
| F13a1 | coagulation factor XIII |
| F2rl1 | coagulation factor II (thrombin) receptor-like 1 |
| Faap20 | Fanconi anemia core complex associated protein 20 |
| Fabp12 | fatty acid binding protein 12 |
| Fabp3 | fatty acid binding protein 3 |
| Fabp4 | fatty acid binding protein 4 |
| Fads3 | fatty acid desaturase 3 |
| Faf1 | Fas-associated factor 1 |
| Fam102a | family with sequence similarity 102 |
| Fam117b | family with sequence similarity 117 |
| Fam124b | family with sequence similarity 124 |
| Fam134b | family with sequence similarity 134 |
| Fam135a | family with sequence similarity 135 |
| Fam136a | family with sequence similarity 136 |
| Fam13a | family with sequence similarity 13 |
| Fam155a | family with sequence similarity 155 |
| Fam159b | family with sequence similarity 159 |
| Fam163a | family with sequence similarity 163 |
| Fam163b | family with sequence similarity 163 |
| Fam167a | family with sequence similarity 167 |
| Fam168a | family with sequence similarity 168 |
| Fam168b | family with sequence similarity 168 |
| Fam171b | family with sequence similarity 171 |
| Fam172a | family with sequence similarity 172 |
| Fam173b | family with sequence similarity 173 |
| Fam174a | family with sequence similarity 174 |
| Fam174b | family with sequence similarity 174 |
| Fam175b | family with sequence similarity 175 |
| Fam184a | family with sequence similarity 184 |
| Fam188b | family with sequence similarity 188 |
| Fam193a | family with sequence similarity 193 |
| Fam19a2 | family with sequence similarity 19 |
| Fam20c | family with sequence similarity 20 |
| Fam214a | family with sequence similarity 214 |
| Fam217a | family with sequence similarity 217 |
| Fam220a | family with sequence similarity 220 |
| Fam221a | family with sequence similarity 221 |
| Fam234a | family with sequence similarity 234 |
| Fam3c | family with sequence similarity 3 |
| Fam43a | family with sequence similarity 43 |
| Fam43b | family with sequence similarity 43 |
| Fam46a | family with sequence similarity 46 |
| Fam46c | family with sequence similarity 46 |
| Fam49a | family with sequence similarity 49 |
| Fam49b | family with sequence similarity 49 |
| Fam53b | family with sequence similarity 53 |
| Fam60a | family with sequence similarity 60 |
| Fam69a | family with sequence similarity 69 |
| Fam69c | family with sequence similarity 69 |
| Fam71b | family with sequence similarity 71 |
| Fam76b | family with sequence similarity 76 |
| Fam78a | family with sequence similarity 78 |
| Fam81a | family with sequence similarity 81 |
| Fam83b | family with sequence similarity 83 |
| Fam83g | family with sequence similarity 83 |
| Fam84a | family with sequence similarity 84 |
| Fancc | Fanconi anemia |
| Fanci | Fanconi anemia |
| Fancl | Fanconi anemia |
| Farp1 | FERM |
| Farp2 | FERM |
| Fars2 | phenylalanine-tRNA synthetase 2 (mitochondrial) |
| Fasl | Fas ligand (TNF superfamily |
| Fat4 | FAT atypical cadherin 4 |
| Fbln1 | fibulin 1 |
| Fbn1 | fibrillin 1 |
| Fbxl12 | F-box and leucine-rich repeat protein 12 |
| Fbxl17 | F-box and leucine-rich repeat protein 17 |
| Fbxl21 | F-box and leucine-rich repeat protein 21 |
| Fbxo11 | F-box protein 11 |
| Fbxo15 | F-box protein 15 |
| Fbxo30 | F-box protein 30 |
| Fbxo32 | F-box protein 32 |
| Fbxo36 | F-box protein 36 |
| Fbxo41 | F-box protein 41 |
| Fbxo45 | F-box protein 45 |
| Fbxo9 | f-box protein 9 |
| Fbxw11 | F-box and WD-40 domain protein 11 |
| Fcgr3 | Fc receptor |
| Fcmr | Fc fragment of IgM receptor |
| Fcrl1 | Fc receptor-like 1 |
| Fendrr | Foxf1 adjacent non-coding developmental regulatory RNA |
| Fer | fer (fms/fps related) protein kinase |
| Fermt2 | fermitin family member 2 |
| Fez2 | fasciculation and elongation protein zeta 2 (zygin II) |
| Fgd4 | FYVE |
| Fgd6 | FYVE |
| Fgf12 | fibroblast growth factor 12 |
| Fgf14 | fibroblast growth factor 14 |
| Fgf2 | fibroblast growth factor 2 |
| Fgf20 | fibroblast growth factor 20 |
| Fgf8 | fibroblast growth factor 8 |
| Fgfr1op | Fgfr1 oncogene partner |
| Fggy | FGGY carbohydrate kinase domain containing |
| Fhit | fragile histidine triad gene |
| Fhl4 | four and a half LIM domains 4 |
| Fhod3 | formin homology 2 domain containing 3 |
| Fibcd1 | fibrinogen C domain containing 1 |
| Fignl2 | fidgetin-like 2 |
| Filip1 | filamin A interacting protein 1 |
| Filip1l | filamin A interacting protein 1-like |
| Fjx1 | four jointed box 1 (Drosophila) |
| Fli1 | Friend leukemia integration 1 |
| Flnb | filamin |
| Flt3 | FMS-like tyrosine kinase 3 |
| Flt4 | FMS-like tyrosine kinase 4 |
| Fmnl2 | formin-like 2 |
| Fn1 | fibronectin 1 |
| Fnbp1 | formin binding protein 1 |
| Fnbp4 | formin binding protein 4 |
| Fndc7 | fibronectin type III domain containing 7 |
| Fntb | farnesyltransferase |
| Focad | focadhesin |
| Fopnl | Fgfr1op N-terminal like |
| Foxb2 | forkhead box B2 |
| Foxc1 | forkhead box C1 |
| Foxi2 | forkhead box I2 |
| Foxk1 | forkhead box K1 |
| Foxn2 | forkhead box N2 |
| Foxn3 | forkhead box N3 |
| Foxo1 | forkhead box O1 |
| Foxo3 | forkhead box O3 |
| Foxo6 | forkhead box O6 |
| Foxp1 | forkhead box P1 |
| Foxq1 | forkhead box Q1 |
| Frmd4a | FERM domain containing 4A |
| Frmd4b | FERM domain containing 4B |
| Frmd5 | FERM domain containing 5 |
| Frmd6 | FERM domain containing 6 |
| Frmd7 | FERM domain containing 7 |
| Frmpd1 | FERM and PDZ domain containing 1 |
| Frmpd4 | FERM and PDZ domain containing 4 |
| Frrs1 | ferric-chelate reductase 1 |
| Frs3os | fibroblast growth factor receptor substrate 3 |
| Fry | FRY microtubule binding protein |
| Fryl | FRY like transcription coactivator |
| Frzb | frizzled-related protein |
| Fscn1 | fascin actin-bundling protein 1 |
| Fsd1l | fibronectin type III and SPRY domain containing 1-like |
| Fsd2 | fibronectin type III and SPRY domain containing 2 |
| Fstl1 | follistatin-like 1 |
| Fstl4 | follistatin-like 4 |
| Fstl5 | follistatin-like 5 |
| Fut10 | fucosyltransferase 10 |
| Fut8 | fucosyltransferase 8 |
| Fxyd7 | FXYD domain-containing ion transport regulator 7 |
| Fyb | FYN binding protein |
| Fzd10 | frizzled class receptor 10 |
| G730013B05Rik | RIKEN cDNA G730013B05 gene |
| Gab1 | growth factor receptor bound protein 2-associated protein 1 |
| Gabbr2 | gamma-aminobutyric acid (GABA) B receptor |
| Gabpb2 | GA repeat binding protein |
| Gabrb1 | gamma-aminobutyric acid (GABA) A receptor |
| Gabrr1 | gamma-aminobutyric acid (GABA) C receptor |
| Gabrr2 | gamma-aminobutyric acid (GABA) C receptor |
| Gad2 | glutamic acid decarboxylase 2 |
| Gadd45a | growth arrest and DNA-damage-inducible 45 alpha |
| Gadl1 | glutamate decarboxylase-like 1 |
| Gal3st1 | galactose-3-O-sulfotransferase 1 |
| Galc | galactosylceramidase |
| Galm | galactose mutarotase |
| Galnt1 | UDP-N-acetyl-alpha-D-galactosamine:polypeptide N-acetylgalactosaminyltransferase 1 |
| Galnt18 | UDP-N-acetyl-alpha-D-galactosamine:polypeptide N-acetylgalactosaminyltransferase 18 |
| Galnt5 | UDP-N-acetyl-alpha-D-galactosamine:polypeptide N-acetylgalactosaminyltransferase 5 |
| Galnt7 | UDP-N-acetyl-alpha-D-galactosamine: polypeptide N-acetylgalactosaminyltransferase 7 |
| Galr1 | galanin receptor 1 |
| Garem1 | GRB2 associated regulator of MAPK1 subtype 1 |
| Gars | glycyl-tRNA synthetase |
| Gas2l3 | growth arrest-specific 2 like 3 |
| Gata3 | GATA binding protein 3 |
| Gata4 | GATA binding protein 4 |
| Gatm | glycine amidinotransferase (L-arginine:glycine amidinotransferase) |
| Gatsl2 | GATS protein-like 2 |
| Gbp7 | guanylate binding protein 7 |
| Gch1 | GTP cyclohydrolase 1 |
| Gcm1 | glial cells missing homolog 1 (Drosophila) |
| Gcnt1 | glucosaminyl (N-acetyl) transferase 1 |
| Gda | guanine deaminase |
| Gdf6 | growth differentiation factor 6 |
| Gdpd5 | glycerophosphodiester phosphodiesterase domain containing 5 |
| Gemin5 | gem (nuclear organelle) associated protein 5 |
| Gemin8 | gem (nuclear organelle) associated protein 8 |
| Gfpt2 | glutamine fructose-6-phosphate transaminase 2 |
| Gfra1 | glial cell line derived neurotrophic factor family receptor alpha 1 |
| Gfra4 | glial cell line derived neurotrophic factor family receptor alpha 4 |
| Gga3 | golgi associated |
| Ggta1 | glycoprotein galactosyltransferase alpha 1 |
| Gigyf2 | GRB10 interacting GYF protein 2 |
| Gip | gastric inhibitory polypeptide |
| Gipc1 | GIPC PDZ domain containing family |
| Gipc2 | GIPC PDZ domain containing family |
| Git2 | G protein-coupled receptor kinase-interactor 2 |
| Gja1 | gap junction protein |
| Gja3 | gap junction protein |
| Gja5 | gap junction protein |
| Gk | glycerol kinase |
| Gk2 | glycerol kinase 2 |
| Glb1 | galactosidase |
| Glcci1 | glucocorticoid induced transcript 1 |
| Glce | glucuronyl C5-epimerase |
| Gli2 | GLI-Kruppel family member GLI2 |
| Glipr1 | GLI pathogenesis-related 1 (glioma) |
| Glis3 | GLIS family zinc finger 3 |
| Glra1 | glycine receptor |
| Glrb | glycine receptor |
| Glrp1 | glutamine repeat protein 1 |
| Gltp | glycolipid transfer protein |
| Gm10354 | predicted gene 10354 |
| Gm10790 | predicted gene 10790 |
| Gm10863 | predicted gene 10863 |
| Gm11240 | predicted gene 11240 |
| Gm11627 | predicted gene 11627 |
| Gm11696 | predicted gene 11696 |
| Gm12108 | predicted gene 12108 |
| Gm12169 | predicted gene 12169 |
| Gm12409 | predicted gene 12409 |
| Gm12596 | predicted gene 12596 |
| Gm12610 | predicted gene 12610 |
| Gm13483 | predicted gene 13483 |
| Gm13498 | heat shock protein 90 alpha (cytosolic) |
| Gm13546 | predicted gene 13546 |
| Gm13710 | predicted gene 13710 |
| Gm13889 | predicted gene 13889 |
| Gm13986 | predicted gene 13986 |
| Gm14005 | predicted gene 14005 |
| Gm15446 | predicted gene 15446 |
| Gm15972 | predicted gene 15972 |
| Gm16063 | predicted gene 16063 |
| Gm16432 | predicted gene 16432 |
| Gm1647 | predicted gene 1647 |
| Gm1653 | predicted gene 1653 |
| Gm16675 | predicted gene |
| Gm1673 | predicted gene 1673 |
| Gm16863 | predicted gene |
| Gm1720 | predicted gene 1720 |
| Gm17359 | predicted gene |
| Gm17746 | predicted gene |
| Gm19466 | predicted gene |
| Gm19510 | predicted gene |
| Gm19589 | predicted gene |
| Gm19619 | predicted gene |
| Gm1966 | predicted gene 1966 |
| Gm1968 | predicted gene 1968 |
| Gm20098 | predicted gene |
| Gm20110 | predicted gene |
| Gm20125 | predicted gene |
| Gm20139 | predicted gene |
| Gm20743 | predicted gene |
| Gm20754 | predicted gene |
| Gm20755 | predicted gene |
| Gm2087 | predicted gene 2087 |
| Gm2115 | predicted gene 2115 |
| Gm21221 | predicted gene |
| Gm2447 | predicted gene 2447 |
| Gm26688 | predicted gene |
| Gm27162 | predicted gene 27162 |
| Gm29682 | predicted gene |
| Gm29685 | predicted gene |
| Gm29687 | predicted gene |
| Gm31763 | predicted gene |
| Gm3230 | predicted gene 3230 |
| Gm3704 | predicted gene 3704 |
| Gm3776 | predicted gene 3776 |
| Gm38403 | predicted gene |
| Gm38404 | predicted gene |
| Gm38437 | predicted gene |
| Gm4251 | predicted gene 4251 |
| Gm4489 | predicted gene 4489 |
| Gm45924 | predicted gene |
| Gm4737 | predicted gene 4737 |
| Gm4832 | predicted gene 4832 |
| Gm4841 | predicted gene 4841 |
| Gm4971 | Smad nuclear interacting protein 1 pseudogene |
| Gm5065 | predicted gene 5065 |
| Gm5082 | predicted gene 5082 |
| Gm5083 | predicted gene 5083 |
| Gm5086 | predicted gene 5086 |
| Gm5105 | predicted gene 5105 |
| Gm5122 | predicted gene 5122 |
| Gm5126 | predicted pseudogene 5126 |
| Gm5127 | predicted gene 5127 |
| Gm5144 | predicted gene 5144 |
| Gm5468 | predicted gene 5468 |
| Gm5538 | predicted gene 5538 |
| Gm5544 | predicted gene 5544 |
| Gm5607 | predicted gene 5607 |
| Gm572 | predicted gene 572 |
| Gm5833 | predicted gene 5833 |
| Gm5893 | predicted gene 5893 |
| Gm590 | predicted gene 590 |
| Gm6260 | predicted gene 6260 |
| Gm6416 | predicted gene 6416 |
| Gm6498 | glyceraldehyde-3-phosphate dehydrogenase pseudogene |
| Gm6559 | predicted gene 6559 |
| Gm6639 | predicted gene 6639 |
| Gm684 | predicted gene 684 |
| Gm6936 | predicted gene 6936 |
| Gm6961 | predicted gene 6961 |
| Gm7244 | predicted gene 7244 |
| Gm7538 | predicted gene 7538 |
| Gm765 | predicted gene 765 |
| Gm8013 | predicted gene 8013 |
| Gm829 | predicted gene 829 |
| Gm833 | predicted gene 833 |
| Gm8709 | glyceraldehyde-3-phosphate dehydrogenase pseudogene |
| Gm8817 | predicted gene 8817 |
| Gm8884 | predicted gene 8884 |
| Gm9199 | glycine cleavage system protein H (aminomethyl carrier) pseudogene |
| Gm973 | predicted gene 973 |
| Gm9776 | predicted gene 9776 |
| Gm9926 | predicted gene 9926 |
| Gm9992 | predicted gene 9992 |
| Gmpr | guanosine monophosphate reductase |
| Gnai3 | guanine nucleotide binding protein (G protein) |
| Gnal | guanine nucleotide binding protein |
| Gnaq | guanine nucleotide binding protein |
| Gnb4 | guanine nucleotide binding protein (G protein) |
| Gng12 | guanine nucleotide binding protein (G protein) |
| Gnpda1 | glucosamine-6-phosphate deaminase 1 |
| Golph3 | golgi phosphoprotein 3 |
| Gpatch2 | G patch domain containing 2 |
| Gpbp1 | GC-rich promoter binding protein 1 |
| Gpc1 | glypican 1 |
| Gpc3 | glypican 3 |
| Gpd2 | glycerol phosphate dehydrogenase 2 |
| Gphn | gephyrin |
| Gpm6b | glycoprotein m6b |
| Gpnmb | glycoprotein (transmembrane) nmb |
| Gpr12 | G-protein coupled receptor 12 |
| Gpr15 | G protein-coupled receptor 15 |
| Gpr151 | G protein-coupled receptor 151 |
| Gpr158 | G protein-coupled receptor 158 |
| Gpr160 | G protein-coupled receptor 160 |
| Gpr180 | G protein-coupled receptor 180 |
| Gpr19 | G protein-coupled receptor 19 |
| Gpr33 | G protein-coupled receptor 33 |
| Gpr37 | G protein-coupled receptor 37 |
| Gpr37l1 | G protein-coupled receptor 37-like 1 |
| Gpr50 | G-protein-coupled receptor 50 |
| Gpr55 | G protein-coupled receptor 55 |
| Gpr65 | G-protein coupled receptor 65 |
| Gprasp2 | G protein-coupled receptor associated sorting protein 2 |
| Gprc5b | G protein-coupled receptor |
| Gprc5c | G protein-coupled receptor |
| Gprin3 | GPRIN family member 3 |
| Gramd1b | GRAM domain containing 1B |
| Gramd3 | GRAM domain containing 3 |
| Grap2 | GRB2-related adaptor protein 2 |
| Grb10 | growth factor receptor bound protein 10 |
| Grb14 | growth factor receptor bound protein 14 |
| Grb2 | growth factor receptor bound protein 2 |
| Grhl1 | grainyhead-like 1 (Drosophila) |
| Grhl2 | grainyhead-like 2 (Drosophila) |
| Gria3 | glutamate receptor |
| Grid2 | glutamate receptor |
| Grik1 | glutamate receptor |
| Grik4 | glutamate receptor |
| Grin2b | glutamate receptor |
| Grip1 | glutamate receptor interacting protein 1 |
| Grm4 | glutamate receptor |
| Grm6 | glutamate receptor |
| Grpr | gastrin releasing peptide receptor |
| Gsap | gamma-secretase activating protein |
| Gsc | goosecoid homeobox |
| Gse1 | genetic suppressor element 1 |
| Gsk3b | glycogen synthase kinase 3 beta |
| Gstt2 | glutathione S-transferase |
| Gstt4 | glutathione S-transferase |
| Gsx2 | GS homeobox 2 |
| Gtdc1 | glycosyltransferase-like domain containing 1 |
| Gtf2b | general transcription factor IIB |
| Gtf2ird1 | general transcription factor II I repeat domain-containing 1 |
| Gtf3c3 | general transcription factor IIIC |
| Gtpbp4 | GTP binding protein 4 |
| Guca2b | guanylate cyclase activator 2b (retina) |
| Gucy1a3 | guanylate cyclase 1 |
| Gucy1b3 | guanylate cyclase 1 |
| Gulp1 | GULP |
| Gvin1 | GTPase |
| Gxylt2 | glucoside xylosyltransferase 2 |
| Gypa | glycophorin A |
| Gypc | glycophorin C |
| H2afy3 | H2A histone family |
| H2bfm | H2B histone family |
| H6pd | hexose-6-phosphate dehydrogenase (glucose 1-dehydrogenase) |
| Hacd3 | 3-hydroxyacyl-CoA dehydratase 3 |
| Halr1 | Hoxa adjacent long noncoding RNA 1 |
| Hapln1 | hyaluronan and proteoglycan link protein 1 |
| Hbs1l | Hbs1-like (S. cerevisiae) |
| Hcfc2 | host cell factor C2 |
| Hcn1 | hyperpolarization-activated |
| Hdac4 | histone deacetylase 4 |
| Hdac9 | histone deacetylase 9 |
| Hdx | highly divergent homeobox |
| Hecw1 | HECT |
| Hecw2 | HECT |
| Heg1 | heart development protein with EGF-like domains 1 |
| Helz | helicase with zinc finger domain |
| Hepacam | hepatocyte cell adhesion molecule |
| Hephl1 | hephaestin-like 1 |
| Herc1 | HECT and RLD domain containing E3 ubiquitin protein ligase family member 1 |
| Herpud2 | HERPUD family member 2 |
| Hexim2 | hexamethylene bis-acetamide inducible 2 |
| Heyl | hairy/enhancer-of-split related with YRPW motif-like |
| Hfm1 | HFM1 |
| Hgd | homogentisate 1 |
| Hhat | hedgehog acyltransferase |
| Hhipl1 | hedgehog interacting protein-like 1 |
| Hhipl2 | hedgehog interacting protein-like 2 |
| Hibch | 3-hydroxyisobutyryl-Coenzyme A hydrolase |
| Hilpda | hypoxia inducible lipid droplet associated |
| Hist1h4n | histone cluster 1 |
| Hivep3 | human immunodeficiency virus type I enhancer binding protein 3 |
| Hk1 | hexokinase 1 |
| Hlcs | holocarboxylase synthetase (biotin- [propriony-Coenzyme A-carboxylase (ATP-hydrolysing)] ligase) |
| Hlf | hepatic leukemia factor |
| Hmcn1 | hemicentin 1 |
| Hmgb1 | high mobility group box 1 |
| Hmgb1-rs17 | high mobility group box 1 |
| Hmgn1 | high mobility group nucleosomal binding domain 1 |
| Hmox2 | heme oxygenase 2 |
| Hnrnpa3 | heterogeneous nuclear ribonucleoprotein A3 |
| Hnrnpll | heterogeneous nuclear ribonucleoprotein L-like |
| Homer1 | homer scaffolding protein 1 |
| Hopx | HOP homeobox |
| Hormad2 | HORMA domain containing 2 |
| Hoxa1 | homeobox A1 |
| Hoxd1 | homeobox D1 |
| Hoxd11 | homeobox D11 |
| Hpca | hippocalcin |
| Hpcal1 | hippocalcin-like 1 |
| Hpn | hepsin |
| Hpse | heparanase |
| Hrh1 | histamine receptor H1 |
| Hs2st1 | heparan sulfate 2-O-sulfotransferase 1 |
| Hs3st1 | heparan sulfate (glucosamine) 3-O-sulfotransferase 1 |
| Hs3st3b1 | heparan sulfate (glucosamine) 3-O-sulfotransferase 3B1 |
| Hs6st1 | heparan sulfate 6-O-sulfotransferase 1 |
| Hsd17b10 | hydroxysteroid (17-beta) dehydrogenase 10 |
| Hsd17b4 | hydroxysteroid (17-beta) dehydrogenase 4 |
| Hsf2bp | heat shock transcription factor 2 binding protein |
| Hspa12a | heat shock protein 12A |
| Hspa13 | heat shock protein 70 family |
| Hspa4l | heat shock protein 4 like |
| Hspb11 | heat shock protein family B (small) |
| Hspb8 | heat shock protein 8 |
| Hspbap1 | Hspb associated protein 1 |
| Htr1f | 5-hydroxytryptamine (serotonin) receptor 1F |
| Htr4 | 5 hydroxytryptamine (serotonin) receptor 4 |
| Htr5b | 5-hydroxytryptamine (serotonin) receptor 5B |
| Htr7 | 5-hydroxytryptamine (serotonin) receptor 7 |
| Htt | huntingtin |
| Hunk | hormonally upregulated Neu-associated kinase |
| Hyal5 | hyaluronoglucosaminidase 5 |
| I730028E13Rik | RIKEN cDNA I730028E13 gene |
| I830077J02Rik | RIKEN cDNA I830077J02 gene |
| Ibtk | inhibitor of Bruton agammaglobulinemia tyrosine kinase |
| Icos | inducible T cell co-stimulator |
| Id3 | inhibitor of DNA binding 3 |
| Idh2 | isocitrate dehydrogenase 2 (NADP+) |
| Ido2 | indoleamine 2 |
| Ier5 | immediate early response 5 |
| Ier5l | immediate early response 5-like |
| Ifi214 | interferon activated gene 214 |
| Ifi44 | interferon-induced protein 44 |
| Ifit2 | interferon-induced protein with tetratricopeptide repeats 2 |
| Ifnar2 | interferon (alpha and beta) receptor 2 |
| Ifne | interferon epsilon |
| Ifng | interferon gamma |
| Ifngr2 | interferon gamma receptor 2 |
| Ift43 | intraflagellar transport 43 |
| Ift57 | intraflagellar transport 57 |
| Igf1 | insulin-like growth factor 1 |
| Igf1r | insulin-like growth factor I receptor |
| Igf2 | insulin-like growth factor 2 |
| Igf2bp1 | insulin-like growth factor 2 mRNA binding protein 1 |
| Igf2bp2 | insulin-like growth factor 2 mRNA binding protein 2 |
| Igfbp2 | insulin-like growth factor binding protein 2 |
| Igfbp3 | insulin-like growth factor binding protein 3 |
| Iigp1 | interferon inducible GTPase 1 |
| Ikzf2 | IKAROS family zinc finger 2 |
| Ikzf3 | IKAROS family zinc finger 3 |
| Il12rb2 | interleukin 12 receptor |
| Il17f | interleukin 17F |
| Il18 | interleukin 18 |
| Il18rap | interleukin 18 receptor accessory protein |
| Il1b | interleukin 1 beta |
| Il1f5 | interleukin 1 family |
| Il1f8 | interleukin 1 family |
| Il1r1 | interleukin 1 receptor |
| Il1r2 | interleukin 1 receptor |
| Il20 | interleukin 20 |
| Il20rb | interleukin 20 receptor beta |
| Il21 | interleukin 21 |
| Il22ra2 | interleukin 22 receptor |
| Il23a | interleukin 23 |
| Il23r | interleukin 23 receptor |
| Il27 | interleukin 27 |
| Il6 | interleukin 6 |
| Il6st | interleukin 6 signal transducer |
| Il7r | interleukin 7 receptor |
| Ildr2 | immunoglobulin-like domain containing receptor 2 |
| Iltifb | interleukin 10-related T cell-derived inducible factor beta |
| Immp2l | IMP2 inner mitochondrial membrane peptidase-like (S. cerevisiae) |
| Inf2 | inverted formin |
| Ing1 | inhibitor of growth family |
| Inhba | inhibin beta-A |
| Ino80c | INO80 complex subunit C |
| Ino80d | INO80 complex subunit D |
| Ino80dos | INO80 complex subunit D |
| Inpp4b | inositol polyphosphate-4-phosphatase |
| Insig2 | insulin induced gene 2 |
| Insr | insulin receptor |
| Ints13 | integrator complex subunit 13 |
| Ints3 | integrator complex subunit 3 |
| Ints6 | integrator complex subunit 6 |
| Ipcef1 | interaction protein for cytohesin exchange factors 1 |
| Ipo5 | importin 5 |
| Iqck | IQ motif containing K |
| Iqgap2 | IQ motif containing GTPase activating protein 2 |
| Irak1bp1 | interleukin-1 receptor-associated kinase 1 binding protein 1 |
| Ireb2 | iron responsive element binding protein 2 |
| Irf2bp2 | interferon regulatory factor 2 binding protein 2 |
| Irf4 | interferon regulatory factor 4 |
| Irf8 | interferon regulatory factor 8 |
| Irs4 | insulin receptor substrate 4 |
| Irx4 | Iroquois related homeobox 4 (Drosophila) |
| Irx5 | Iroquois related homeobox 5 (Drosophila) |
| Islr2 | immunoglobulin superfamily containing leucine-rich repeat 2 |
| Ism1 | isthmin 1 |
| Isoc2b | isochorismatase domain containing 2b |
| Ispd | isoprenoid synthase domain containing |
| Itga4 | integrin alpha 4 |
| Itga5 | integrin alpha 5 (fibronectin receptor alpha) |
| Itga9 | integrin alpha 9 |
| Itgb1 | integrin beta 1 (fibronectin receptor beta) |
| Itgb3 | integrin beta 3 |
| Itgb5 | integrin beta 5 |
| Itgb6 | integrin beta 6 |
| Itm2a | integral membrane protein 2A |
| Itpkb | inositol 1 |
| Itpr1 | inositol 1 |
| Itpr2 | inositol 1 |
| Itpr3 | inositol 1 |
| Itpripl2 | inositol 1 |
| Itsn2 | intersectin 2 |
| Iws1 | IWS1 |
| Jade2 | jade family PHD finger 2 |
| Jak1 | Janus kinase 1 |
| Jak2 | Janus kinase 2 |
| Jakmip1 | janus kinase and microtubule interacting protein 1 |
| Jakmip3 | janus kinase and microtubule interacting protein 3 |
| Jam2 | junction adhesion molecule 2 |
| Jam3 | junction adhesion molecule 3 |
| Jaml | junction adhesion molecule like |
| Jarid2 | jumonji |
| Jazf1 | JAZF zinc finger 1 |
| Jmy | junction-mediating and regulatory protein |
| Jun | jun proto-oncogene |
| Kank1 | KN motif and ankyrin repeat domains 1 |
| Kank4 | KN motif and ankyrin repeat domains 4 |
| Kat2b | K(lysine) acetyltransferase 2B |
| Kat6b | K(lysine) acetyltransferase 6B |
| Kazn | kazrin |
| Kcna5 | potassium voltage-gated channel |
| Kcnb1 | potassium voltage gated channel |
| Kcnb2 | potassium voltage gated channel |
| Kcnc2 | potassium voltage gated channel |
| Kcnd3 | potassium voltage-gated channel |
| Kcnd3os | potassium voltage-gated channel |
| Kcnf1 | potassium voltage-gated channel |
| Kcng2 | potassium voltage-gated channel |
| Kcnh1 | potassium voltage-gated channel |
| Kcnh5 | potassium voltage-gated channel |
| Kcnh8 | potassium voltage-gated channel |
| Kcnj15 | potassium inwardly-rectifying channel |
| Kcnj2 | potassium inwardly-rectifying channel |
| Kcnj3 | potassium inwardly-rectifying channel |
| Kcnj6 | potassium inwardly-rectifying channel |
| Kcnk10 | potassium channel |
| Kcnk12 | potassium channel |
| Kcnk15 | potassium channel |
| Kcnn4 | potassium intermediate/small conductance calcium-activated channel |
| Kcnq1 | potassium voltage-gated channel |
| Kcnq5 | potassium voltage-gated channel |
| Kcns2 | K+ voltage-gated channel |
| Kctd1 | potassium channel tetramerisation domain containing 1 |
| Kctd12 | potassium channel tetramerisation domain containing 12 |
| Kctd15 | potassium channel tetramerisation domain containing 15 |
| Kctd19 | potassium channel tetramerisation domain containing 19 |
| Kctd7 | potassium channel tetramerisation domain containing 7 |
| Kdm3b | KDM3B lysine (K)-specific demethylase 3B |
| Kdr | kinase insert domain protein receptor |
| Khdrbs2 | KH domain containing |
| Khdrbs3 | KH domain containing |
| Kidins220 | kinase D-interacting substrate 220 |
| Kif13a | kinesin family member 13A |
| Kif13b | kinesin family member 13B |
| Kif17 | kinesin family member 17 |
| Kif1b | kinesin family member 1B |
| Kif21a | kinesin family member 21A |
| Kif26b | kinesin family member 26B |
| Kif5b | kinesin family member 5B |
| Kif5c | kinesin family member 5C |
| Kifc3 | kinesin family member C3 |
| Kirrel3 | kin of IRRE like 3 (Drosophila) |
| Kit | KIT proto-oncogene receptor tyrosine kinase |
| Kiz | kizuna centrosomal protein |
| Klf12 | Kruppel-like factor 12 |
| Klf3 | Kruppel-like factor 3 (basic) |
| Klf4 | Kruppel-like factor 4 (gut) |
| Klf5 | Kruppel-like factor 5 |
| Klf6 | Kruppel-like factor 6 |
| Klf7 | Kruppel-like factor 7 (ubiquitous) |
| Klf8 | Kruppel-like factor 8 |
| Klhdc8a | kelch domain containing 8A |
| Klhl25 | kelch-like 25 |
| Klhl40 | kelch-like 40 |
| Klhl5 | kelch-like 5 |
| Klhl8 | kelch-like 8 |
| Klrk1 | killer cell lectin-like receptor subfamily K |
| Kntc1 | kinetochore associated 1 |
| Krr1 | KRR1 |
| Krt222 | keratin 222 |
| Ksr1 | kinase suppressor of ras 1 |
| Ksr2 | kinase suppressor of ras 2 |
| Ktn1 | kinectin 1 |
| Ky | kyphoscoliosis peptidase |
| L3mbtl4 | l(3)mbt-like 4 (Drosophila) |
| Lactbl1 | lactamase |
| Lama4 | laminin |
| Lamc3 | laminin gamma 3 |
| Lamp5 | lysosomal-associated membrane protein family |
| Lars2 | leucyl-tRNA synthetase |
| Lbp | lipopolysaccharide binding protein |
| Lbr | lamin B receptor |
| Lclat1 | lysocardiolipin acyltransferase 1 |
| Lcn9 | lipocalin 9 |
| Lcp2 | lymphocyte cytosolic protein 2 |
| Ldhd | lactate dehydrogenase D |
| Ldlrad3 | low density lipoprotein receptor class A domain containing 3 |
| Ldlrad4 | low density lipoprotein receptor class A domain containing 4 |
| Lef1 | lymphoid enhancer binding factor 1 |
| Lgalsl | lectin |
| Lgmn | legumain |
| Lgr5 | leucine rich repeat containing G protein coupled receptor 5 |
| Lgr6 | leucine-rich repeat-containing G protein-coupled receptor 6 |
| Lhcgr | luteinizing hormone/choriogonadotropin receptor |
| Lhfp | lipoma HMGIC fusion partner |
| Lhfpl2 | lipoma HMGIC fusion partner-like 2 |
| Lhpp | phospholysine phosphohistidine inorganic pyrophosphate phosphatase |
| Lhx2 | LIM homeobox protein 2 |
| Lif | leukemia inhibitory factor |
| Lifr | leukemia inhibitory factor receptor |
| Limch1 | LIM and calponin homology domains 1 |
| Lin52 | lin-52 homolog (C. elegans) |
| Lin54 | lin-54 homolog (C. elegans) |
| Lincred1 | long intergenic non-protein coding RNA of erythroid differentiation 1 |
| Lipk | lipase |
| Lix1 | limb and CNS expressed 1 |
| Lmbr1 | limb region 1 |
| Lmntd1 | lamin tail domain containing 1 |
| Lmo4 | LIM domain only 4 |
| Lmx1a | LIM homeobox transcription factor 1 alpha |
| Lncbate1 | brown adipose tissue enriched long non-coding RNA 1 |
| Lncenc1 | long non-coding RNA |
| Lnpep | leucyl/cystinyl aminopeptidase |
| LOC100861615 | alpha takusan-like |
| LOC105245869 | uncharacterized LOC105245869 |
| Lonp2 | lon peptidase 2 |
| Lonrf1 | LON peptidase N-terminal domain and ring finger 1 |
| Lonrf3 | LON peptidase N-terminal domain and ring finger 3 |
| Lpar1 | lysophosphatidic acid receptor 1 |
| Lpar3 | lysophosphatidic acid receptor 3 |
| Lpar4 | lysophosphatidic acid receptor 4 |
| Lpcat3 | lysophosphatidylcholine acyltransferase 3 |
| Lpgat1 | lysophosphatidylglycerol acyltransferase 1 |
| Lpin1 | lipin 1 |
| Lpp | LIM domain containing preferred translocation partner in lipoma |
| Lrat | lecithin-retinol acyltransferase (phosphatidylcholine-retinol-O-acyltransferase) |
| Lrba | LPS-responsive beige-like anchor |
| Lrch1 | leucine-rich repeats and calponin homology (CH) domain containing 1 |
| Lrch3 | leucine-rich repeats and calponin homology (CH) domain containing 3 |
| Lrfn2 | leucine rich repeat and fibronectin type III domain containing 2 |
| Lrfn5 | leucine rich repeat and fibronectin type III domain containing 5 |
| Lrig1 | leucine-rich repeats and immunoglobulin-like domains 1 |
| Lrp12 | low density lipoprotein-related protein 12 |
| Lrp1b | low density lipoprotein-related protein 1B (deleted in tumors) |
| Lrp2 | low density lipoprotein receptor-related protein 2 |
| Lrp5 | low density lipoprotein receptor-related protein 5 |
| Lrp6 | low density lipoprotein receptor-related protein 6 |
| Lrrc1 | leucine rich repeat containing 1 |
| Lrrc10b | leucine rich repeat containing 10B |
| Lrrc28 | leucine rich repeat containing 28 |
| Lrrc38 | leucine rich repeat containing 38 |
| Lrrc3b | leucine rich repeat containing 3B |
| Lrrc55 | leucine rich repeat containing 55 |
| Lrrc57 | leucine rich repeat containing 57 |
| Lrrc72 | leucine rich repeat containing 72 |
| Lrrc8c | leucine rich repeat containing 8 family |
| Lrrc8d | leucine rich repeat containing 8D |
| Lrrk2 | leucine-rich repeat kinase 2 |
| Lrrn3 | leucine rich repeat protein 3 |
| Lsr | lipolysis stimulated lipoprotein receptor |
| Ly6c1 | lymphocyte antigen 6 complex |
| Ly6e | lymphocyte antigen 6 complex |
| Ly75 | lymphocyte antigen 75 |
| Lyn | LYN proto-oncogene |
| Lypd6 | LY6/PLAUR domain containing 6 |
| Lypd6b | LY6/PLAUR domain containing 6B |
| Lyrm1 | LYR motif containing 1 |
| Lyrm7 | LYR motif containing 7 |
| Lyst | lysosomal trafficking regulator |
| Lyzl1 | lysozyme-like 1 |
| Lzts1 | leucine zipper |
| M1ap | meiosis 1 associated protein |
| Mab21l3 | mab-21-like 3 (C. elegans) |
| Macf1 | microtubule-actin crosslinking factor 1 |
| Macrod1 | MACRO domain containing 1 |
| Mad1l1 | MAD1 mitotic arrest deficient 1-like 1 |
| Mad2l1 | MAD2 mitotic arrest deficient-like 1 |
| Maf | avian musculoaponeurotic fibrosarcoma oncogene homolog |
| Magee2 | melanoma antigen |
| Magi2 | membrane associated guanylate kinase |
| Malt1 | MALT1 paracaspase |
| Maml2 | mastermind like 2 (Drosophila) |
| Maml3 | mastermind like 3 (Drosophila) |
| Man1a | mannosidase 1 |
| Man1a2 | mannosidase |
| Man2a1 | mannosidase 2 |
| Map1b | microtubule-associated protein 1B |
| Map2k6 | mitogen-activated protein kinase kinase 6 |
| Map3k13 | mitogen-activated protein kinase kinase kinase 13 |
| Map3k14 | mitogen-activated protein kinase kinase kinase 14 |
| Map3k20 | mitogen-activated protein kinase kinase kinase 20 |
| Map3k4 | mitogen-activated protein kinase kinase kinase 4 |
| Map3k5 | mitogen-activated protein kinase kinase kinase 5 |
| Map3k7 | mitogen-activated protein kinase kinase kinase 7 |
| Map4k3 | mitogen-activated protein kinase kinase kinase kinase 3 |
| Map4k4 | mitogen-activated protein kinase kinase kinase kinase 4 |
| Map4k5 | mitogen-activated protein kinase kinase kinase kinase 5 |
| Map7 | microtubule-associated protein 7 |
| Map7d2 | MAP7 domain containing 2 |
| Mapk10 | mitogen-activated protein kinase 10 |
| 4-Mar | membrane-associated ring finger (C3HC4) 4 |
| Marcks | myristoylated alanine rich protein kinase C substrate |
| Mark2 | MAP/microtubule affinity regulating kinase 2 |
| Mast4 | microtubule associated serine/threonine kinase family member 4 |
| Max | Max protein |
| Mbnl2 | muscleblind-like 2 |
| Mboat1 | membrane bound O-acyltransferase domain containing 1 |
| Mcoln3 | mucolipin 3 |
| Mctp1 | multiple C2 domains |
| Mdfic | MyoD family inhibitor domain containing |
| Mdga1 | MAM domain containing glycosylphosphatidylinositol anchor 1 |
| Mdm1 | transformed mouse 3T3 cell double minute 1 |
| Mdm2 | transformed mouse 3T3 cell double minute 2 |
| Me3 | malic enzyme 3 |
| Med12l | mediator complex subunit 12-like |
| Med13 | mediator complex subunit 13 |
| Med13l | mediator complex subunit 13-like |
| Med27 | mediator complex subunit 27 |
| Med30 | mediator complex subunit 30 |
| Mefv | Mediterranean fever |
| Melk | maternal embryonic leucine zipper kinase |
| Mesp1 | mesoderm posterior 1 |
| Metrnl | meteorin |
| Mex3b | mex3 RNA binding family member B |
| Mfge8 | milk fat globule-EGF factor 8 protein |
| Mfhas1 | malignant fibrous histiocytoma amplified sequence 1 |
| Mfsd4a | major facilitator superfamily domain containing 4A |
| Mfsd4b4 | major facilitator superfamily domain containing 4B4 |
| Mgat1 | mannoside acetylglucosaminyltransferase 1 |
| Mgat5 | mannoside acetylglucosaminyltransferase 5 |
| Mgme1 | mitochondrial genome maintenance exonuclease 1 |
| Mical3 | microtubule associated monooxygenase |
| Micu2 | mitochondrial calcium uptake 2 |
| Minpp1 | multiple inositol polyphosphate histidine phosphatase 1 |
| Mir128-2 | microRNA 128-2 |
| Mir129-1 | microRNA 129-1 |
| Mir145b | microRNA 145b |
| Mir148a | microRNA 148a |
| Mir17 | microRNA 17 |
| Mir181a-1 | microRNA 181a-1 |
| Mir1892 | microRNA 1892 |
| Mir1893 | microRNA 1893 |
| Mir1907 | microRNA 1907 |
| Mir21c | microRNA 21c |
| Mir28a | microRNA 28a |
| Mir3095 | microRNA 3095 |
| Mir30a | microRNA 30a |
| Mir3108 | microRNA 3108 |
| Mir3109 | microRNA 3109 |
| Mir32 | microRNA 32 |
| Mir34c | microRNA 34c |
| Mir363 | microRNA 363 |
| Mir365-2 | microRNA 365-2 |
| Mir378d | microRNA 378d |
| Mir3961 | microRNA 3961 |
| Mir467h | microRNA 467h |
| Mir5118 | microRNA 5118 |
| Mir5127 | microRNA 5127 |
| Mir5132 | microRNA 5132 |
| Mir6236 | microRNA 6236 |
| Mir6337 | microRNA 6337 |
| Mir6347 | microRNA 6347 |
| Mir6349 | microRNA 6349 |
| Mir6356 | microRNA 6356 |
| Mir6387 | microRNA 6387 |
| Mir6388 | microRNA 6388 |
| Mir6390 | microRNA 6390 |
| Mir6395 | microRNA 6395 |
| Mir6411 | microRNA 6411 |
| Mir6417 | microRNA 6417 |
| Mir670 | microRNA 670 |
| Mir6951 | microRNA 6951 |
| Mir700 | microRNA 700 |
| Mir7089 | microRNA 7089 |
| Mir7094-2 | microRNA 7094-2 |
| Mir7233 | microRNA 7233 |
| Mir7671 | microRNA 7671 |
| Mir7682 | microRNA 7682 |
| Mir8118 | microRNA 8118 |
| Mir8120 | microRNA 8120 |
| Mir9-3hg | Mir9-3 host gene |
| Mir92-2 | microRNA 92-2 |
| Mir98 | microRNA 98 |
| Mir99ahg | Mir99a and Mirlet7c-1 host gene (non-protein coding) |
| Mki67 | antigen identified by monoclonal antibody Ki 67 |
| Mkl1 | MKL (megakaryoblastic leukemia)/myocardin-like 1 |
| Mkln1 | muskelin 1 |
| Mks1 | Meckel syndrome |
| Mkx | mohawk homeobox |
| Mlec | malectin |
| Mlip | muscular LMNA-interacting protein |
| Mmaa | methylmalonic aciduria (cobalamin deficiency) type A |
| Mmd | monocyte to macrophage differentiation-associated |
| Mmp15 | matrix metallopeptidase 15 |
| Mmp27 | matrix metallopeptidase 27 |
| Mmrn1 | multimerin 1 |
| Mms19 | MMS19 (MET18 S. cerevisiae) |
| Mnat1 | menage a trois 1 |
| Mns1 | meiosis-specific nuclear structural protein 1 |
| Mob3b | MOB kinase activator 3B |
| Mob4 | MOB family member 4 |
| Mobp | myelin-associated oligodendrocytic basic protein |
| Mon2 | MON2 homolog |
| Moxd1 | monooxygenase |
| Mpdz | multiple PDZ domain protein |
| Mpl | myeloproliferative leukemia virus oncogene |
| Mpp5 | membrane protein |
| Mpp6 | membrane protein |
| Mrap2 | melanocortin 2 receptor accessory protein 2 |
| Mrgpra1 | MAS-related GPR |
| Mrpl1 | mitochondrial ribosomal protein L1 |
| Mrpl3 | mitochondrial ribosomal protein L3 |
| Mrpl44 | mitochondrial ribosomal protein L44 |
| Mrps22 | mitochondrial ribosomal protein S22 |
| Mrps28 | mitochondrial ribosomal protein S28 |
| Mrps33 | mitochondrial ribosomal protein S33 |
| Ms4a15 | membrane-spanning 4-domains |
| Ms4a4a | membrane-spanning 4-domains |
| Ms4a4d | membrane-spanning 4-domains |
| Msantd3 | Myb/SANT-like DNA-binding domain containing 3 |
| Msc | musculin |
| Msh3 | mutS homolog 3 |
| Msh6 | mutS homolog 6 |
| Msi2 | musashi RNA-binding protein 2 |
| Msn | moesin |
| Msra | methionine sulfoxide reductase A |
| Msx1os | msh homeobox 1 opposite strand |
| Mt3 | metallothionein 3 |
| Mtap | methylthioadenosine phosphorylase |
| Mtfr1 | mitochondrial fission regulator 1 |
| Mthfd1l | methylenetetrahydrofolate dehydrogenase (NADP+ dependent) 1-like |
| Mthfs | 5 |
| Mtif3 | mitochondrial translational initiation factor 3 |
| Mtss1 | metastasis suppressor 1 |
| Mtus2 | microtubule associated tumor suppressor candidate 2 |
| Mtx2 | metaxin 2 |
| Mup4 | major urinary protein 4 |
| Mvb12b | multivesicular body subunit 12B |
| Mxd1 | MAX dimerization protein 1 |
| Mxi1 | MAX interactor 1 |
| Myb | myeloblastosis oncogene |
| Mycn | v-myc avian myelocytomatosis viral related oncogene |
| Myh15 | myosin |
| Mylip | myosin regulatory light chain interacting protein |
| Mylk4 | myosin light chain kinase family |
| Myo10 | myosin X |
| Myo18a | myosin XVIIIA |
| Myo18b | myosin XVIIIb |
| Myo1b | myosin IB |
| Myo1e | myosin IE |
| Myo3a | myosin IIIA |
| Myo3b | myosin IIIB |
| Myo5a | myosin VA |
| Myo5b | myosin VB |
| Myoc | myocilin |
| Myom1 | myomesin 1 |
| Myom2 | myomesin 2 |
| Myt1l | myelin transcription factor 1-like |
| N4bp3 | NEDD4 binding protein 3 |
| NA | NA |
| Naa20 | N(alpha)-acetyltransferase 20 |
| Naa30 | N(alpha)-acetyltransferase 30 |
| Nab1 | Ngfi-A binding protein 1 |
| Nabp1 | nucleic acid binding protein 1 |
| Naip1 | NLR family |
| Nampt | nicotinamide phosphoribosyltransferase |
| Nans | N-acetylneuraminic acid synthase (sialic acid synthase) |
| Nars2 | asparaginyl-tRNA synthetase 2 (mitochondrial)(putative) |
| Nat8l | N-acetyltransferase 8-like |
| Nav1 | neuron navigator 1 |
| Nav2 | neuron navigator 2 |
| Nbea | neurobeachin |
| Nbl1 | neuroblastoma |
| Ncald | neurocalcin delta |
| Nck2 | non-catalytic region of tyrosine kinase adaptor protein 2 |
| Nckap5 | NCK-associated protein 5 |
| Ncmap | noncompact myelin associated protein |
| Ncoa2 | nuclear receptor coactivator 2 |
| Ncoa7 | nuclear receptor coactivator 7 |
| Ncs1 | neuronal calcium sensor 1 |
| Ndrg1 | N-myc downstream regulated gene 1 |
| Ndrg3 | N-myc downstream regulated gene 3 |
| Ndst3 | N-deacetylase/N-sulfotransferase (heparan glucosaminyl) 3 |
| Ndufa12 | NADH dehydrogenase (ubiquinone) 1 alpha subcomplex |
| Ndufaf2 | NADH dehydrogenase (ubiquinone) 1 alpha subcomplex |
| Neb | nebulin |
| Nedd4l | neural precursor cell expressed |
| Nefl | neurofilament |
| Nek1 | NIMA (never in mitosis gene a)-related expressed kinase 1 |
| Nek10 | NIMA (never in mitosis gene a)- related kinase 10 |
| Nek11 | NIMA (never in mitosis gene a)-related expressed kinase 11 |
| Nek6 | NIMA (never in mitosis gene a)-related expressed kinase 6 |
| Nek7 | NIMA (never in mitosis gene a)-related expressed kinase 7 |
| Nell2 | NEL-like 2 |
| Neo1 | neogenin |
| Nepro | nucleolus and neural progenitor protein |
| Net1 | neuroepithelial cell transforming gene 1 |
| Neto1 | neuropilin (NRP) and tolloid (TLL)-like 1 |
| Neu3 | neuraminidase 3 |
| Neu4 | sialidase 4 |
| Neurod1 | neurogenic differentiation 1 |
| Nexn | nexilin |
| Nfam1 | Nfat activating molecule with ITAM motif 1 |
| Nfe2l3 | nuclear factor |
| Nfia | nuclear factor I/A |
| Nfix | nuclear factor I/X |
| Ngef | neuronal guanine nucleotide exchange factor |
| Ngfr | nerve growth factor receptor (TNFR superfamily |
| Ngly1 | N-glycanase 1 |
| Nhlh2 | nescient helix loop helix 2 |
| Nhsl1 | NHS-like 1 |
| Nhsl2 | NHS-like 2 |
| Nid1 | nidogen 1 |
| Nid2 | nidogen 2 |
| Ninj2 | ninjurin 2 |
| Nipal1 | NIPA-like domain containing 1 |
| Nkain1 | Na+/K+ transporting ATPase interacting 1 |
| Nkx1-2 | NK1 transcription factor related |
| Nkx2-1 | NK2 homeobox 1 |
| Nkx2-2os | NK2 homeobox 2 |
| Nkx2-9 | NK2 homeobox 9 |
| Nkx3-1 | NK-3 transcription factor |
| Nkx6-1 | NK6 homeobox 1 |
| Nkx6-2 | NK6 homeobox 2 |
| Nlgn1 | neuroligin 1 |
| Nlk | nemo like kinase |
| Nln | neurolysin (metallopeptidase M3 family) |
| Nlrc4 | NLR family |
| Nlrp1b | NLR family |
| Nme4 | NME/NM23 nucleoside diphosphate kinase 4 |
| Nme7 | NME/NM23 family member 7 |
| Nme9 | NME/NM23 family member 9 |
| Nmnat3 | nicotinamide nucleotide adenylyltransferase 3 |
| Nnmt | nicotinamide N-methyltransferase |
| Noct | nocturnin |
| Nol4l | nucleolar protein 4-like |
| Nol9 | nucleolar protein 9 |
| Nos2 | nitric oxide synthase 2 |
| Notch2 | notch 2 |
| Notch4 | notch 4 |
| Npas2 | neuronal PAS domain protein 2 |
| Npas3 | neuronal PAS domain protein 3 |
| Npbwr1 | neuropeptides B/W receptor 1 |
| Npepps | aminopeptidase puromycin sensitive |
| Npff | neuropeptide FF-amide peptide precursor |
| Npffr1 | neuropeptide FF receptor 1 |
| Npm3-ps1 | nucleoplasmin 3 |
| Npnt | nephronectin |
| Nppc | natriuretic peptide type C |
| Npr1 | natriuretic peptide receptor 1 |
| Nptn | neuroplastin |
| Npvf | neuropeptide VF precursor |
| Npy | neuropeptide Y |
| Nr1h4 | nuclear receptor subfamily 1 |
| Nr1i2 | nuclear receptor subfamily 1 |
| Nr3c1 | nuclear receptor subfamily 3 |
| Nr4a3 | nuclear receptor subfamily 4 |
| Nr5a2 | nuclear receptor subfamily 5 |
| Nrbf2 | nuclear receptor binding factor 2 |
| Nrcam | neuronal cell adhesion molecule |
| Nrg2 | neuregulin 2 |
| Nrg4 | neuregulin 4 |
| Nrip1 | nuclear receptor interacting protein 1 |
| Nrip3 | nuclear receptor interacting protein 3 |
| Nrn1 | neuritin 1 |
| Nrp1 | neuropilin 1 |
| Nrxn1 | neurexin I |
| Nrxn3 | neurexin III |
| Nsg1 | neuron specific gene family member 1 |
| Nsg2 | neuron specific gene family member 2 |
| Nsmaf | neutral sphingomyelinase (N-SMase) activation associated factor |
| Nsmce1 | NSE1 homolog |
| Nsmce2 | NSE2/MMS21 homolog |
| Nsun6 | NOL1/NOP2/Sun domain family member 6 |
| Nt5c2 | 5'-nucleotidase |
| Nt5dc3 | 5'-nucleotidase domain containing 3 |
| Nt5e | 5' nucleotidase |
| Ntm | neurotrimin |
| Ntn1 | netrin 1 |
| Ntn4 | netrin 4 |
| Ntng1 | netrin G1 |
| Ntrk2 | neurotrophic tyrosine kinase |
| Nuak1 | NUAK family |
| Nucb2 | nucleobindin 2 |
| Nudcd2 | NudC domain containing 2 |
| Nudt4 | nudix (nucleoside diphosphate linked moiety X)-type motif 4 |
| Nufip1 | nuclear fragile X mental retardation protein interacting protein 1 |
| Nup210 | nucleoporin 210 |
| Nup210l | nucleoporin 210-like |
| Nupl1 | nucleoporin like 1 |
| Nwd1 | NACHT and WD repeat domain containing 1 |
| Nwd2 | NACHT and WD repeat domain containing 2 |
| Oaf | out at first homolog |
| Oas2 | 2'-5' oligoadenylate synthetase 2 |
| Oasl1 | 2'-5' oligoadenylate synthetase-like 1 |
| Odc1 | ornithine decarboxylase |
| Olfm1 | olfactomedin 1 |
| Olfm5 | olfactomedin 5 |
| Olfr1110 | olfactory receptor 1110 |
| Olfr1385 | olfactory receptor 1385 |
| Olfr1507 | olfactory receptor 1507 |
| Olfr1509 | olfactory receptor 1509 |
| Olfr1510 | olfactory receptor 1510 |
| Olfr231 | olfactory receptor 231 |
| Olfr279 | olfactory receptor 279 |
| Olfr427 | olfactory receptor 427 |
| Olfr536 | olfactory receptor 536 |
| Olfr94 | olfactory receptor 94 |
| Olig1 | oligodendrocyte transcription factor 1 |
| Olig2 | oligodendrocyte transcription factor 2 |
| Olig3 | oligodendrocyte transcription factor 3 |
| Opa1 | OPA1 |
| Optc | opticin |
| Optn | optineurin |
| Orc5 | origin recognition complex |
| Osbpl10 | oxysterol binding protein-like 10 |
| Osbpl11 | oxysterol binding protein-like 11 |
| Osbpl1a | oxysterol binding protein-like 1A |
| Osbpl3 | oxysterol binding protein-like 3 |
| Osbpl6 | oxysterol binding protein-like 6 |
| Osbpl9 | oxysterol binding protein-like 9 |
| Otud1 | OTU domain containing 1 |
| Otud4 | OTU domain containing 4 |
| Otx1 | orthodenticle homeobox 1 |
| Oxtr | oxytocin receptor |
| P2rx5 | purinergic receptor P2X |
| P4ha2 | procollagen-proline |
| Pabpc2 | poly(A) binding protein |
| Pacsin1 | protein kinase C and casein kinase substrate in neurons 1 |
| Pacsin2 | protein kinase C and casein kinase substrate in neurons 2 |
| Padi1 | peptidyl arginine deiminase |
| Padi4 | peptidyl arginine deiminase |
| Pag1 | phosphoprotein associated with glycosphingolipid microdomains 1 |
| Pak1ip1 | PAK1 interacting protein 1 |
| Pakap | paralemmin A kinase anchor protein |
| Palld | palladin |
| Pam | peptidylglycine alpha-amidating monooxygenase |
| Panx1 | pannexin 1 |
| Papd4 | PAP associated domain containing 4 |
| Papss2 | 3'-phosphoadenosine 5'-phosphosulfate synthase 2 |
| Paqr3 | progestin and adipoQ receptor family member III |
| Paqr5 | progestin and adipoQ receptor family member V |
| Pard3 | par-3 family cell polarity regulator |
| Pard3b | par-3 family cell polarity regulator beta |
| Parg | poly (ADP-ribose) glycohydrolase |
| Park7 | Parkinson disease (autosomal recessive |
| Parp8 | poly (ADP-ribose) polymerase family |
| Parvg | parvin |
| Patj | PATJ |
| Patz1 | POZ (BTB) and AT hook containing zinc finger 1 |
| Pax1 | paired box 1 |
| Pax5 | paired box 5 |
| Pcca | propionyl-Coenzyme A carboxylase |
| Pcdhac2 | protocadherin alpha subfamily C |
| Pcm1 | pericentriolar material 1 |
| Pcnx2 | pecanex homolog 2 |
| Pcsk1 | proprotein convertase subtilisin/kexin type 1 |
| Pcsk5 | proprotein convertase subtilisin/kexin type 5 |
| Pcsk6 | proprotein convertase subtilisin/kexin type 6 |
| Pde10a | phosphodiesterase 10A |
| Pde11a | phosphodiesterase 11A |
| Pde1a | phosphodiesterase 1A |
| Pde4b | phosphodiesterase 4B |
| Pde4d | phosphodiesterase 4D |
| Pde5a | phosphodiesterase 5A |
| Pde7a | phosphodiesterase 7A |
| Pde8b | phosphodiesterase 8B |
| Pde9a | phosphodiesterase 9A |
| Pdgfb | platelet derived growth factor |
| Pdia3 | protein disulfide isomerase associated 3 |
| Pdia5 | protein disulfide isomerase associated 5 |
| Pdlim5 | PDZ and LIM domain 5 |
| Pdpn | podoplanin |
| Pds5b | PDS5 cohesin associated factor B |
| Pdss1 | prenyl (solanesyl) diphosphate synthase |
| Pdzd2 | PDZ domain containing 2 |
| Pdzrn3 | PDZ domain containing RING finger 3 |
| Peli1 | pellino 1 |
| Peli2 | pellino 2 |
| Pelo | pelota homolog (Drosophila) |
| Pemt | phosphatidylethanolamine N-methyltransferase |
| Pex14 | peroxisomal biogenesis factor 14 |
| Pex26 | peroxisomal biogenesis factor 26 |
| Pex5l | peroxisomal biogenesis factor 5-like |
| Pex7 | peroxisomal biogenesis factor 7 |
| Pfkp | phosphofructokinase |
| Pgm3 | phosphoglucomutase 3 |
| Pgr15l | G protein-coupled receptor 15-like |
| Pgrmc2 | progesterone receptor membrane component 2 |
| Phactr2 | phosphatase and actin regulator 2 |
| Phactr3 | phosphatase and actin regulator 3 |
| Phb | prohibitin |
| Phc2 | polyhomeotic-like 2 (Drosophila) |
| Phc3 | polyhomeotic-like 3 (Drosophila) |
| Phf14 | PHD finger protein 14 |
| Phf2 | PHD finger protein 2 |
| Phf20 | PHD finger protein 20 |
| Phf24 | PHD finger protein 24 |
| Phf3 | PHD finger protein 3 |
| Phka2 | phosphorylase kinase alpha 2 |
| Phlda1 | pleckstrin homology like domain |
| Phtf2 | putative homeodomain transcription factor 2 |
| Phxr4 | per-hexamer repeat gene 4 |
| Pias1 | protein inhibitor of activated STAT 1 |
| Pias2 | protein inhibitor of activated STAT 2 |
| Pid1 | phosphotyrosine interaction domain containing 1 |
| Pigk | phosphatidylinositol glycan anchor biosynthesis |
| Pigz | phosphatidylinositol glycan anchor biosynthesis |
| Pik3c2a | phosphatidylinositol 3-kinase |
| Pik3cg | phosphoinositide-3-kinase |
| Pik3r1 | phosphatidylinositol 3-kinase |
| Pik3r4 | phosphatidylinositol 3 kinase |
| Pim1 | proviral integration site 1 |
| Pip4k2a | phosphatidylinositol-5-phosphate 4-kinase |
| Pip5k1b | phosphatidylinositol-4-phosphate 5-kinase |
| Pirt | phosphoinositide-interacting regulator of transient receptor potential channels |
| Pitpnb | phosphatidylinositol transfer protein |
| Pitpnc1 | phosphatidylinositol transfer protein |
| Pitpnm2 | phosphatidylinositol transfer protein |
| Piwil2 | piwi-like RNA-mediated gene silencing 2 |
| Pja2 | praja 2 |
| Pkdrej | polycystin (PKD) family receptor for egg jelly |
| Pkib | protein kinase inhibitor beta |
| Pkn2 | protein kinase N2 |
| Pkp4 | plakophilin 4 |
| Pla2g12a | phospholipase A2 |
| Pla2g4a | phospholipase A2 |
| Pla2r1 | phospholipase A2 receptor 1 |
| Plat | plasminogen activator |
| Platr22 | pluripotency associated transcript 22 |
| Platr4 | pluripotency associated transcript 4 |
| Plau | plasminogen activator |
| Plaur | plasminogen activator |
| Plcb4 | phospholipase C |
| Pld1 | phospholipase D1 |
| Plekha1 | pleckstrin homology domain containing |
| Plekha5 | pleckstrin homology domain containing |
| Plekha7 | pleckstrin homology domain containing |
| Plekha8 | pleckstrin homology domain containing |
| Plekhg6 | pleckstrin homology domain containing |
| Plekhh2 | pleckstrin homology domain containing |
| Plekhs1 | pleckstrin homology domain containing |
| Plet1 | placenta expressed transcript 1 |
| Plin2 | perilipin 2 |
| Plp1 | proteolipid protein (myelin) 1 |
| Plpp1 | phospholipid phosphatase 1 |
| Plpp3 | phospholipid phosphatase 3 |
| Plpp4 | phospholipid phosphatase 4 |
| Plscr5 | phospholipid scramblase family |
| Plxna4 | plexin A4 |
| Plxnc1 | plexin C1 |
| Pmp2 | peripheral myelin protein 2 |
| Pmp22 | peripheral myelin protein 22 |
| Pnkd | paroxysmal nonkinesiogenic dyskinesia |
| Pnma2 | paraneoplastic antigen MA2 |
| Pnmal2 | PNMA-like 2 |
| Pnoc | prepronociceptin |
| Poc1b | POC1 centriolar protein B |
| Pofut1 | protein O-fucosyltransferase 1 |
| Pola1 | polymerase (DNA directed) |
| Pole4 | polymerase (DNA-directed) |
| Polh | polymerase (DNA directed) |
| Poln | DNA polymerase N |
| Polr1a | polymerase (RNA) I polypeptide A |
| Pomt2 | protein-O-mannosyltransferase 2 |
| Por | P450 (cytochrome) oxidoreductase |
| Postn | periostin |
| Pou1f1 | POU domain |
| Pou2af1 | POU domain |
| Pou2f1 | POU domain |
| Pou3f2 | POU domain |
| Pou4f2 | POU domain |
| Ppa2 | pyrophosphatase (inorganic) 2 |
| Pparg | peroxisome proliferator activated receptor gamma |
| Ppargc1b | peroxisome proliferative activated receptor |
| Ppcdc | phosphopantothenoylcysteine decarboxylase |
| Ppef1 | protein phosphatase with EF hand calcium-binding domain 1 |
| Ppfia4 | protein tyrosine phosphatase |
| Ppfibp1 | PTPRF interacting protein |
| Ppfibp2 | PTPRF interacting protein |
| Ppic | peptidylprolyl isomerase C |
| Ppip5k2 | diphosphoinositol pentakisphosphate kinase 2 |
| Ppm1b | protein phosphatase 1B |
| Ppm1h | protein phosphatase 1H (PP2C domain containing) |
| Ppm1l | protein phosphatase 1 (formerly 2C)-like |
| Ppm1m | protein phosphatase 1M |
| Ppp1cb | protein phosphatase 1 |
| Ppp1r1c | protein phosphatase 1 |
| Ppp1r21 | protein phosphatase 1 |
| Ppp1r3b | protein phosphatase 1 |
| Ppp1r3g | protein phosphatase 1 |
| Ppp2r2a | protein phosphatase 2 |
| Ppp2r2b | protein phosphatase 2 |
| Ppp2r5a | protein phosphatase 2 |
| Ppp2r5c | protein phosphatase 2 |
| Ppp2r5e | protein phosphatase 2 |
| Ppp3r1 | protein phosphatase 3 |
| Ppp4r1l-ps | protein phosphatase 4 |
| Ppp4r2 | protein phosphatase 4 |
| Ppp6r3 | protein phosphatase 6 |
| Ppt1 | palmitoyl-protein thioesterase 1 |
| Pptc7 | PTC7 protein phosphatase homolog |
| Prag1 | PEAK1 related kinase activating pseudokinase 1 |
| Pramel1 | preferentially expressed antigen in melanoma-like 1 |
| Prdm11 | PR domain containing 11 |
| Prdm5 | PR domain containing 5 |
| Prdm6 | PR domain containing 6 |
| Prdx4 | peroxiredoxin 4 |
| Prdx6 | peroxiredoxin 6 |
| Prep | prolyl endopeptidase |
| Prg4 | proteoglycan 4 (megakaryocyte stimulating factor |
| Prickle1 | prickle planar cell polarity protein 1 |
| Prkag2 | protein kinase |
| Prkch | protein kinase C |
| Prkg2 | protein kinase |
| Prl2c3 | prolactin family 2 |
| Prlr | prolactin receptor |
| Prmt8 | protein arginine N-methyltransferase 8 |
| Proc | protein C |
| Prodh | proline dehydrogenase |
| Prok2 | prokineticin 2 |
| Proser2 | proline and serine rich 2 |
| Prpf18 | pre-mRNA processing factor 18 |
| Prph2 | peripherin 2 |
| Prps1l1 | phosphoribosyl pyrophosphate synthetase 1-like 1 |
| Prps2 | phosphoribosyl pyrophosphate synthetase 2 |
| Prr29 | proline rich 29 |
| Prr5l | proline rich 5 like |
| Prrc1 | proline-rich coiled-coil 1 |
| Prrt4 | proline-rich transmembrane protein 4 |
| Prss23 | protease |
| Prss46 | protease |
| Prune2 | prune homolog 2 |
| Psd3 | pleckstrin and Sec7 domain containing 3 |
| Psen2 | presenilin 2 |
| Psmb7 | proteasome (prosome |
| Psmd11 | proteasome (prosome |
| Psmd7 | proteasome (prosome |
| Psme4 | proteasome (prosome |
| Pstpip2 | proline-serine-threonine phosphatase-interacting protein 2 |
| Ptch1 | patched 1 |
| Ptchd1 | patched domain containing 1 |
| Ptchd3 | patched domain containing 3 |
| Ptger3 | prostaglandin E receptor 3 (subtype EP3) |
| Ptgfrn | prostaglandin F2 receptor negative regulator |
| Ptk2 | PTK2 protein tyrosine kinase 2 |
| Ptk2b | PTK2 protein tyrosine kinase 2 beta |
| Ptma | prothymosin alpha |
| Ptpn1 | protein tyrosine phosphatase |
| Ptpn13 | protein tyrosine phosphatase |
| Ptpn14 | protein tyrosine phosphatase |
| Ptpn4 | protein tyrosine phosphatase |
| Ptprf | protein tyrosine phosphatase |
| Ptprj | protein tyrosine phosphatase |
| Ptprk | protein tyrosine phosphatase |
| Ptpro | protein tyrosine phosphatase |
| Ptprq | protein tyrosine phosphatase |
| Ptpru | protein tyrosine phosphatase |
| Pum1 | pumilio RNA-binding family member 1 |
| Pum2 | pumilio RNA-binding family member 2 |
| Pvt1 | plasmacytoma variant translocation 1 |
| Pxk | PX domain containing serine/threonine kinase |
| Pxmp2 | peroxisomal membrane protein 2 |
| Pxylp1 | 2-phosphoxylose phosphatase 1 |
| Pygo1 | pygopus 1 |
| Qpct | glutaminyl-peptide cyclotransferase (glutaminyl cyclase) |
| Qrich1 | glutamine-rich 1 |
| Rab11b | RAB11B |
| Rab11fip2 | RAB11 family interacting protein 2 (class I) |
| Rab1a | RAB1A |
| Rab27a | RAB27A |
| Rab2b | RAB2B |
| Rab33b | RAB33B |
| Rab3gap2 | RAB3 GTPase activating protein subunit 2 |
| Rab3ip | RAB3A interacting protein |
| Rab7b | RAB7B |
| Rabgap1l | RAB GTPase activating protein 1-like |
| Rad23b | RAD23 homolog B |
| Rad51b | RAD51 paralog B |
| Rad54b | RAD54 homolog B (S. cerevisiae) |
| Rag2 | recombination activating gene 2 |
| Rai1 | retinoic acid induced 1 |
| Ralb | v-ral simian leukemia viral oncogene B |
| Ralgapa2 | Ral GTPase activating protein |
| Ralgps2 | Ral GEF with PH domain and SH3 binding motif 2 |
| Ralyl | RALY RNA binding protein-like |
| Ramp1 | receptor (calcitonin) activity modifying protein 1 |
| Ranbp2 | RAN binding protein 2 |
| Rap1a | RAS-related protein-1a |
| Rap1b | RAS related protein 1b |
| Rap2b | RAP2B |
| Rapgef1 | Rap guanine nucleotide exchange factor (GEF) 1 |
| Rapgef2 | Rap guanine nucleotide exchange factor (GEF) 2 |
| Rapgef4 | Rap guanine nucleotide exchange factor (GEF) 4 |
| Rapgef5 | Rap guanine nucleotide exchange factor (GEF) 5 |
| Rapgef6 | Rap guanine nucleotide exchange factor (GEF) 6 |
| Rasa1 | RAS p21 protein activator 1 |
| Rasa2 | RAS p21 protein activator 2 |
| Rasa3 | RAS p21 protein activator 3 |
| Rasal2 | RAS protein activator like 2 |
| Rasal3 | RAS protein activator like 3 |
| Rasgrf2 | RAS protein-specific guanine nucleotide-releasing factor 2 |
| Rasgrp1 | RAS guanyl releasing protein 1 |
| Rasgrp3 | RAS |
| Rassf2 | Ras association (RalGDS/AF-6) domain family member 2 |
| Rassf4 | Ras association (RalGDS/AF-6) domain family member 4 |
| Rb1 | RB transcriptional corepressor 1 |
| Rbbp8 | retinoblastoma binding protein 8 |
| Rbbp8nl | RBBP8 N-terminal like |
| Rbfox1 | RNA binding protein |
| Rbl2 | RB transcriptional corepressor like 2 |
| Rbm11 | RNA binding motif protein 11 |
| Rbm20 | RNA binding motif protein 20 |
| Rbm24 | RNA binding motif protein 24 |
| Rbm27 | RNA binding motif protein 27 |
| Rbm46 | RNA binding motif protein 46 |
| Rbms1 | RNA binding motif |
| Rbms2 | RNA binding motif |
| Rbms3 | RNA binding motif |
| Rbmxl2 | RNA binding motif protein |
| Rbp4 | retinol binding protein 4 |
| Rbpj | recombination signal binding protein for immunoglobulin kappa J region |
| Rbpms | RNA binding protein gene with multiple splicing |
| Rcan1 | regulator of calcineurin 1 |
| Rcan2 | regulator of calcineurin 2 |
| Rcan3 | regulator of calcineurin 3 |
| Rcbtb2 | regulator of chromosome condensation (RCC1) and BTB (POZ) domain containing protein 2 |
| Rcor1 | REST corepressor 1 |
| Rcsd1 | RCSD domain containing 1 |
| Rd3 | retinal degeneration 3 |
| Rdh8 | retinol dehydrogenase 8 |
| Redrum | Redrum |
| Reep1 | receptor accessory protein 1 |
| Reln | reelin |
| Ren2 | renin 2 tandem duplication of Ren1 |
| Reps1 | RalBP1 associated Eps domain containing protein |
| Rere | arginine glutamic acid dipeptide (RE) repeats |
| Rerg | RAS-like |
| Retnlb | resistin like beta |
| Rev3l | REV3 like |
| Rfc1 | replication factor C (activator 1) 1 |
| Rffl | ring finger and FYVE like domain containing protein |
| Rflna | refilin A |
| Rflnb | refilin B |
| Rftn2 | raftlin family member 2 |
| Rfx4 | regulatory factor X |
| Rfx8 | regulatory factor X 8 |
| Rgs1 | regulator of G-protein signaling 1 |
| Rgs2 | regulator of G-protein signaling 2 |
| Rgs20 | regulator of G-protein signaling 20 |
| Rgs21 | regulator of G-protein signalling 21 |
| Rgs3 | regulator of G-protein signaling 3 |
| Rgs6 | regulator of G-protein signaling 6 |
| Rgs9 | regulator of G-protein signaling 9 |
| Rgsl1 | regulator of G-protein signaling like 1 |
| Rhbdd1 | rhomboid domain containing 1 |
| Rho | rhodopsin |
| Rhobtb1 | Rho-related BTB domain containing 1 |
| Rhobtb2 | Rho-related BTB domain containing 2 |
| Rhoh | ras homolog family member H |
| Rhoj | ras homolog family member J |
| Rhoq | ras homolog family member Q |
| Ric3 | RIC3 acetylcholine receptor chaperone |
| Rictor | RPTOR independent companion of MTOR |
| Rimklb | ribosomal modification protein rimK-like family member B |
| Rims1 | regulating synaptic membrane exocytosis 1 |
| Rims3 | regulating synaptic membrane exocytosis 3 |
| Rin2 | Ras and Rab interactor 2 |
| Ripply2 | ripply transcriptional repressor 2 |
| Rmi2 | RecQ mediated genome instability 2 |
| Rmnd1 | required for meiotic nuclear division 1 homolog |
| Rnaseh2b | ribonuclease H2 |
| Rnf125 | ring finger protein 125 |
| Rnf130 | ring finger protein 130 |
| Rnf145 | ring finger protein 145 |
| Rnf149 | ring finger protein 149 |
| Rnf150 | ring finger protein 150 |
| Rnf152 | ring finger protein 152 |
| Rnf157 | ring finger protein 157 |
| Rnf165 | ring finger protein 165 |
| Rnf169 | ring finger protein 169 |
| Rnf185 | ring finger protein 185 |
| Rnf19b | ring finger protein 19B |
| Rnf216 | ring finger protein 216 |
| Rnf217 | ring finger protein 217 |
| Rnf220 | ring finger protein 220 |
| Rnf224 | ring finger protein 224 |
| Rnf225 | ring finger protein 225 |
| Rnf32 | ring finger protein 32 |
| Rnf38 | ring finger protein 38 |
| Rnls | renalase |
| Rnu7 | U7 small nuclear RNA |
| Robo1 | roundabout guidance receptor 1 |
| Robo3 | roundabout guidance receptor 3 |
| Rock1 | Rho-associated coiled-coil containing protein kinase 1 |
| Rock2 | Rho-associated coiled-coil containing protein kinase 2 |
| Ror1 | receptor tyrosine kinase-like orphan receptor 1 |
| Rora | RAR-related orphan receptor alpha |
| Rpap3 | RNA polymerase II associated protein 3 |
| Rph3a | rabphilin 3A |
| Rpia | ribose 5-phosphate isomerase A |
| Rpl21 | ribosomal protein L21 |
| Rpl34 | ribosomal protein L34 |
| Rplp1 | ribosomal protein |
| Rprd2 | regulation of nuclear pre-mRNA domain containing 2 |
| Rprm | reprimo |
| Rps15a | ribosomal protein S15A |
| Rps21 | ribosomal protein S21 |
| Rps24 | ribosomal protein S24 |
| Rps6ka2 | ribosomal protein S6 kinase |
| Rps6ka3 | ribosomal protein S6 kinase polypeptide 3 |
| Rps6ka5 | ribosomal protein S6 kinase |
| Rps6kc1 | ribosomal protein S6 kinase polypeptide 1 |
| Rptor | regulatory associated protein of MTOR |
| Rras2 | related RAS viral (r-ras) oncogene 2 |
| Rreb1 | ras responsive element binding protein 1 |
| Rrm2b | ribonucleotide reductase M2 B (TP53 inducible) |
| Rrp1 | ribosomal RNA processing 1 homolog (S. cerevisiae) |
| Rsl24d1 | ribosomal L24 domain containing 1 |
| Rsph1 | radial spoke head 1 homolog (Chlamydomonas) |
| Rsph14 | radial spoke head homolog 14 (Chlamydomonas) |
| Rspo3 | R-spondin 3 |
| Rspo4 | R-spondin 4 |
| Rsrc1 | arginine/serine-rich coiled-coil 1 |
| Rsu1 | Ras suppressor protein 1 |
| Rtn1 | reticulon 1 |
| Rtn4 | reticulon 4 |
| Rufy1 | RUN and FYVE domain containing 1 |
| Rundc1 | RUN domain containing 1 |
| Rundc3b | RUN domain containing 3B |
| Runx1 | runt related transcription factor 1 |
| Runx2 | runt related transcription factor 2 |
| Rwdd3 | RWD domain containing 3 |
| Rxfp1 | relaxin/insulin-like family peptide receptor 1 |
| Rxfp2 | relaxin/insulin-like family peptide receptor 2 |
| Rybp | RING1 and YY1 binding protein |
| Ryk | receptor-like tyrosine kinase |
| Ryr2 | ryanodine receptor 2 |
| Ryr3 | ryanodine receptor 3 |
| S100a6 | S100 calcium binding protein A6 (calcyclin) |
| S1pr1 | sphingosine-1-phosphate receptor 1 |
| S1pr2 | sphingosine-1-phosphate receptor 2 |
| Sall1 | sal-like 1 (Drosophila) |
| Sall3 | sal-like 3 (Drosophila) |
| Samd4 | sterile alpha motif domain containing 4 |
| Samd4b | sterile alpha motif domain containing 4B |
| Samhd1 | SAM domain and HD domain |
| Sap130 | Sin3A associated protein |
| Sardh | sarcosine dehydrogenase |
| Sash1 | SAM and SH3 domain containing 1 |
| Sash3 | SAM and SH3 domain containing 3 |
| Satb1 | special AT-rich sequence binding protein 1 |
| Sbf2 | SET binding factor 2 |
| Sbk2 | SH3-binding domain kinase family |
| Sc5d | sterol-C5-desaturase |
| Scaf8 | SR-related CTD-associated factor 8 |
| Scamp5 | secretory carrier membrane protein 5 |
| Sccpdh | saccharopine dehydrogenase (putative) |
| Scfd2 | Sec1 family domain containing 2 |
| Scg2 | secretogranin II |
| Schip1 | schwannomin interacting protein 1 |
| Scin | scinderin |
| Scml4 | sex comb on midleg-like 4 (Drosophila) |
| Scn10a | sodium channel |
| Scn11a | sodium channel |
| Scn2b | sodium channel |
| Scn3b | sodium channel |
| Scp2 | sterol carrier protein 2 |
| Scrt2 | scratch family zinc finger 2 |
| Scube1 | signal peptide |
| Sdccag8 | serologically defined colon cancer antigen 8 |
| Sdhaf1 | succinate dehydrogenase complex assembly factor 1 |
| Sdk1 | sidekick cell adhesion molecule 1 |
| Sdk2 | sidekick cell adhesion molecule 2 |
| Sec1 | secretory blood group 1 |
| Sec11a | SEC11 homolog A |
| Sec24b | Sec24 related gene family |
| Sec61g | SEC61 |
| Secisbp2l | SECIS binding protein 2-like |
| Sel1l | sel-1 suppressor of lin-12-like (C. elegans) |
| Sel1l3 | sel-1 suppressor of lin-12-like 3 (C. elegans) |
| Sele | selectin |
| Selenof | selenoprotein F |
| Selenot | selenoprotein T |
| Sell | selectin |
| Selp | selectin |
| Sema3c | sema domain |
| Sema4d | sema domain |
| Sema6a | sema domain |
| Senp7 | SUMO1/sentrin specific peptidase 7 |
| 3-Sep | septin 3 |
| 9-Sep | septin 9 |
| Serhl | serine hydrolase-like |
| Serinc5 | serine incorporator 5 |
| Serp2 | stress-associated endoplasmic reticulum protein family member 2 |
| Serpinb5 | serine (or cysteine) peptidase inhibitor |
| Serpine2 | serine (or cysteine) peptidase inhibitor |
| Sertad2 | SERTA domain containing 2 |
| Sesn1 | sestrin 1 |
| Sesn3 | sestrin 3 |
| Sestd1 | SEC14 and spectrin domains 1 |
| Setbp1 | SET binding protein 1 |
| Setd5 | SET domain containing 5 |
| Setdb1 | SET domain |
| Setx | senataxin |
| Sfmbt2 | Scm-like with four mbt domains 2 |
| Sfrp2 | secreted frizzled-related protein 2 |
| Sfswap | splicing factor |
| Sfxn1 | sideroflexin 1 |
| Sgip1 | SH3-domain GRB2-like (endophilin) interacting protein 1 |
| Sgk1 | serum/glucocorticoid regulated kinase 1 |
| Sgk3 | serum/glucocorticoid regulated kinase 3 |
| Sgms1 | sphingomyelin synthase 1 |
| Sgpp1 | sphingosine-1-phosphate phosphatase 1 |
| Sgsm1 | small G protein signaling modulator 1 |
| Sgsm2 | small G protein signaling modulator 2 |
| Sh2d1a | SH2 domain containing 1A |
| Sh2d2a | SH2 domain containing 2A |
| Sh2d4a | SH2 domain containing 4A |
| Sh3bgrl2 | SH3 domain binding glutamic acid-rich protein like 2 |
| Sh3gl3 | SH3-domain GRB2-like 3 |
| Sh3kbp1 | SH3-domain kinase binding protein 1 |
| Sh3pxd2a | SH3 and PX domains 2A |
| Shank1 | SH3/ankyrin domain gene 1 |
| Shank3 | SH3/ankyrin domain gene 3 |
| Shb | src homology 2 domain-containing transforming protein B |
| She | src homology 2 domain-containing transforming protein E |
| Shisa2 | shisa family member 2 |
| Shoc2 | soc-2 (suppressor of clear) homolog (C. elegans) |
| Shq1 | SHQ1 homolog (S. cerevisiae) |
| Shroom3 | shroom family member 3 |
| Shtn1 | shootin 1 |
| Siah3 | seven in absentia homolog 3 (Drosophila) |
| Siglec15 | sialic acid binding Ig-like lectin 15 |
| Sik1 | salt inducible kinase 1 |
| Sik3 | SIK family kinase 3 |
| Sim2 | single-minded homolog 2 (Drosophila) |
| Sipa1l1 | signal-induced proliferation-associated 1 like 1 |
| Sipa1l2 | signal-induced proliferation-associated 1 like 2 |
| Sirpa | signal-regulatory protein alpha |
| Six3 | sine oculis-related homeobox 3 |
| Six4 | sine oculis-related homeobox 4 |
| Skap2 | src family associated phosphoprotein 2 |
| Skil | SKI-like |
| Skor1 | SKI family transcriptional corepressor 1 |
| Skor2 | SKI family transcriptional corepressor 2 |
| Skp1a | S-phase kinase-associated protein 1A |
| Slain1 | SLAIN motif family |
| Slamf7 | SLAM family member 7 |
| Slc10a1 | solute carrier family 10 (sodium/bile acid cotransporter family) |
| Slc12a2 | solute carrier family 12 |
| Slc12a6 | solute carrier family 12 |
| Slc14a2 | solute carrier family 14 (urea transporter) |
| Slc16a1 | solute carrier family 16 (monocarboxylic acid transporters) |
| Slc16a10 | solute carrier family 16 (monocarboxylic acid transporters) |
| Slc16a14 | solute carrier family 16 (monocarboxylic acid transporters) |
| Slc16a2 | solute carrier family 16 (monocarboxylic acid transporters) |
| Slc16a7 | solute carrier family 16 (monocarboxylic acid transporters) |
| Slc16a9 | solute carrier family 16 (monocarboxylic acid transporters) |
| Slc17a8 | solute carrier family 17 (sodium-dependent inorganic phosphate cotransporter) |
| Slc19a3 | solute carrier family 19 |
| Slc1a1 | solute carrier family 1 (neuronal/epithelial high affinity glutamate transporter |
| Slc1a2 | solute carrier family 1 (glial high affinity glutamate transporter) |
| Slc22a1 | solute carrier family 22 (organic cation transporter) |
| Slc22a15 | solute carrier family 22 (organic anion/cation transporter) |
| Slc22a16 | solute carrier family 22 (organic cation transporter) |
| Slc23a2 | solute carrier family 23 (nucleobase transporters) |
| Slc23a4 | solute carrier family 23 member 4 |
| Slc24a4 | solute carrier family 24 (sodium/potassium/calcium exchanger) |
| Slc24a5 | solute carrier family 24 |
| Slc25a12 | solute carrier family 25 (mitochondrial carrier |
| Slc25a24 | solute carrier family 25 (mitochondrial carrier |
| Slc25a25 | solute carrier family 25 (mitochondrial carrier |
| Slc25a33 | solute carrier family 25 |
| Slc25a53 | solute carrier family 25 |
| Slc26a8 | solute carrier family 26 |
| Slc27a6 | solute carrier family 27 (fatty acid transporter) |
| Slc29a3 | solute carrier family 29 (nucleoside transporters) |
| Slc2a12 | solute carrier family 2 (facilitated glucose transporter) |
| Slc2a5 | solute carrier family 2 (facilitated glucose transporter) |
| Slc30a5 | solute carrier family 30 (zinc transporter) |
| Slc30a7 | solute carrier family 30 (zinc transporter) |
| Slc35b3 | solute carrier family 35 |
| Slc35d1 | solute carrier family 35 (UDP-glucuronic acid/UDP-N-acetylgalactosamine dual transporter) |
| Slc35f1 | solute carrier family 35 |
| Slc35f4 | solute carrier family 35 |
| Slc35f5 | solute carrier family 35 |
| Slc36a3 | solute carrier family 36 (proton/amino acid symporter) |
| Slc37a3 | solute carrier family 37 (glycerol-3-phosphate transporter) |
| Slc38a2 | solute carrier family 38 |
| Slc38a6 | solute carrier family 38 |
| Slc39a10 | solute carrier family 39 (zinc transporter) |
| Slc39a6 | solute carrier family 39 (metal ion transporter) |
| Slc40a1 | solute carrier family 40 (iron-regulated transporter) |
| Slc41a2 | solute carrier family 41 |
| Slc41a3 | solute carrier family 41 |
| Slc43a2 | solute carrier family 43 |
| Slc44a3 | solute carrier family 44 |
| Slc44a5 | solute carrier family 44 |
| Slc45a1 | solute carrier family 45 |
| Slc45a4 | solute carrier family 45 |
| Slc46a1 | solute carrier family 46 |
| Slc47a1 | solute carrier family 47 |
| Slc4a10 | solute carrier family 4 |
| Slc4a11 | solute carrier family 4 |
| Slc4a4 | solute carrier family 4 (anion exchanger) |
| Slc4a7 | solute carrier family 4 |
| Slc5a8 | solute carrier family 5 (iodide transporter) |
| Slc7a2 | solute carrier family 7 (cationic amino acid transporter |
| Slc7a8 | solute carrier family 7 (cationic amino acid transporter |
| Slc8a3 | solute carrier family 8 (sodium/calcium exchanger) |
| Slc9a1 | solute carrier family 9 (sodium/hydrogen exchanger) |
| Slc9b2 | solute carrier family 9 |
| Slco2a1 | solute carrier organic anion transporter family |
| Slco3a1 | solute carrier organic anion transporter family |
| Slco5a1 | solute carrier organic anion transporter family |
| Slco6d1 | solute carrier organic anion transporter family |
| Slfn5os | schlafen 5 |
| Slit3 | slit homolog 3 (Drosophila) |
| Slitrk1 | SLIT and NTRK-like family |
| Sln | sarcolipin |
| Sltm | SAFB-like |
| Slx4 | SLX4 structure-specific endonuclease subunit homolog (S. cerevisiae) |
| Slx4ip | SLX4 interacting protein |
| Smad4 | SMAD family member 4 |
| Smad9 | SMAD family member 9 |
| Smap1 | small ArfGAP 1 |
| Smarca4 | SWI/SNF related |
| Smarce1 | SWI/SNF related |
| Smco4 | single-pass membrane protein with coiled-coil domains 4 |
| Smg6 | Smg-6 homolog |
| Smim14 | small integral membrane protein 14 |
| Smim20 | small integral membrane protein 20 |
| Smok4a | sperm motility kinase 4A |
| Sms | spermine synthase |
| Smtnl1 | smoothelin-like 1 |
| Smtnl2 | smoothelin-like 2 |
| Smurf1 | SMAD specific E3 ubiquitin protein ligase 1 |
| Smyd3 | SET and MYND domain containing 3 |
| Snapc1 | small nuclear RNA activating complex |
| Sncaip | synuclein |
| Snd1 | staphylococcal nuclease and tudor domain containing 1 |
| Snn | stannin |
| Snora26 | small nucleolar RNA |
| Snrnp40 | small nuclear ribonucleoprotein 40 (U5) |
| Snrpe | small nuclear ribonucleoprotein E |
| Sntb1 | syntrophin |
| Snx27 | sorting nexin family member 27 |
| Snx3 | sorting nexin 3 |
| Snx30 | sorting nexin family member 30 |
| Snx8 | sorting nexin 8 |
| Socs1 | suppressor of cytokine signaling 1 |
| Soga3 | SOGA family member 3 |
| Sorcs2 | sortilin-related VPS10 domain containing receptor 2 |
| Sorl1 | sortilin-related receptor |
| Sox17 | SRY (sex determining region Y)-box 17 |
| Sox4 | SRY (sex determining region Y)-box 4 |
| Sox5 | SRY (sex determining region Y)-box 5 |
| Sox6 | SRY (sex determining region Y)-box 6 |
| Sp100 | nuclear antigen Sp100 |
| Sp3 | trans-acting transcription factor 3 |
| Sp5 | trans-acting transcription factor 5 |
| Spaca1 | sperm acrosome associated 1 |
| Spaca6 | sperm acrosome associated 6 |
| Spag16 | sperm associated antigen 16 |
| Spag9 | sperm associated antigen 9 |
| Spast | spastin |
| Spata13 | spermatogenesis associated 13 |
| Spata5 | spermatogenesis associated 5 |
| Spats2 | spermatogenesis associated |
| Spats2l | spermatogenesis associated |
| Specc1 | sperm antigen with calponin homology and coiled-coil domains 1 |
| Speer2 | spermatogenesis associated glutamate (E)-rich protein 2 |
| Speer4f1 | spermatogenesis associated glutamate (E)-rich protein 4F1 |
| Spef2 | sperm flagellar 2 |
| Speg | SPEG complex locus |
| Spidr | scaffolding protein involved in DNA repair |
| Spink2 | serine peptidase inhibitor |
| Spo11 | SPO11 meiotic protein covalently bound to DSB |
| Spock1 | sparc/osteonectin |
| Spon1 | spondin 1 |
| Spred1 | sprouty protein with EVH-1 domain 1 |
| Spred2 | sprouty-related |
| Spry1 | sprouty homolog 1 (Drosophila) |
| Spry4 | sprouty homolog 4 (Drosophila) |
| Sptbn1 | spectrin beta |
| Sptbn2 | spectrin beta |
| Sptlc1 | serine palmitoyltransferase |
| Spty2d1 | SPT2 |
| Srbd1 | S1 RNA binding domain 1 |
| Src | Rous sarcoma oncogene |
| Srgap2 | SLIT-ROBO Rho GTPase activating protein 2 |
| Srgap3 | SLIT-ROBO Rho GTPase activating protein 3 |
| Srpk1 | serine/arginine-rich protein specific kinase 1 |
| Srpk2 | serine/arginine-rich protein specific kinase 2 |
| Srr | serine racemase |
| Srsf4 | serine/arginine-rich splicing factor 4 |
| Ss18 | SS18 |
| Ssbp2 | single-stranded DNA binding protein 2 |
| Ssh2 | slingshot homolog 2 (Drosophila) |
| Sspn | sarcospan |
| Sstr3 | somatostatin receptor 3 |
| St18 | suppression of tumorigenicity 18 |
| St3gal1 | ST3 beta-galactoside alpha-2 |
| St5 | suppression of tumorigenicity 5 |
| St6gal1 | beta galactoside alpha 2 |
| St6galnac3 | ST6 (alpha-N-acetyl-neuraminyl-2 |
| St6galnac5 | ST6 (alpha-N-acetyl-neuraminyl-2 |
| St7 | suppression of tumorigenicity 7 |
| St8sia1 | ST8 alpha-N-acetyl-neuraminide alpha-2 |
| St8sia5 | ST8 alpha-N-acetyl-neuraminide alpha-2 |
| Stab2 | stabilin 2 |
| Stag1 | stromal antigen 1 |
| Stap1 | signal transducing adaptor family member 1 |
| Stard8 | START domain containing 8 |
| Stat1 | signal transducer and activator of transcription 1 |
| Stat3 | signal transducer and activator of transcription 3 |
| Stat5b | signal transducer and activator of transcription 5B |
| Stau2 | staufen (RNA binding protein) homolog 2 (Drosophila) |
| Stc1 | stanniocalcin 1 |
| Stc2 | stanniocalcin 2 |
| Stim1 | stromal interaction molecule 1 |
| Stim2 | stromal interaction molecule 2 |
| Stk17b | serine/threonine kinase 17b (apoptosis-inducing) |
| Stk24 | serine/threonine kinase 24 |
| Stk35 | serine/threonine kinase 35 |
| Stk38 | serine/threonine kinase 38 |
| Stk39 | serine/threonine kinase 39 |
| Stk4 | serine/threonine kinase 4 |
| Ston2 | stonin 2 |
| Stox2 | storkhead box 2 |
| Stpg4 | sperm tail PG rich repeat containing 4 |
| Stra6 | stimulated by retinoic acid gene 6 |
| Strn | striatin |
| Stx11 | syntaxin 11 |
| Stx6 | syntaxin 6 |
| Stx7 | syntaxin 7 |
| Stx8 | syntaxin 8 |
| Stxbp3 | syntaxin binding protein 3 |
| Stxbp5 | syntaxin binding protein 5 (tomosyn) |
| Stxbp6 | syntaxin binding protein 6 (amisyn) |
| Suclg1 | succinate-CoA ligase |
| Suclg2 | succinate-Coenzyme A ligase |
| Sugp2 | SURP and G patch domain containing 2 |
| Sugt1 | SGT1 |
| Sulf1 | sulfatase 1 |
| Sulf2 | sulfatase 2 |
| Sult3a1 | sulfotransferase family 3A |
| Sult6b2 | sulfotransferase family 6B |
| Sun1 | Sad1 and UNC84 domain containing 1 |
| Sun2 | Sad1 and UNC84 domain containing 2 |
| Supt20 | suppressor of Ty 20 |
| Susd1 | sushi domain containing 1 |
| Susd3 | sushi domain containing 3 |
| Susd5 | sushi domain containing 5 |
| Sv2b | synaptic vesicle glycoprotein 2 b |
| Sv2c | synaptic vesicle glycoprotein 2c |
| Svep1 | sushi |
| Syde1 | synapse defective 1 |
| Syde2 | synapse defective 1 |
| Syk | spleen tyrosine kinase |
| Syndig1 | synapse differentiation inducing 1 |
| Syndig1l | synapse differentiation inducing 1 like |
| Syne1 | spectrin repeat containing |
| Synj2 | synaptojanin 2 |
| Synpo2 | synaptopodin 2 |
| Sypl | synaptophysin-like protein |
| Syt1 | synaptotagmin I |
| Syt13 | synaptotagmin XIII |
| Syt16 | synaptotagmin XVI |
| Syt2 | synaptotagmin II |
| Syt6 | synaptotagmin VI |
| Syt9 | synaptotagmin IX |
| Tacc1 | transforming |
| Tacr1 | tachykinin receptor 1 |
| Taf3 | TATA-box binding protein associated factor 3 |
| Tanc1 | tetratricopeptide repeat |
| Tango6 | transport and golgi organization 6 |
| Tank | TRAF family member-associated Nf-kappa B activator |
| Tas2r143 | taste receptor |
| Tasp1 | taspase |
| Tbata | thymus |
| Tbc1d1 | TBC1 domain family |
| Tbc1d10a | TBC1 domain family |
| Tbc1d16 | TBC1 domain family |
| Tbc1d22a | TBC1 domain family |
| Tbc1d22b | TBC1 domain family |
| Tbc1d2b | TBC1 domain family |
| Tbc1d4 | TBC1 domain family |
| Tbc1d9 | TBC1 domain family |
| Tbcel | tubulin folding cofactor E-like |
| Tbl1xr1 | transducin (beta)-like 1X-linked receptor 1 |
| Tbr1 | T-box brain gene 1 |
| Tbx19 | T-box 19 |
| Tbx20 | T-box 20 |
| Tbx3 | T-box 3 |
| Tcaf1 | TRPM8 channel-associated factor 1 |
| Tcerg1 | transcription elongation regulator 1 (CA150) |
| Tcf12 | transcription factor 12 |
| Tcf15 | transcription factor 15 |
| Tcf20 | transcription factor 20 |
| Tcf25 | transcription factor 25 (basic helix-loop-helix) |
| Tcf4 | transcription factor 4 |
| Tcf7 | transcription factor 7, T cell specific |
| Tcf7l2 | transcription factor 7 like 2 |
| Tcof1 | treacle ribosome biogenesis factor 1 |
| Tcp10b | t-complex protein 10b |
| Tctex1d1 | Tctex1 domain containing 1 |
| Tdp1 | tyrosyl-DNA phosphodiesterase 1 |
| Tdrd3 | tudor domain containing 3 |
| Tdrd6 | tudor domain containing 6 |
| Tdrp | testis development related protein |
| Tead1 | TEA domain family member 1 |
| Tec | tec protein tyrosine kinase |
| Tecpr2 | tectonin beta-propeller repeat containing 2 |
| Teddm3 | transmembrane epididymal family member 3 |
| Tef | thyrotroph embryonic factor |
| Tenm4 | teneurin transmembrane protein 4 |
| Terf2ip | telomeric repeat binding factor 2 |
| Tesk2 | testis-specific kinase 2 |
| Tet1 | tet methylcytosine dioxygenase 1 |
| Tet2 | tet methylcytosine dioxygenase 2 |
| Tet3 | tet methylcytosine dioxygenase 3 |
| Tex26 | testis expressed 26 |
| Tex29 | testis expressed 29 |
| Tex9 | testis expressed gene 9 |
| Tfap2c | transcription factor AP-2 |
| Tfdp1 | transcription factor Dp 1 |
| Tfdp2 | transcription factor Dp 2 |
| Tgfa | transforming growth factor alpha |
| Tgfbr2 | transforming growth factor |
| Tgfbr3 | transforming growth factor |
| Tgm4 | transglutaminase 4 (prostate) |
| Thada | thyroid adenoma associated |
| Thap12 | THAP domain containing 12 |
| Thap4 | THAP domain containing 4 |
| Themis | thymocyte selection associated |
| Themis2 | thymocyte selection associated family member 2 |
| Thpo | thrombopoietin |
| Thsd7a | thrombospondin |
| Tiam1 | T cell lymphoma invasion and metastasis 1 |
| Timd2 | T cell immunoglobulin and mucin domain containing 2 |
| Tiprl | TIP41 |
| Tjp2 | tight junction protein 2 |
| Tle3 | transducin-like enhancer of split 3 |
| Tle4 | transducin-like enhancer of split 4 |
| Tlk1 | tousled-like kinase 1 |
| Tlr1 | toll-like receptor 1 |
| Tlr3 | toll-like receptor 3 |
| Tlr5 | toll-like receptor 5 |
| Tlr7 | toll-like receptor 7 |
| Tlr8 | toll-like receptor 8 |
| Tm2d1 | TM2 domain containing 1 |
| Tm4sf5 | transmembrane 4 superfamily member 5 |
| Tmc1 | transmembrane channel-like gene family 1 |
| Tmc3 | transmembrane channel-like gene family 3 |
| Tmc8 | transmembrane channel-like gene family 8 |
| Tmcc1 | transmembrane and coiled coil domains 1 |
| Tmeff1 | transmembrane protein with EGF-like and two follistatin-like domains 1 |
| Tmem110 | transmembrane protein 110 |
| Tmem116 | transmembrane protein 116 |
| Tmem117 | transmembrane protein 117 |
| Tmem121 | transmembrane protein 121 |
| Tmem123 | transmembrane protein 123 |
| Tmem131 | transmembrane protein 131 |
| Tmem132c | transmembrane protein 132C |
| Tmem132e | transmembrane protein 132E |
| Tmem14a | transmembrane protein 14A |
| Tmem150c | transmembrane protein 150C |
| Tmem161b | transmembrane protein 161B |
| Tmem17 | transmembrane protein 17 |
| Tmem178 | transmembrane protein 178 |
| Tmem18 | transmembrane protein 18 |
| Tmem182 | transmembrane protein 182 |
| Tmem184b | transmembrane protein 184b |
| Tmem192 | transmembrane protein 192 |
| Tmem2 | transmembrane protein 2 |
| Tmem200a | transmembrane protein 200A |
| Tmem217 | transmembrane protein 217 |
| Tmem229a | transmembrane protein 229A |
| Tmem236 | transmembrane protein 236 |
| Tmem243 | transmembrane protein 243 |
| Tmem245 | transmembrane protein 245 |
| Tmem255b | transmembrane protein 255B |
| Tmem26 | transmembrane protein 26 |
| Tmem260 | transmembrane protein 260 |
| Tmem28 | transmembrane protein 28 |
| Tmem29 | transmembrane protein 29 |
| Tmem30b | transmembrane protein 30B |
| Tmem35a | transmembrane protein 35A |
| Tmem45a | transmembrane protein 45a |
| Tmem51 | transmembrane protein 51 |
| Tmem63b | transmembrane protein 63b |
| Tmem64 | transmembrane protein 64 |
| Tmem87b | transmembrane protein 87B |
| Tmem94 | transmembrane protein 94 |
| Tmod1 | tropomodulin 1 |
| Tmprss11a | transmembrane protease |
| Tmprss11e | transmembrane protease |
| Tmprss15 | transmembrane protease |
| Tmprss3 | transmembrane protease |
| Tmprss7 | transmembrane serine protease 7 |
| Tmsb10 | thymosin |
| Tnfaip3 | tumor necrosis factor |
| Tnfrsf11a | tumor necrosis factor receptor superfamily |
| Tnfrsf13b | tumor necrosis factor receptor superfamily |
| Tnfrsf19 | tumor necrosis factor receptor superfamily |
| Tnfsf11 | tumor necrosis factor (ligand) superfamily |
| Tnfsf18 | tumor necrosis factor (ligand) superfamily |
| Tnfsf4 | tumor necrosis factor (ligand) superfamily |
| Tnfsf8 | tumor necrosis factor (ligand) superfamily |
| Tnik | TRAF2 and NCK interacting kinase |
| Tnip1 | TNFAIP3 interacting protein 1 |
| Tnip2 | TNFAIP3 interacting protein 2 |
| Tnip3 | TNFAIP3 interacting protein 3 |
| Tnks | tankyrase |
| Tnn | tenascin N |
| Tnni2 | troponin I |
| Tnpo1 | transportin 1 |
| Tnrc6b | trinucleotide repeat containing 6b |
| Tnrc6c | trinucleotide repeat containing 6C |
| Tns1 | tensin 1 |
| Tns3 | tensin 3 |
| Tns4 | tensin 4 |
| Tob1 | transducer of ErbB-2.1 |
| Tom1l2 | target of myb1-like 2 (chicken) |
| Tomm20 | translocase of outer mitochondrial membrane 20 homolog (yeast) |
| Top1 | topoisomerase (DNA) I |
| Tor1aip2 | torsin A interacting protein 2 |
| Tox | thymocyte selection-associated high mobility group box |
| Tox2 | TOX high mobility group box family member 2 |
| Tox3 | TOX high mobility group box family member 3 |
| Tpbg | trophoblast glycoprotein |
| Tpk1 | thiamine pyrophosphokinase |
| Tprg | transformation related protein 63 regulated |
| Tpst1 | protein-tyrosine sulfotransferase 1 |
| Traf3 | TNF receptor-associated factor 3 |
| Tram1 | translocating chain-associating membrane protein 1 |
| Tram2 | translocating chain-associating membrane protein 2 |
| Trank1 | tetratricopeptide repeat and ankyrin repeat containing 1 |
| Trappc12 | trafficking protein particle complex 12 |
| Trat1 | T cell receptor associated transmembrane adaptor 1 |
| Treml2 | triggering receptor expressed on myeloid cells-like 2 |
| Trerf1 | transcriptional regulating factor 1 |
| Trib1 | tribbles pseudokinase 1 |
| Trib2 | tribbles pseudokinase 2 |
| Tril | TLR4 interactor with leucine-rich repeats |
| Trim30d | tripartite motif-containing 30D |
| Trim36 | tripartite motif-containing 36 |
| Trim44 | tripartite motif-containing 44 |
| Trim55 | tripartite motif-containing 55 |
| Trio | triple functional domain (PTPRF interacting) |
| Trip12 | thyroid hormone receptor interactor 12 |
| Troap | trophinin associated protein |
| Trpc3 | transient receptor potential cation channel |
| Trpm1 | transient receptor potential cation channel |
| Trpm2 | transient receptor potential cation channel |
| Trpm3 | transient receptor potential cation channel |
| Trpm6 | transient receptor potential cation channel |
| Trpm8 | transient receptor potential cation channel |
| Trps1 | transcriptional repressor GATA binding 1 |
| Tsc22d1 | TSC22 domain family |
| Tsen2 | tRNA splicing endonuclease subunit 2 |
| Tsg101 | tumor susceptibility gene 101 |
| Tshz1 | teashirt zinc finger family member 1 |
| Tshz2 | teashirt zinc finger family member 2 |
| Tspan12 | tetraspanin 12 |
| Tspan13 | tetraspanin 13 |
| Tspan3 | tetraspanin 3 |
| Tspan5 | tetraspanin 5 |
| Tspan9 | tetraspanin 9 |
| Tssc1 | tumor suppressing subtransferable candidate 1 |
| Ttbk2 | tau tubulin kinase 2 |
| Ttc17 | tetratricopeptide repeat domain 17 |
| Ttc27 | tetratricopeptide repeat domain 27 |
| Ttc28 | tetratricopeptide repeat domain 28 |
| Ttc3 | tetratricopeptide repeat domain 3 |
| Ttc39b | tetratricopeptide repeat domain 39B |
| Ttc39c | tetratricopeptide repeat domain 39C |
| Ttc6 | tetratricopeptide repeat domain 6 |
| Ttc7 | tetratricopeptide repeat domain 7 |
| Ttc9 | tetratricopeptide repeat domain 9 |
| Ttl | tubulin tyrosine ligase |
| Ttll8 | tubulin tyrosine ligase-like family |
| Ttn | titin |
| Ttpa | tocopherol (alpha) transfer protein |
| Tuft1 | tuftelin 1 |
| Tulp4 | tubby like protein 4 |
| Tusc1 | tumor suppressor candidate 1 |
| Tusc3 | tumor suppressor candidate 3 |
| Tvp23a | trans-golgi network vesicle protein 23A |
| Twf1 | twinfilin actin binding protein 1 |
| Twist2 | twist basic helix-loop-helix transcription factor 2 |
| Txk | TXK tyrosine kinase |
| Txndc15 | thioredoxin domain containing 15 |
| Txnrd2 | thioredoxin reductase 2 |
| Ubac2 | ubiquitin associated domain containing 2 |
| Ubash3b | ubiquitin associated and SH3 domain containing |
| Ubc | ubiquitin C |
| Ube2cbp | ubiquitin-conjugating enzyme E2C binding protein |
| Ube2d2a | ubiquitin-conjugating enzyme E2D 2A |
| Ube2e1 | ubiquitin-conjugating enzyme E2E 1 |
| Ube2e2 | ubiquitin-conjugating enzyme E2E 2 |
| Ube4b | ubiquitination factor E4B |
| Ubp1 | upstream binding protein 1 |
| Ubr1 | ubiquitin protein ligase E3 component n-recognin 1 |
| Ubr2 | ubiquitin protein ligase E3 component n-recognin 2 |
| Ubr4 | ubiquitin protein ligase E3 component n-recognin 4 |
| Ubtd1 | ubiquitin domain containing 1 |
| Ubtd2 | ubiquitin domain containing 2 |
| Ufsp2 | UFM1-specific peptidase 2 |
| Ugcg | UDP-glucose ceramide glucosyltransferase |
| Ugt1a10 | UDP glycosyltransferase 1 family |
| Ugt1a6a | UDP glucuronosyltransferase 1 family |
| Ugt1a9 | UDP glucuronosyltransferase 1 family |
| Ulbp1 | UL16 binding protein 1 |
| Ulk4 | unc-51-like kinase 4 |
| Unc13a | unc-13 homolog A (C. elegans) |
| Unc45b | unc-45 myosin chaperone B |
| Unc5a | unc-5 netrin receptor A |
| Unc5c | unc-5 netrin receptor C |
| Uncx | UNC homeobox |
| Upf1 | UPF1 regulator of nonsense transcripts homolog (yeast) |
| Uprt | uracil phosphoribosyltransferase |
| Urad | ureidoimidazoline (2-oxo-4-hydroxy-4-carboxy-5) decarboxylase |
| Usb1 | U6 snRNA biogenesis 1 |
| Usf3 | upstream transcription factor family member 3 |
| Uso1 | USO1 vesicle docking factor |
| Usp3 | ubiquitin specific peptidase 3 |
| Usp33 | ubiquitin specific peptidase 33 |
| Usp35 | ubiquitin specific peptidase 35 |
| Usp4 | ubiquitin specific peptidase 4 (proto-oncogene) |
| Usp44 | ubiquitin specific peptidase 44 |
| Usp46 | ubiquitin specific peptidase 46 |
| Usp53 | ubiquitin specific peptidase 53 |
| Usp7 | ubiquitin specific peptidase 7 |
| Uspl1 | ubiquitin specific peptidase like 1 |
| Utp14b | UTP14B small subunit processome component |
| Utrn | utrophin |
| Uty | ubiquitously transcribed tetratricopeptide repeat gene |
| Uvrag | UV radiation resistance associated gene |
| Vangl1 | vang-like 1 (van gogh |
| Vapb | vesicle-associated membrane protein |
| Vat1l | vesicle amine transport protein 1 like |
| Vav2 | vav 2 oncogene |
| Vav3 | vav 3 oncogene |
| Vax1 | ventral anterior homeobox 1 |
| Vcl | vinculin |
| Vdac1 | voltage-dependent anion channel 1 |
| Veph1 | ventricular zone expressed PH domain-containing 1 |
| Vezf1 | vascular endothelial zinc finger 1 |
| Vgll1 | vestigial like family member 1 |
| Vgll4 | vestigial like family member 4 |
| Vmn1r20 | vomeronasal 1 receptor 20 |
| Vmn2r92 | vomeronasal 2 |
| Vmn2r99 | vomeronasal 2 |
| Vps13b | vacuolar protein sorting 13B |
| Vps13c | vacuolar protein sorting 13C |
| Vps41 | VPS41 HOPS complex subunit |
| Vrk1 | vaccinia related kinase 1 |
| Vrk2 | vaccinia related kinase 2 |
| Vstm2l | V-set and transmembrane domain containing 2-like |
| Vtcn1 | V-set domain containing T cell activation inhibitor 1 |
| Vti1a | vesicle transport through interaction with t-SNAREs 1A |
| Vwa3a | von Willebrand factor A domain containing 3A |
| Vwa8 | von Willebrand factor A domain containing 8 |
| Vwc2 | von Willebrand factor C domain containing 2 |
| Wasf1 | WAS protein family |
| Wasf2 | WAS protein family |
| Wasf3 | WAS protein family |
| Washc4 | WASH complex subunit 4 |
| Wbp1l | WW domain binding protein 1 like |
| Wdfy1 | WD repeat and FYVE domain containing 1 |
| Wdfy3 | WD repeat and FYVE domain containing 3 |
| Wdr11 | WD repeat domain 11 |
| Wdr41 | WD repeat domain 41 |
| Wdr46 | WD repeat domain 46 |
| Wdr59 | WD repeat domain 59 |
| Wdr7 | WD repeat domain 7 |
| Wdr78 | WD repeat domain 78 |
| Wdr82 | WD repeat domain containing 82 |
| Wdr89 | WD repeat domain 89 |
| Wdr91 | WD repeat domain 91 |
| Wdr92 | WD repeat domain 92 |
| Wfdc15b | WAP four-disulfide core domain 15B |
| Wfdc2 | WAP four-disulfide core domain 2 |
| Wfs1 | wolframin ER transmembrane glycoprotein |
| Whrn | whirlin |
| Wif1 | Wnt inhibitory factor 1 |
| Wipf1 | WAS/WASL interacting protein family |
| Wipf3 | WAS/WASL interacting protein family |
| Wipi1 | WD repeat domain |
| Wisp1 | WNT1 inducible signaling pathway protein 1 |
| Wnk3 | WNK lysine deficient protein kinase 3 |
| Wnt16 | wingless-type MMTV integration site family |
| Wnt3 | wingless-type MMTV integration site family |
| Wnt5b | wingless-type MMTV integration site family |
| Wnt9b | wingless-type MMTV integration site family |
| Wsb2 | WD repeat and SOCS box-containing 2 |
| Wscd1 | WSC domain containing 1 |
| Wt1os | Wilms tumor 1 homolog |
| Wwc1 | WW |
| Wwc2 | WW |
| Wwox | WW domain-containing oxidoreductase |
| Wwp2 | WW domain containing E3 ubiquitin protein ligase 2 |
| Wwtr1 | WW domain containing transcription regulator 1 |
| Xcl1 | chemokine (C motif) ligand 1 |
| Xdh | xanthine dehydrogenase |
| Xkr4 | X-linked Kx blood group related 4 |
| Xkrx | X-linked Kx blood group related |
| Xpa | xeroderma pigmentosum |
| Xpnpep2 | X-prolyl aminopeptidase (aminopeptidase P) 2 |
| Xpo4 | exportin 4 |
| Xpo6 | exportin 6 |
| Xrcc6 | X-ray repair complementing defective repair in Chinese hamster cells 6 |
| Xylt1 | xylosyltransferase 1 |
| Ylpm1 | YLP motif containing 1 |
| Ypel5 | yippee-like 5 (Drosophila) |
| Ywhaz | tyrosine 3-monooxygenase/tryptophan 5-monooxygenase activation protein |
| Zadh2 | zinc binding alcohol dehydrogenase |
| Zbed4 | zinc finger |
| Zbtb1 | zinc finger and BTB domain containing 1 |
| Zbtb18 | zinc finger and BTB domain containing 18 |
| Zbtb20 | zinc finger and BTB domain containing 20 |
| Zbtb9 | zinc finger and BTB domain containing 9 |
| Zc3h12b | zinc finger CCCH-type containing 12B |
| Zc3h12c | zinc finger CCCH type containing 12C |
| Zcchc10 | zinc finger |
| Zcchc14 | zinc finger |
| Zcchc2 | zinc finger |
| Zcchc24 | zinc finger |
| Zcchc9 | zinc finger |
| Zcwpw1 | zinc finger |
| Zdbf2 | zinc finger |
| Zdhhc13 | zinc finger |
| Zdhhc14 | zinc finger |
| Zdhhc15 | zinc finger |
| Zdhhc17 | zinc finger |
| Zeb1 | zinc finger E-box binding homeobox 1 |
| Zeb2 | zinc finger E-box binding homeobox 2 |
| Zfand3 | zinc finger |
| Zfand5 | zinc finger |
| Zfand6 | zinc finger |
| Zfhx3 | zinc finger homeobox 3 |
| Zfp120 | zinc finger protein 120 |
| Zfp148 | zinc finger protein 148 |
| Zfp2 | zinc finger protein 2 |
| Zfp236 | zinc finger protein 236 |
| Zfp24 | zinc finger protein 24 |
| Zfp277 | zinc finger protein 277 |
| Zfp281 | zinc finger protein 281 |
| Zfp286 | zinc finger protein 286 |
| Zfp316 | zinc finger protein 316 |
| Zfp346 | zinc finger protein 346 |
| Zfp354b | zinc finger protein 354B |
| Zfp365 | zinc finger protein 365 |
| Zfp366 | zinc finger protein 366 |
| Zfp36l1 | zinc finger protein 36 |
| Zfp385b | zinc finger protein 385B |
| Zfp407 | zinc finger protein 407 |
| Zfp438 | zinc finger protein 438 |
| Zfp449 | zinc finger protein 449 |
| Zfp462 | zinc finger protein 462 |
| Zfp507 | zinc finger protein 507 |
| Zfp521 | zinc finger protein 521 |
| Zfp53 | zinc finger protein 53 |
| Zfp532 | zinc finger protein 532 |
| Zfp534 | zinc finger protein 534 |
| Zfp54 | zinc finger protein 54 |
| Zfp57 | zinc finger protein 57 |
| Zfp592 | zinc finger protein 592 |
| Zfp60 | zinc finger protein 60 |
| Zfp608 | zinc finger protein 608 |
| Zfp618 | zinc finger protein 618 |
| Zfp619 | zinc finger protein 619 |
| Zfp629 | zinc finger protein 629 |
| Zfp64 | zinc finger protein 64 |
| Zfp644 | zinc finger protein 644 |
| Zfp658 | zinc finger protein 658 |
| Zfp667 | zinc finger protein 667 |
| Zfp677 | zinc finger protein 677 |
| Zfp697 | zinc finger protein 697 |
| Zfp704 | zinc finger protein 704 |
| Zfp706 | zinc finger protein 706 |
| Zfp746 | zinc finger protein 746 |
| Zfp800 | zinc finger protein 800 |
| Zfp820 | zinc finger protein 820 |
| Zfp825 | zinc finger protein 825 |
| Zfp827 | zinc finger protein 827 |
| Zfp839 | zinc finger protein 839 |
| Zfp846 | zinc finger protein 846 |
| Zfp862-ps | zinc finger protein 862 |
| Zfp879 | zinc finger protein 879 |
| Zfp930 | zinc finger protein 930 |
| Zfp950 | zinc finger protein 950 |
| Zfp976 | zinc finger protein 976 |
| Zfp982 | zinc finger protein 982 |
| Zfy1 | zinc finger protein 1 |
| Zfyve28 | zinc finger |
| Zgrf1 | zinc finger |
| Zhx2 | zinc fingers and homeoboxes 2 |
| Zkscan16 | zinc finger with KRAB and SCAN domains 16 |
| Zkscan3 | zinc finger with KRAB and SCAN domains 3 |
| Zmat4 | zinc finger |
| Zmiz1 | zinc finger |
| Zmpste24 | zinc metallopeptidase |
| Zmym6 | zinc finger |
| Zmynd8 | zinc finger |
| Znrf1 | zinc and ring finger 1 |
| Znrf2 | zinc and ring finger 2 |
| Znrf3 | zinc and ring finger 3 |
| Zswim2 | zinc finger SWIM-type containing 2 |
| Zswim6 | zinc finger SWIM-type containing 6 |
| Zzef1 | zinc finger |
| Zzz3 | zinc finger |
