# Supplementary material for: The histone methyltransferase EZH2 primes the early differentiation of follicular helper T cells during acute viral infection
Source: Cell Mol Immunol. 2019 Mar 6;17(3):247–60. doi: 10.1038/s41423-019-0219-z (PMC7052164; doi:10.1038/s41423-019-0219-z)
Supplement: Supplementary file 5 — Supplementary table 3 [file 41423_2019_219_MOESM5_ESM.docx]

Supplementary Table 3. Antibodies used in flow cytometry

| Antibody target/Reagent | Clone/Cat. No. | Dilution | Provider |
| --- | --- | --- | --- |
| CD4 | RM4-5 | 1:200 | Biolegend |
| CD19 | eBio1D3 | 1:100 | Biolegend |
| CD25 | PC61.5 | 1:100 | Biolegend |
| CD44 | IM7 | 1:100 | eBioscience |
| CD45.1 | A20 | 1:100 | Biolegend |
| CD45.2 | 104 | 1:100 | Biolegend |
| FAS | JO2 | 1:100 | BD Biosciences |
| SLAM | TC15-12F12.2 | 1:100 | Biolegend |
| PD-1 | RMP1-30 | 1:100 | Biolegend |
| Purified Rat Anti-Mouse CXCR5 | 2G8 | 1:100 | BD Biosciences |
| EZH2 | 562478 | 1:50 | BD Biosciences |
| H3K27me3 | 9733S | 1:200 | Cell Signaling Technology |
| ICOS | 7E.17G9 | 1:100 | Biolegend |
| T-bet | 4B10 | 1:100 | Biolegend |
| Foxp3 | FJK-16s | 1:100 | eBioscience |
| PNA | FL-1071 | 1:500 | Vector Labs |
| TCF-1 | C46C7 | 1:200 | Cell Signaling Technology |
| Bcl-6 | K112-91 | 1:20 | BD Biosciences |
| CD138 | 281-2 | 1:100 | BD Biosciences |
| CD45R(B220) | RA3-6B2 | 1:100 | eBioscience |
| Strepavidin | 25-4317-82 | 1:200 | eBioscience |
| Alexa Fluor (R) 647 anti-rabbit polyclonal IgG Fab2 | 4414S | 1:500 | Cell Signaling Technology |
| Donkey anti-Rabbit Polyclonal IgG (H+L) Highly Cross-Adsorbed Secondary Antibody | A-21206 | 1:500 | Thermofisher |
| Biotin-SP (long spacer) AffiniPure Goat Anti-Rat IgG (H+L) | 112-066-003 | 1:200 | Jackson Immunoresearch |
| LIVE/DEAD Fixable Near-IR Dead Cell Stain Kit | L10199 | 1:200 | Life Technologies |
