# Supplementary material for: The histone methyltransferase EZH2 primes the early differentiation of follicular helper T cells during acute viral infection
Source: Cell Mol Immunol. 2019 Mar 6;17(3):247–60. doi: 10.1038/s41423-019-0219-z (PMC7052164; doi:10.1038/s41423-019-0219-z)
Supplement: Supplementary file 6 — Supplementary table 4 [file 41423_2019_219_MOESM6_ESM.docx]

Supplementary Table 4. Primers used in quantitative RT-PCR.

| ***Primers for gene expression analysis*** | | |
| --- | --- | --- |
| Gene symbol | 5' primer | 3' primer |
| *Bcl6* | 5'-agacgcacagtgacaaacca | 5'-agtgtgggtcttcaggttgg |
| *Tcf7* | 5'-caatctgctcatgccctacc | 5'-cttgcttctggctgatgtcc |
| *Lef1* | 5’-tgagtgcacgctaaaggaga | 3’-ctgaccagcctggataaagc |
| *Ascl2* | 5'-tactcgtcggaggaaagcag | 5'-acccagggatgcagcttag |
| *Batf* | 5'-ctggcaaacaggactcatctg | 5'-gggtgtcggctttctgtgtc |
| *Id3* | 5'-atctcccgatccagacagc | 5'-gagagagggtcccagagtcc |
| *Cxcr5* | 5'-catgggctccatcacataca | 5'-ggcatgaataccgccttaaa |
| *Icos* | 5'-tgccgtgtctttgtcttctg | 5'-cttcccttggtcttggtgag |
| *Il21* | 5’-cgcctcctgattagacttcg | 5’-aaaacaggcaaaagctgcat |
| *Sh2d1a* | 5’-ggcttctgaaacgctgagat | 5’-tcgagtgtcccagacagaaa |
